# Suzuki–Miyaura cross-coupling of amides and esters at room temperature: correlation with barriers to rotation around C–N and C–O bonds†
†Electronic supplementary information (ESI) available: Experimental details and characterization data. See DOI: 10.1039/c7sc02692g



**DOI:** 10.1039/c7sc02692g

**Published:** 2017-08-01

**Authors:** Peng Lei, Guangrong Meng, Shicheng Shi, Yun Ling, Jie An, Roman Szostak, Michal Szostak

**Affiliations:** a Department of Applied Chemistry , College of Science , China Agricultural University , Beijing 100193 , China; b Department of Chemistry , Rutgers University , 73 Warren Street , Newark , NJ 07102 , USA . Email: michal.szostak@rutgers.edu; c Department of Chemistry , Wroclaw University , F. Joliot-Curie 14 , Wroclaw , 50-383 , Poland

## Abstract

The Suzuki–Miyaura cross-coupling has been widely recognized as one of the most important methods for the construction of C–C bonds. However, in contrast to traditional aryl halide or pseudohalide electrophiles, coupling reactions with unactivated C–N and C–O electrophiles have proven significantly more challenging. Here we report the first general palladium-catalyzed Suzuki–Miyaura cross-coupling of both common amides and aryl esters through the selective cleavage of the C–N and C–O bonds under exceedingly mild conditions. Notably, for the first time we demonstrate selective C(acyl)–N and C(acyl)–O cleavage/cross-coupling under the same reaction conditions. The reaction uses a commercially available, bench-stable and operationally-convenient (η^3^-1-*t*-Bu-indenyl)Pd(IPr)(Cl) precatalyst. Furthermore, we demonstrate that the reactivity of generic amides and aryl esters can be correlated with barriers to isomerization around the C(acyl)–X (X = N, O) bond, thus providing a blueprint for the development of a broad range of novel coupling reactions of ester and amide electrophiles by the selective activation of C–O and C–N bonds.

## Abstract 2

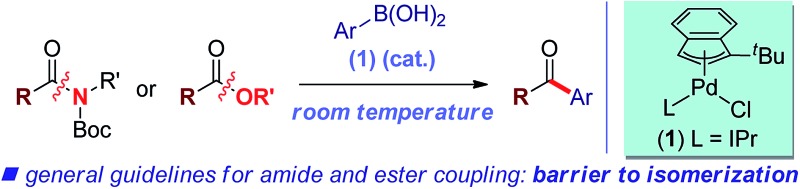
We report the first general palladium-catalyzed Suzuki–Miyaura cross-coupling of both common amides and aryl esters through the selective cleavage of the C–N and C–O bonds at ambient temperature.

## Introduction

Transition-metal-catalyzed cross-coupling reactions have become a central transformation in organic synthesis.^1^ Although cross-coupling reactions of aryl halides and pseudohalides have been extensively developed,^2^ chemical methods using unconventional electrophiles have been more challenging to develop.^3^ Over the past five years, strategies for the cross-coupling of unactivated aryl ester and amide electrophiles using Ni-, Pd- and Rh-catalysis have been reported.^4–7^ However, the development of practical methods by selective C(acyl)–O and C(acyl)–N scission in common esters and amides remains an elusive goal in organic synthesis.

**Fig. 1:**
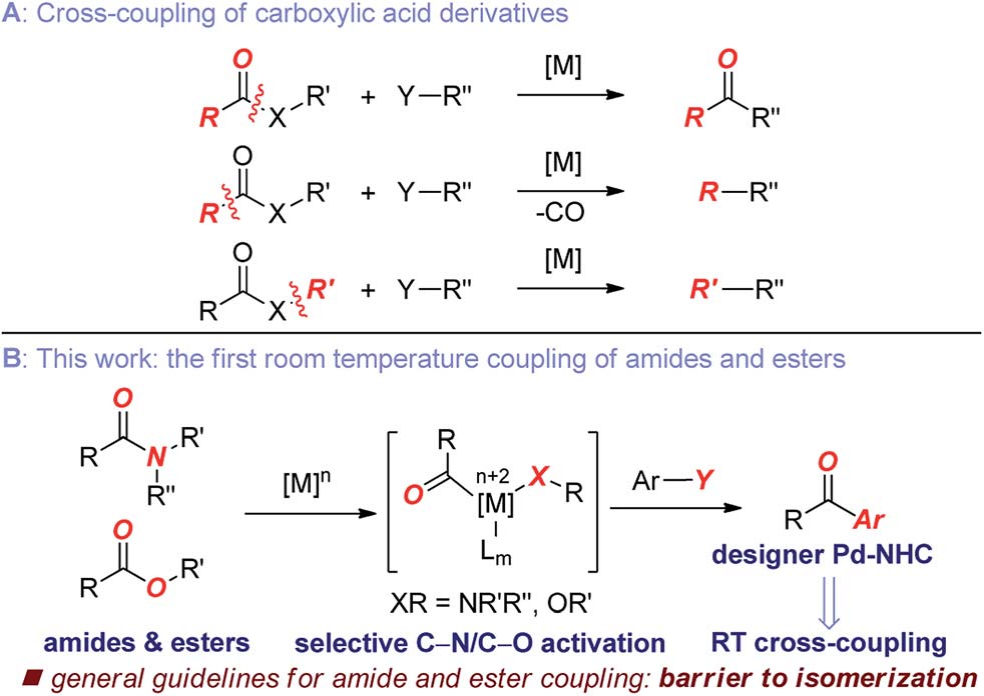
(A) Cross-coupling of carboxylic acid derivatives. (B) The first general coupling of amides and esters at room temperature enabled by designer Pd–NHC catalysis (this work).

The key challenge in the cross-coupling of unactivated esters and amides is slow oxidative addition of the C(acyl)–X (X = N, O) bond to the metal.^8^ Accordingly, these reactions typically require elevated temperatures, leading to problems associated with low reactivity and reaction selectivity of acyl-metal intermediates through direct coupling and decarbonylative pathways (Fig. 1A).^4–7^

Here we demonstrate the selective C(acyl)–N and C(acyl)–O cleavage/cross-coupling of common amides and esters by operationally-convenient Pd–NHC precatalyst at room temperature. The following features of our study are notable:

(1) We describe the first room temperature Suzuki coupling of both common esters and amides by O–C/N–C bond activation. This is by far the most active catalyst identified to date. The current-state-of-the-art for ester coupling is 90 °C, while for amides 60 °C. The practicality and generality of method supersede the current methods.

(2) We establish the first Suzuki coupling of both common esters and amides by selective O–C/N–C bond activation under the same catalytic reaction conditions. This concept could enable the productive engagement of the acyl and aryl coupling mechanisms from both types of these unconventional electrophiles and avoid restriction to a particular acylmetal precursor.

(3) We provide guidelines for cross-coupling of amide/ester electrophiles by showing that the reactivity can be correlated with barriers to isomerization around the C(acyl)–X bond.

These innovative concepts in the cross-coupling of amide/ester acyl electrophiles demonstrate how simple and readily available amides and esters can be systematically used as coupling partners in transition metal catalysis.

There is a strong impetus to develop selective coupling reactions of bench-stable unactivated amide and ester electrophiles due to the prevalence of these precursors in modern organic synthesis.^9^ Furthermore, amides and esters are traditionally derived from different pool of precursors than aryl halides, phenols and anilines.^10^ Additionally, amides and esters are robust, easy to handle, and typically inert to a variety of reaction conditions allowing for ring prefunctionalization.^11^ Developing a single manifold for C(acyl)–O and C(acyl)–N cleavage could enable the productive engagement of the acyl and aryl coupling mechanisms from both types of these unconventional electrophiles and avoid restriction to a particular acylmetal precursor. Methods that engage common amides and esters in coupling manifolds under mild conditions are absent.^4–7^

Here we establish the cross-coupling of common amides and aryl esters through the selective cleavage of the C–N and C–O bonds that proceeds under exceedingly mild conditions (Fig. 1B). These studies represent the first example of a room temperature Suzuki–Miyaura coupling of common amide and ester electrophiles.^4–7^ As a result of these studies, we report experimental evidence showing that (1) the selective C(acyl)–N and C(acyl)–O cleavage/cross-coupling can proceed under the same reaction conditions, (2) the reactivity of generic amides and aryl esters can be correlated with barriers to isomerization around the C(acyl)–X (X = N, O) bond, (3) the present barrier to chemoselective carbon–carbon bond forming reactivity of unactivated esters and amides by C(acyl)–X (X = N, O) bond cleavage is located at *ca.* 10 kcal mol^–1^ bond isomerization. Collectively, our studies provide a blueprint for the development of a broad range of novel coupling reactions of unconventional ester and amide electrophiles by the selective cleavage of C–O and C–N bonds.

## Results and discussion

Recently, our laboratory introduced a new amide bond activation mode by ground-state destabilization.^4*a*^ The seminal studies by Garg and co-workers have demonstrated that classical acyl cross-coupling pathways can be accessed from common amides.^7*a*^ With the goal of further expanding the manifold of amide functionalization, we anticipated that σ donor nucleophilic N-heterocyclic carbene (NHC) ligands could provide rapid access to acylmetal intermediates from amides using practical palladium catalysis.^7m,12^ Our studies focused on the identification of a suitable Pd–NHC precatalyst capable of facilitating oxidative addition at ambient conditions. We established that a commercially available, bench-stable and operationally-convenient (η^3^-1-*t*-Bu-indenyl)Pd(IPr)(Cl) (**1**), developed by Hazari and co-workers,^13^ is an effective catalyst for the coupling of common amides and esters with boronic acids at room temperature (eqn (1)).1
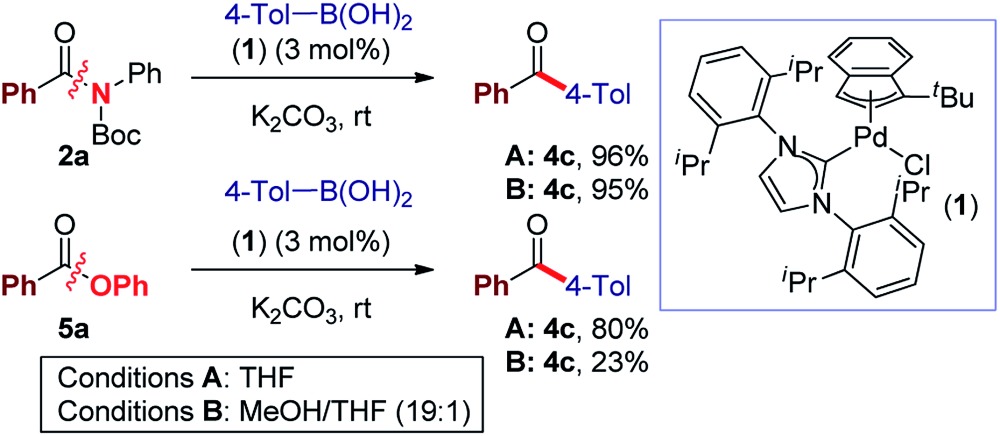



Elegant studies have shown that a major advantage of (η^3^-indenyl)Pd(IPr)(Cl) complexes stems from preventing the formation of the catalytically inactive Pd(i) dimer, and fast reduction of Pd(ii) to Pd(0), depending on reaction conditions.^14^ The high reactivity of these complexes results from maintaining the optimal 1 : 1 Pd to ligand ratio.^15^ The indenyl complex (**1**) was screened as a catalyst in the Suzuki–Miyaura cross-coupling of N-Boc activated amide **2a** with 4-tolylboronic acid (eqn (1)). Note that N-Boc activated amides are readily prepared by selective *N-tert*-butoxycarbonyl activation, which provides a general method for the cross-coupling of common2
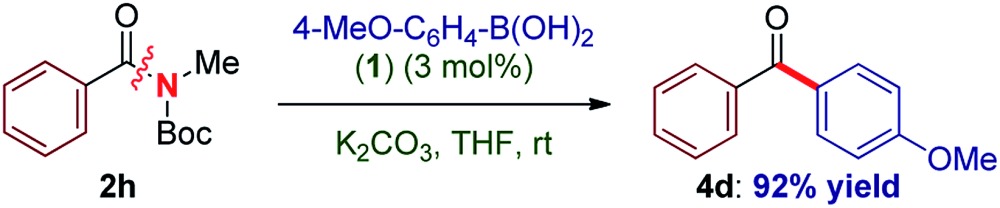

3
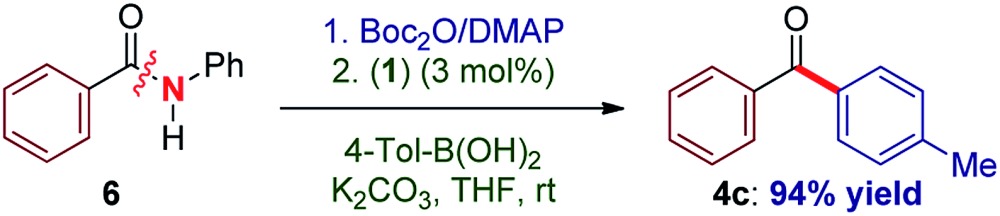
secondary amides.^4a,b^ We postulate that N-Boc activated amides represent the most synthetically useful family of amide electrophiles for cross-coupling.^7^ Under optimal conditions, the cross-coupling of **2a** proceeded in excellent yield using both heterogeneous (THF, 96% yield) and homogeneous (*i.e.* the base is either in or out of solution) (MeOH : THF, 19 : 1, 95% yield) reaction conditions^13^ (K_2_CO_3_, 4.5 equiv.) at room temperature (eqn (1)). Furthermore, coupling of aryl ester **5a** with 4-tolylboronic acid afforded the biaryl ketone product in 80% yield (THF, K_2_CO_3_, 7.2 equiv.). Interestingly, the use of homogeneous conditions with **5a** was ineffective (23% yield) (eqn (1)). Although detailed mechanistic studies are beyond the scope of this report, based on the elegant studies by Hazari, Nolan and others, the mechanism of activation involves rapid reduction from Pd(ii) to Pd(0) under both types of conditions.^14*b*^ Other bases (KOH, K_3_PO_4_, KF) and solvents (dioxane, toluene) were screened, with THF and K_2_CO_3_ providing the optimum results.

With the optimal reaction conditions in hand, the scope of the process was surveyed. As shown in Table 1, this method proceeds at room temperature with an extensive range of sterically and electronically differentiated boronic acids. The coupling using neutral (entry 1), sterically-hindered (entry 2), electron-rich (entries 3–4), electron-withdrawing (entry 5) and fluoro-containing (entry 6) boronic acids proceeded in excellent yields. Next, the scope of the amide component was examined (Table 1). As shown, a broad range of electrophilic amide acceptors serves as effective acyl precursors in this coupling. Notably, sterically-demanding (entry 7), electron-rich (entry 8), electron-deficient (entries 9–10), fluorine-containing (entry 11) and electron-rich, heterocyclic amides conjugated at the 2-position (entry 12) proved highly effective in the reaction. For comparison, the coupling was conducted using aryl ester **5a**, providing the biaryl ketone products in high to excellent yields at room temperature (Table 2, entries 1–6). Importantly, the scope of the ester component is also broad, furnishing high to excellent yields in the coupling at room temperature (Table 2, entries 7–12). Comparison of (**1**) with (η^3^-cinnamyl)Pd(IPr)(Cl) (Cin-IPr)^7m,17^ demonstrated that (**1**) significantly outperforms Cin-IPr in the coupling (see ESI†). The biaryl ketone motif is a central component in the production of a wide range of pharmaceuticals, natural products and organic materials.^16^ Boronic acid is used in excess. Transmetallation is generally considered as the rate-determining step in Suzuki–Miyaura couplings using Pd–NHC.^15*b*^ While further optimization of reaction conditions are ongoing in our laboratory, we note that (1) under our optimized conditions the coupling using 2.0 equiv. of boronic acid proceeds in 96% and 49% yield for the amide and ester, respectively; (2) we determined that in the coupling of esters, aqueous K_2_CO_3_ could be used, leading to 93% yield with 2.0 equiv. of boronic acid.

**Table 1:** Cross-coupling of amides at 23 °C^*a*^ ^,^ ^*b*^

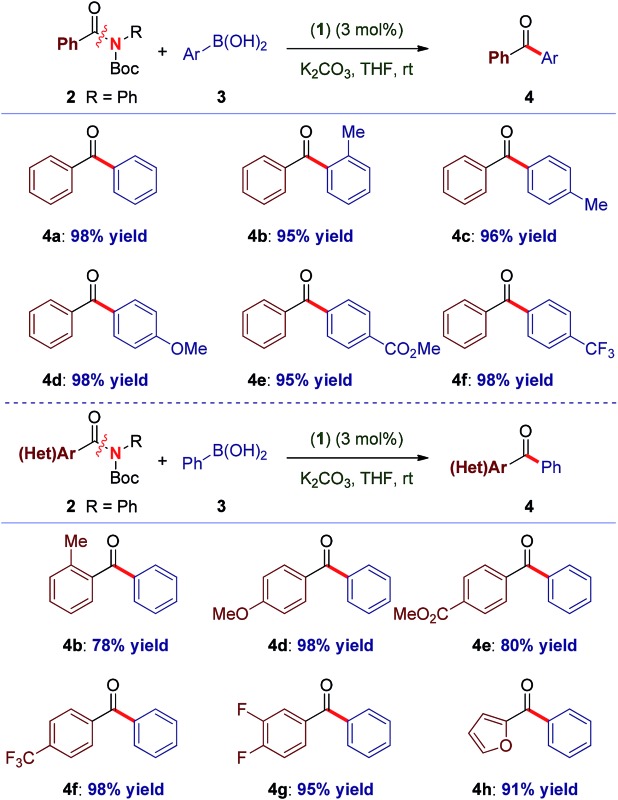

^*a*^Conditions: (**1**) (3 mol%), Ar-B(OH)_2_ (3.0 equiv.), K_2_CO_3_ (4.5 equiv.), THF (0.25 M), 23 °C, 15 h.

^*b*^Isolated yields. See ESI for full details.

**Table 2:** Cross-coupling of esters at 23 °C^*a*^ ^,^ ^*b*^

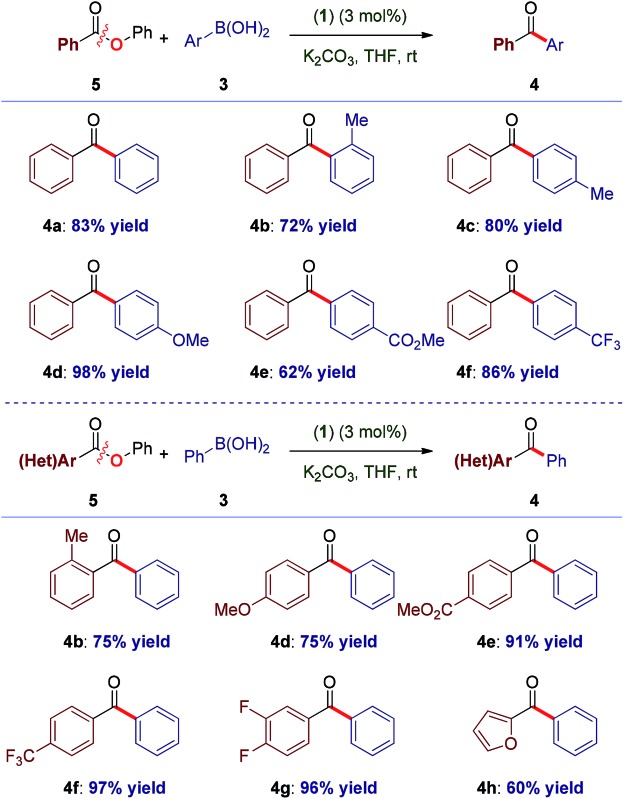

^*a*^Conditions: (**1**) (3 mol%), Ar-B(OH)_2_ (4.5 equiv.), K_2_CO_3_ (7.2 equiv.), THF (0.25 M), 23 °C, 15 h.

^*b*^Isolated yields. See ESI for full details.

Importantly, we have demonstrated that (1) there is no difference in the reaction efficiency when the reactions are performed using readily available commercial vials without pre-treatment; (2) since we are interested in developing cross-coupling of amides and esters of high operational simplicity, all reactions were set up on the bench-top (*cf.* glove-box); (3) we have demonstrated that adding water to the reaction (5 equiv.) leads to no decrease in yields in the cross-coupling of amides (95% yield) and esters (93% yield).

To our knowledge, these results provide (1) the first example of efficient cross-coupling of common amide and aryl ester electrophiles at room temperature using the versatile Pd-catalysis manifold,^1*c*^ (2) the first example of C–C coupling of amides and esters using a single catalyst system.^4–7^ Importantly, coupling of the challenging *N*-alkyl amides proceeded uneventfully (eqn (2)). It is noteworthy that one-pot N-Boc activation/cross-coupling furnishes the coupling product in excellent yield (eqn (3)), demonstrating the robustness of our protocol, and the potential of Pd–NHC precatalysts in amide coupling. To further illustrate the scope, we have systematically investigated the reaction efficiency when both components contain electron-rich and electron-withdrawing groups, examples when both components contain steric hindrance and aliphatic amide/ester electrophiles (Table 3). In all these examples good to excellent yields have been obtained. The present method surpasses other known protocols for coupling of amides/esters,^4–7^ and is performed at practical, room temperature conditions, which is not the case with any other coupling of these electrophiles reported to date. Note that, in addition to the prevalence of aryl esters in organic synthesis, aryl esters can be readily prepared from the corresponding carboxylic acids, benzylic alcohols, aldehydes and ketones,^6^ providing another advantage of this coupling manifold.

**Table 3:** Cross-coupling of amides and esters at 23 °C^*a*^

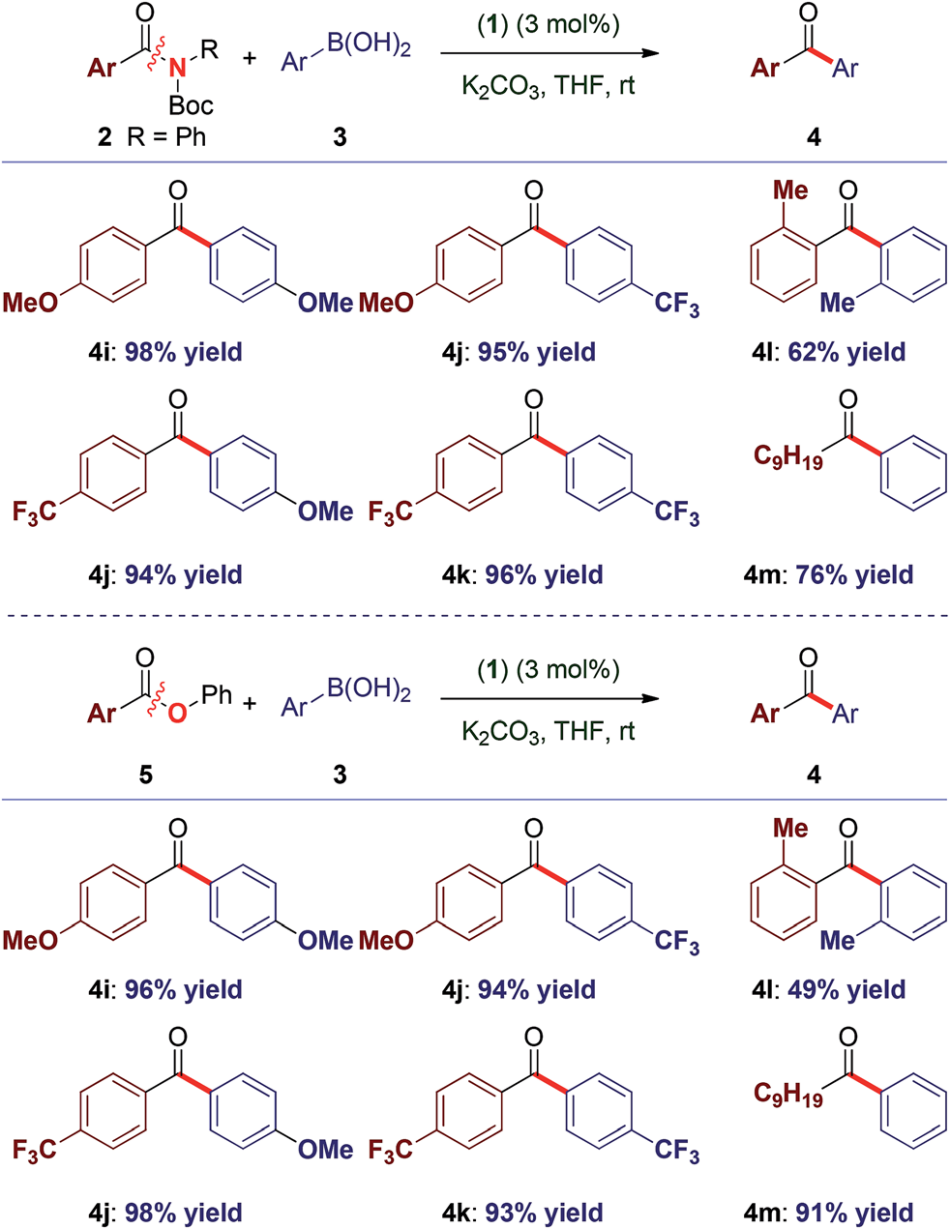

^*a*^See, Tables 1 and 2. See ESI for full details.

**Fig. 2:**
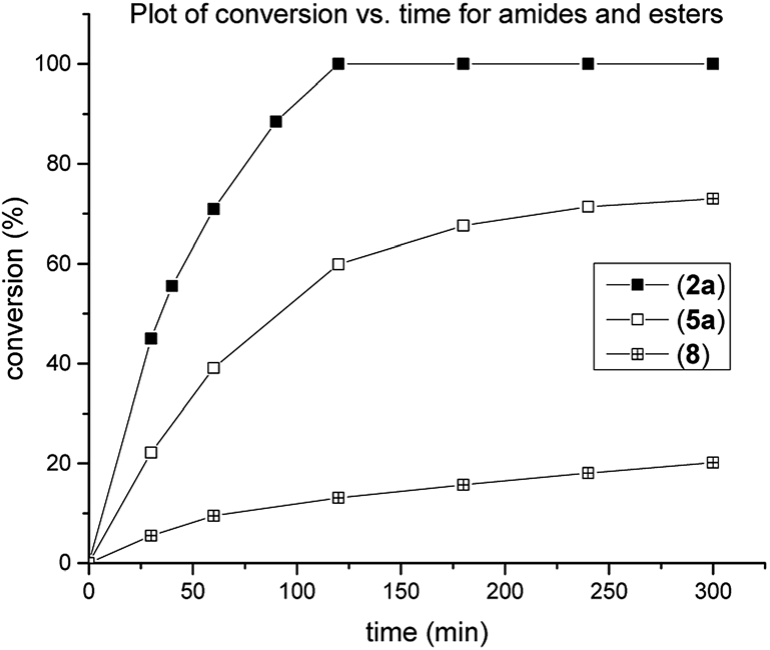
Kinetic profile in the Suzuki–Miyaura cross-coupling with 4-tolylboronic acid catalyzed by (**1**) (3 mol%) at room temperature. **2a**: *N*,*N*-Ph,Boc; **5a**: OPh; **8**: *N*,*N*-Ph,Ts.

Studies were conducted to gain insight into the catalytic reactivity of amide and ester electrophiles undergoing cross-coupling (Fig. 2). *N*-Ts/Ph benzamide^7^ was included for comparison. The study revealed significantly higher catalytic activity of **2a** than **5a** and *N*-Ts/Ph benzamide.

**Scheme 1:**
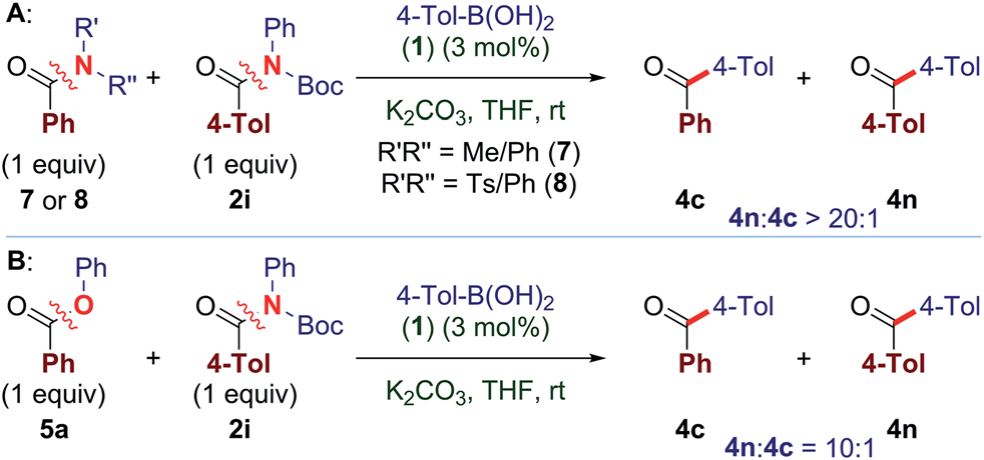
Selectivity in cross-coupling at 23 °C.

Furthermore, in competition studies, **2i** reacts preferentially over the less activated anilide (**7**) and *N*-Ts/Ph amide (**8**), giving full selectivity for the coupling of **2i** (Scheme 1A). Moreover, **2i** gives a synthetically useful selectivity over aryl ester **5a** (Scheme 1B). We also note that the alkyl ester bond remains intact under these conditions (**4e**).

The reactions indicate that there is a correlation between C–X acyl bond strength and reactivity. Classic studies by Liebman and Greenberg^18^ demonstrated that amidic resonance (*E*_r_ = 15–20 kcal mol^–1^, 
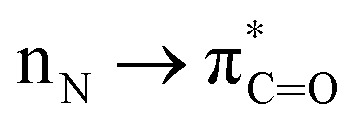
 conjugation in planar amides) correlates with the rotational barrier. Esters show higher resonance energy than amides (*e.g.*, MeCO_2_Me, *E*_r_ = 24 kcal mol^–1^); however, the rotational barrier is significantly lower as a result of maintaining resonance during the isomerization pathway.

**Scheme 2:**
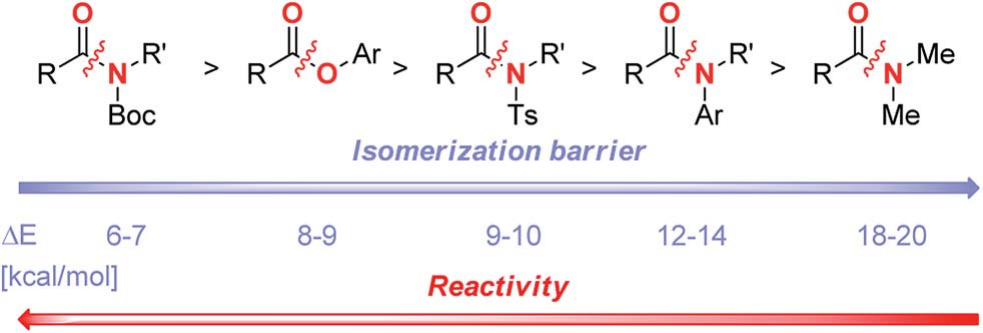
Reactivity scale of amides and esters in TM-catalyzed acyl C–N and acyl C–O coupling.

We have previously correlated the reactivity of amides with resonance energy by ground-state destabilization.^7*m*^ The present study allows to include the ester bond along the C–X (X = N, O) bond rotational pathway required for the effective metal insertion. Rotational barrier in PhCO_2_Ph has been quantified (9.3 kcal mol^–1^, see ESI†). Overall, our results provide strong support for a generalized reactivity scale of the amide and ester electrophiles for the formation of acyl-metal intermediates (Scheme 2).^19^ The present barrier to chemoselective carbon–carbon bond forming reactivity of unactivated esters and amides by C(acyl)–X (X = N, O) cleavage is located at *ca.* 10 kcal mol^–1^ bond isomerization.^20^ The development of improved catalyst systems will lay a foundation for a general application of amide and ester coupling in synthesis. Isomerization barrier is an important parameter that should be considered in acyl couplings.

## Conclusions

In summary, we demonstrated that Hazari's (η^3^-1-*t*-Bu-indenyl)Pd(IPr)(Cl) precatalyst shows unprecedented reactivity in the Suzuki–Miyaura cross-coupling of amides and esters by selective C(acyl)–N and C(acyl)–O cleavage. The potential of this catalyst system is illustrated by the first example of high yielding cross-coupling of amide and ester electrophiles at room temperature. This study demonstrates for the first time the selective C(acyl)–N and C(acyl)–O cleavage/C–C coupling under the same reaction conditions. The reactivity of generic amides and aryl esters can be correlated with barriers to isomerization around the C(acyl)–X bond (X = N, O). This study provides a blueprint for the development of a broad range of novel coupling reactions by avoiding restriction to a particular acylmetal precursor.
