# Supplementary material for: Suzuki–Miyaura cross-coupling of amides and esters at room temperature: correlation with barriers to rotation around C–N and C–O bonds
Source: Chem Sci. 2017 Aug 1;8(9):6525–30. doi: 10.1039/c7sc02692g (PMC5628478; doi:10.1039/c7sc02692g)
Supplement: Supplementary file 1 [file SC-008-C7SC02692G-s001.pdf]

Electronic Supplementary Material (ESI) for Chemical Science.
This journal is © The Royal Society of Chemistry 2017

Electronic Supplementary Information

Suzuki-Miyaura Cross-Coupling of Amides and Esters at Room Temperature: Correlation with Barriers to Rotation around C–N and C–O Bonds

Peng Lei,[†,‡] Guangrong Meng,[‡] Shicheng Shi,[‡] Yun Ling,[†] Jie An,[†] Roman Szostak,[§] and Michal Szostak*[,‡]

[†]*Department of Applied Chemistry, College of Science, China Agricultural University, Beijing 100193, China*

[‡]*Department of Chemistry, Rutgers University, 73 Warren Street, Newark, New Jersey 07102, United States*

[§]*Department of Chemistry, Wroclaw University, F. Joliot-Curie 14, Wroclaw 50-383, Poland*

E-mail: *michal.szostak@rutgers.edu*

Table of Contents 1

List of Known Compounds/General Methods 2
Experimental Procedures and Characterization Data 3
- General Procedures 3
- Characterization Data of Starting Materials 5
- Cross-Coupling of Amides: Variation of Boronic Acid 9
- Cross-Coupling of Amides: Variation of Amides and Esters 15
- Mechanistic Experiments 28
- Computational Methods 31

References 34
^{1}H and ^{13}C NMR Spectra 35

Corresponding Author:
Prof. Dr. M. Szostak
Department of Chemistry, Rutgers University
73 Warren Street, Newark, NJ 07102, United States
E-mail: michal.szostak@rutgers.edu

List of Known Compounds/General Methods

All starting materials reported in the manuscript have been previously described in literature and prepared by the method reported previously. Amides and esters were prepared by standard methods.[1-4] All experiments were performed using standard Schlenk techniques under nitrogen or argon unless stated otherwise. All solvents were purchased at the highest commercial grade and used as received or after purification by passing through activated alumina columns or distillation from sodium/benzophenone under nitrogen. All solvents were deoxygenated prior to use. All other chemicals were purchased at the highest commercial grade and used as received. Reaction glassware was oven-dried at 140 °C for at least 24 h or flame-dried prior to use, allowed to cool under vacuum and purged with argon (three cycles). All products were identified using ^{1}H NMR analysis and comparison with authentic samples. GC and/or GC/MS analysis was used for volatile products. All yields refer to yields determined by ^{1}H NMR and/or GC or GC/MS using an internal standard (optimization) and isolated yields (preparative runs) unless stated otherwise. ^{1}H NMR and ^{13}C NMR spectra were recorded in $CDCl_3$ on Bruker spectrometers at 500 (^{1}H NMR) and 125 MHz (^{13}C NMR). All shifts are reported in parts per million (ppm) relative to residual $CHCl_3$ peak (7.26 and 77.2 ppm, ^{1}H NMR and ^{13}C NMR, respectively). All coupling constants (J) are reported in hertz (Hz). Abbreviations are: s, singlet; d, doublet; t, triplet; q, quartet; brs, broad singlet. GC-MS chromatography was performed using Agilent HP6890 GC System and Agilent 5973A inert XL EI/CI MSD using helium as the carrier gas at a flow rate of 1 mL/min and an initial oven temperature of 50 °C. The injector temperature was 250 °C. The detector temperature was 250 °C. For runs with the initial oven temperature of 50 °C, temperature was increased with a 10 °C/min ramp after 50 °C hold for 3 min to a final temperature of 220 °C, then hold at 220 °C for 15 min (splitless mode of injection, total run time of 22.0 min). High-resolution mass spectra (HRMS) were measured on a 7T Bruker Daltonics FT-MS instrument. All flash chromatography was performed using silica gel, 60 Å, 300 mesh. TLC analysis was carried out on glass plates coated with silica gel 60 F254, 0.2 mm thickness. The plates were visualized using a 254 nm ultraviolet lamp or aqueous potassium permanganate solutions. ^{1}H NMR and ^{13}C NMR data are given for all compounds in the SI for characterization purposes. ^{1}H NMR, ^{13}C NMR, MS and HRMS data are given for all new compounds. All products have been previously reported, unless stated otherwise.

Experimental Procedures and Characterization Data

General Procedure for the Synthesis of Starting Materials. All amides used in this study have been prepared by methods described by us,[1] Garg,[2] and Zou.[3] All esters have been prepared by standard methods.[4] ^{1}H NMR and ^{13}C NMR data for all amides and esters are given in the section below for characterization purposes.

General Procedure for the Suzuki-Miyaura Cross-Coupling of Amides. An oven-dried vial equipped with a stir bar was charged with an amide substrate (neat, 1.0 equiv), potassium carbonate (typically, 4.5 equiv), boronic acid (typically, 3.0 equiv), (**1**) (η^3-1-*t*-Bu-indenyl)Pd(IPr)(Cl))[5] (typically, 3 mol%), placed under a positive pressure of argon, and subjected to three evacuation/backfilling cycles under high vacuum. THF (typically, 0.25 M) was added with vigorous stirring at room temperature and the reaction mixture was stirred for the indicated time. After the indicated time, the reaction mixture was diluted with CH_2Cl_2 (10 mL), filtered, and concentrated. The sample was analyzed by ^{1}H NMR ($CDCl_3$, 500 MHz) and GC-MS to obtain conversion, selectivity and yield using internal standard and comparison with authentic samples. Purification by chromatography on silica gel (EtOAc/hexanes) afforded the title product.

General Procedure for the Suzuki-Miyaura Cross-Coupling of Esters. An oven-dried vial equipped with a stir bar was charged with an ester substrate (neat, 1.0 equiv), potassium carbonate (typically, 7.2 equiv), boronic acid (typically, 4.5 equiv), (**1**) (η^3-1-*t*-Bu-indenyl)Pd(IPr)(Cl)) (typically, 3 mol%), placed under a positive pressure of argon, and subjected to three evacuation/backfilling cycles under high vacuum. THF (typically, 0.25 M) was added with vigorous stirring at room temperature and the reaction mixture was stirred for the indicated time. After the indicated time, the reaction mixture was diluted with CH_2Cl_2 (10 mL), filtered, and concentrated. The sample was analyzed by ^{1}H NMR ($CDCl_3$, 500 MHz) and GC-MS to obtain conversion, selectivity and yield using internal standard and comparison with authentic samples. Purification by chromatography on silica gel (EtOAc/hexanes) afforded the title product.

Representative Cross-Coupling Procedure. An oven-dried vial equipped with a stir bar was charged with phenyl benzoate (1.0 mmol, 198.2 mg, 1.0 equiv), potassium carbonate (4.5 mmol, 621.9 mg, 4.5 equiv), (4-methoxyphenyl)boronic acid (3.0 mmol, 455.9 mg, 3.0 equiv), (**1**) (η^3-1-*t*-Bu-indenyl)Pd(IPr)(Cl)) (1 mol%, 6.9 mg), placed under a positive pressure of argon, and subjected to three evacuation/backfilling cycles under high vacuum. THF (0.25 M) was added with vigorous stirring at room temperature and the reaction mixture was stirred for 15 h at room temperature. After the indicated time, the reaction mixture was diluted with CH_2Cl_2 (20 mL), filtered, and concentrated. The sample was analyzed by ^{1}H NMR ($CDCl_3$, 500 MHz) and GC-MS to obtain conversion, selectivity and yield using internal standard and comparison with authentic samples. Purification by chromatography on silica gel (EtOAc/hexanes) afforded the title product (176.1 mg). Yield 83%. White solid. Characterization data are included in the section below.

Characterization Data for Starting Materials

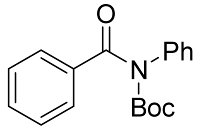

***tert*-Butyl benzoyl(phenyl)carbamate (2a).** White solid. ^{1}H NMR (500 MHz, $CDCl_3$) δ 7.76 (d, *J* = 7.4 Hz, 2 H), 7.55 (t, *J* = 7.3 Hz, 1 H), 7.46 (dd, *J* = 14.1, 7.0 Hz, 4 H), 7.37 (t, *J* = 7.2 Hz, 1 H), 7.30 (d, *J* = 7.8 Hz, 2 H), 1.26 (s, 9 H). ^{13}C NMR (125 MHz, $CDCl_3$) δ 172.8, 153.3, 139.1, 137.0, 131.7, 129.2, 128.3, 128.2, 128.0, 127.8, 83.5, 27.5.

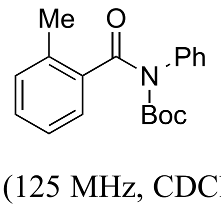

***tert*-Butyl (2-methylbenzoyl)(phenyl)carbamate (2b).** White solid. ^{1}H NMR (500 MHz, $CDCl_3$) δ 7.48 (dd, *J* = 14.3, 7.2 Hz, 3 H), 7.41-7.35 (m, 2 H), 7.32 (d, *J* = 7.9 Hz, 2 H), 7.29-7.25 (m, 2 H), 2.52 (s, 3 H), 1.19 (s, 9 H). ^{13}C NMR (125 MHz, $CDCl_3$) δ 172.6, 152.9, 138.6, 137.9, 135.7, 130.7, 129.9, 129.2, 128.1, 128.0, 126.4, 125.5, 83.6, 27.4, 19.5.

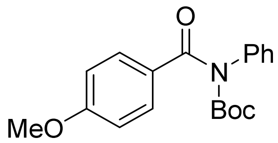

***tert*-Butyl (4-methoxybenzoyl)(phenyl)carbamate (2c).** White solid. ^{1}H NMR (500 MHz, $CDCl_3$) δ 7.77 (d, *J* = 7.6 Hz, 2 H), 7.43 (t, *J* = 7.2 Hz, 2 H), 7.33 (t, *J* = 7.3 Hz, 1 H), 7.28 (d, *J* = 7.9 Hz, 2 H), 6.95 (d, *J* = 7.7 Hz, 2 H), 3.88 (s, 3 H), 1.32 (s, 9 H). ^{13}C NMR (125 MHz, $CDCl_3$) δ 172.1, 162.8, 153.6, 139.5, 130.9, 129.1, 128.7, 127.8, 127.5, 113.6, 83.1, 55.5, 27.7.

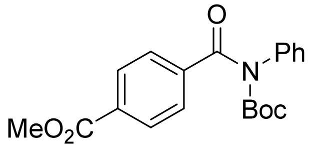

***tert*-Butyl (4-(methoxycarbonyl)benzoyl)(phenyl)carbamate (2d).** White solid. ^{1}H NMR (500 MHz, $CDCl_3$) δ 8.14 (d, *J* = 8.1 Hz, 2 H), 7.78 (d, *J* = 8.1 Hz, 2 H), 7.46 (t, *J* = 7.6 Hz, 2 H), 7.38 (t, *J* = 7.3 Hz, 1 H), 7.28 (d, *J* = 7.8 Hz, 2 H), 3.97 (s, 3 H), 1.26 (s, 9 H). ^{13}C NMR (125 MHz, $CDCl_3$) δ 171.8, 166.2, 153.0, 141.0, 138.6, 132.5, 129.5, 129.3, 128.1, 128.0, 127.8, 84.0, 52.4, 27.5.

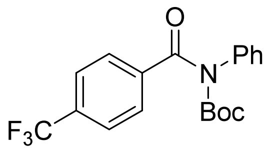

***tert*-Butyl phenyl(4-(trifluoromethyl)benzoyl)carbamate (2e).** White solid. ^{1}H NMR (500 MHz, $CDCl_3$) δ 7.84 (d, *J* = 8.0 Hz, 2 H), 7.74 (d, *J* = 8.2 Hz, 2 H), 7.51-7.44 (m, 2 H), 7.42-7.38 (m, 1 H), 7.31-7.27 (m, 2 H), 1.27 (s, 9 H). ^{13}C NMR (125 MHz, $CDCl_3$) δ 171.4, 152.9, 140.4, 138.5, 133.1 (J^2 = 65.0 Hz),

129.3, 128.2, 128.0, 125.3 (J^3 = 7.5 Hz), 123.7 (J^1 = 270.0 Hz), 84.1, 27.5. ^{19}F NMR (471 MHz, $CDCl_3$) δ -62.92.

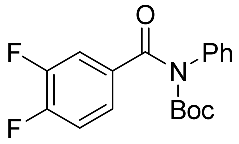

***tert*-Butyl (3,4-difluorobenzoyl)(phenyl)carbamate (2f).** White solid. ^{1}H NMR (500 MHz, $CDCl_3$) δ 7.59 (t, *J* = 8.8 Hz, 1 H), 7.52 (s, 1 H), 7.45 (t, *J* = 7.3 Hz, 2 H), 7.38 (t, *J* = 7.3 Hz, 1 H), 7.24 (t, *J* = 7.8 Hz, 3 H), 1.33 (s, 9 H). ^{13}C NMR (125 MHz, $CDCl_3$) δ 170.3, 152.6 (J^1 = 253.8 Hz), 153.0, 150.07 (J^1 = 250.0 Hz), 138.7, 133.5 (J^3 = 5.0 Hz), 129.3, 128.1, 127.8, 124.9 (J^2 = 15.0 Hz, J^3 = 7.5 Hz), 117.8 (J^2 = 18.8 Hz), 117.3 (J^2 = 17.5 Hz), 84.0, 27.6. ^{19}F NMR (471 MHz, $CDCl_3$) δ -131.6, -136.6.

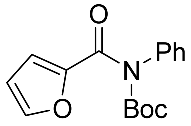

***tert*-Butyl (furan-2-carbonyl)(phenyl)carbamate (2g)**. White solid. ^{1}H NMR (500 MHz, $CDCl_3$) δ 7.58-7.53 (m, 1 H), 7.43 (dd, *J* = 10.4, 4.8 Hz, 2 H), 7.38-7.33 (m, 1 H), 7.30-7.26 (m, 2 H), 7.04 (dd, *J* = 3.5, 0.6 Hz, 1 H), 6.53 (dd, *J* = 3.5, 1.7 Hz, 1 H), 1.42 (s, 9 H). ^{13}C NMR (125 MHz, $CDCl_3$) δ 161.3, 152.9, 148.5, 145.1, 138.7, 129.2, 128.0, 127.9, 118.1, 112.3, 83.3, 27.7.

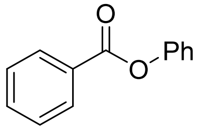

Phenyl benzoate (5a). White solid. ^{1}H NMR (500 MHz, $CDCl_3$) δ 8.29-8.20 (m, 2 H), 7.67 (t, *J* = 7.4 Hz, 1 H), 7.55 (t, *J* = 7.7 Hz, 2 H), 7.47 (t, *J* = 7.9 Hz, 2 H), 7.31 (t, *J* = 7.4 Hz, 1 H), 7.25 (d, *J* = 7.6 Hz, 2 H). ^{13}C NMR (125 MHz, $CDCl_3$) δ 165.2, 151.0, 133.6, 130.2, 129.6, 129.5, 128.6, 125.9, 121.7.

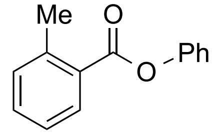

Phenyl 2-methylbenzoate (5b). Oil. ^{1}H NMR (500 MHz, $CDCl_3$) δ 8.20 (d, *J* = 7.7 Hz, 1 H), 7.51 (t, *J* = 7.5 Hz, 1 H), 7.47 (t, *J* = 7.3 Hz, 2 H), 7.36 (t, *J* = 8.0 Hz, 2 H), 7.32-7.28 (m, 1 H), 7.25 (d, *J* = 7.9 Hz, 2 H), 2.71 (s, 3 H). ^{13}C NMR (125 MHz, $CDCl_3$) δ 165.9, 151.0, 141.3, 132.7, 132.0, 131.2, 129.5, 128.6, 125.9, 125.8, 121.9, 22.0.

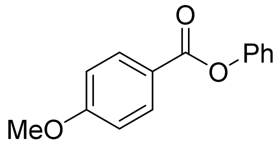

Phenyl 4-methoxybenzoate (5c). White solid. ^{1}H NMR (500 MHz, $CDCl_3$) δ 8.19 (d, *J* = 8.9 Hz, 2 H), 7.45 (t, *J* = 7.9 Hz, 2 H), 7.31-7.27 (m, 1 H), 7.24 (d, *J* = 7.9 Hz, 2 H), 7.02 (d, *J* = 8.8 Hz, 2 H), 3.92 (s, 3

H). ^{13}C NMR (125 MHz, $CDCl_3$) δ 164.9, 163.9, 151.1, 132.3, 129.5, 125.7, 121.9, 121.8, 113.9, 55.5.

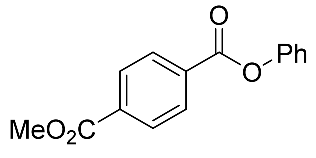

Methyl phenyl terephthalate (5d). White solid. ^{1}H NMR (500 MHz, $CDCl_3$) δ 8.30 (d, J = 7.5 Hz, 2 H), 8.20 (d, J = 7.9 Hz, 2 H), 7.47 (t, J = 7.3 Hz, 2 H), 7.32 (t, J = 7.4 Hz, 1 H), 7.26 (d, J = 8.0 Hz, 2 H), 4.00 (s, 3 H). ^{13}C NMR (125 MHz, $CDCl_3$) δ 166.2, 164.4, 150.8, 134.5, 133.4, 130.2, 129.7, 129.6, 126.1, 121.6, 52.5.

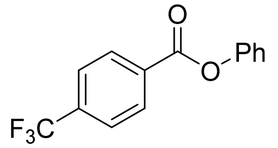

Phenyl 4-(trifluoromethyl)benzoate (5e). White solid. ^{1}H NMR (500 MHz, $CDCl_3$) δ 8.35 (d, J = 8.1 Hz, 2 H), 7.81 (d, J = 8.2 Hz, 2 H), 7.48 (t, J = 7.9 Hz, 2 H), 7.33 (t, J = 7.5 Hz, 1 H), 7.27-7.23 (m, 2 H). ^{13}C NMR (125 MHz, $CDCl_3$) δ 164.0, 150.7, 135.1 (J^2 = 32.8 Hz), 132.9, 130.6, 129.6, 126.3, 125.6 (J^4 = 3.7 Hz), 123.6 (J^1 = 272.8 Hz), 121.6. ^{19}F NMR (471 MHz, $CDCl_3$) δ -63.2.

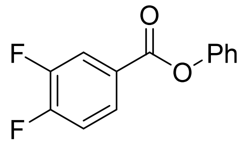

Phenyl 3,4-difluorobenzoate (5f). White solid. ^{1}H NMR (500 MHz, $CDCl_3$) δ 8.10-7.96 (m, 2 H), 7.47 (t, J = 7.3 Hz, 2 H), 7.38-7.29 (m, 2 H), 7.23 (d, J = 7.9 Hz, 2 H). ^{13}C NMR (125 MHz, $CDCl_3$) δ 163.3, 154.1 (J^1 = 257.2 Hz), 150.7, 150.2 (J^1 = 250.9 Hz), 129.6, 127.2 (J^3 = 7.5 Hz, J^4 = 3.6 Hz), 126.6 (J^3 = 7.7 Hz), 126.2, 121.5, 119.5 (J^2 = 18.8 Hz), 117.6 (J^2 = 17.9 Hz). ^{19}F NMR (471 MHz, $CDCl_3$) δ -128.9, -136.0.

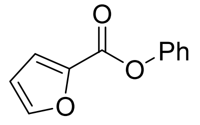

Phenyl furan-2-carboxylate (5g). Oil. ^{1}H NMR (500 MHz, $CDCl_3$) δ 7.70 (s, 1 H), 7.45 (t, J = 7.4 Hz, 2 H), 7.41 (d, J = 2.3 Hz, 1 H), 7.32-7.28 (m, 1 H), 7.24 (d, J = 7.9 Hz, 2 H), 6.62 (d, J = 1.4 Hz, 1 H). ^{13}C NMR (125 MHz, $CDCl_3$) δ 156.9, 150.2, 147.1, 144.1, 129.5, 126.1, 121.6, 119.4, 112.2.

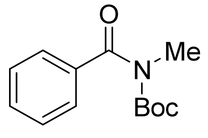

***tert*-Butyl (benzoyl)(methyl)carbamate (2h)**. Oil. ^{1}H NMR (500 MHz, $CDCl_3$) δ 7.53 (d, J = 7.2 Hz, 2 H), 7.48 (t, J = 7.4 Hz, 1 H), 7.40 (t, J = 7.5 Hz, 2 H), 3.33 (s, 3 H), 1.17 (s, 9 H). ^{13}C NMR (125 MHz, $CDCl_3$) δ 173.6, 153.6, 137.9, 130.9, 128.0, 127.4, 83.0, 32.6, 27.4.

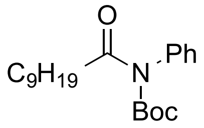

***tert*-Butyl decanoylphenylcarbamate (2i)**. Oil. ^{1}H NMR (500 MHz, $CDCl_3$) δ 7.41 (t, *J* = 7.2 Hz, 2 H), 7.34 (t, *J* = 7.3 Hz, 1 H), 7.09 (d, *J* = 7.7 Hz, 2 H), 2.92 (t, *J* = 7.4 Hz, 2 H), 1.70 (p, *J* = 7.3, 6.8 Hz, 2 H), 1.40 (s, 9 H), 1.29 (s, 12 H), 0.90 (t, *J* = 6.4 Hz, 3 H). ^{13}C NMR (125 MHz, $CDCl_3$) δ 176.0, 152.3, 139.2, 128.9, 128.2, 127.7, 82.9, 38.0, 31.9, 29.5, 29.3, 29.2, 27.8, 25.0, 22.7, 14.1.

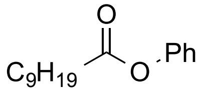

Phenyl decanoate (5h). Oil. ^{1}H NMR (500 MHz, $CDCl_3$) δ 7.40 (t, *J* = 7.8 Hz, 2 H), 7.25 (t, *J* = 7.4 Hz, 1 H), 7.10 (d, *J* = 7.7 Hz, 2 H), 2.58 (t, *J* = 7.5 Hz, 2 H), 1.85-1.73 (m, 2 H), 1.50-1.41 (m, 2 H), 1.40-1.25 (m, 10 H), 0.92 (t, *J* = 6.7 Hz, 3 H). ^{13}C NMR (125 MHz, $CDCl_3$) δ 172.4, 150.8, 129.4, 125.7, 121.6, 34.4, 31.9, 29.5, 29.3, 29.1, 25.0, 22.7, 14.1.

Cross-Coupling of Amides and Esters: Variation of Boronic Acid

Benzophenone (4a) (Table 1, Entry 1)[1d]

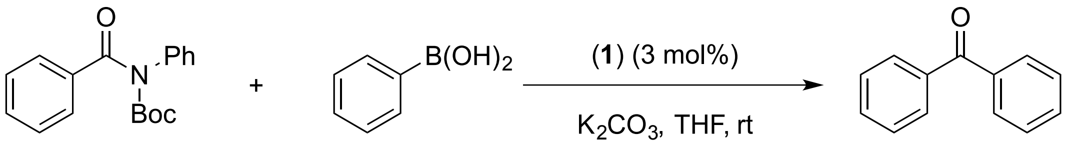

2a **3a** **4a**

According to the general procedure, the reaction of *tert*-butyl benzoyl(phenyl)carbamate (0.20 mmol), phenylboronic acid (3.0 equiv), K_2CO_3 (4.5 equiv), (**1**) (η^3-1-*t*-Bu-indenyl)Pd(IPr)(Cl) (3 mol%) in THF (0.25 M) for 15 h at room temperature, afforded after filtration and chromatography the title compound in 98% yield (35.6 mg). White solid. ^{1}H NMR (500 MHz, $CDCl_3$) δ 7.83-7.78 (m, 4 H), 7.59 (t, *J* = 7.4 Hz, 2 H), 7.48 (t, *J* = 7.7 Hz, 4 H). ^{13}C NMR (125 MHz, $CDCl_3$) δ 196.8, 137.6, 132.4, 130.1, 128.3.

Phenyl(*o*-tolyl)methanone (4b) (Table 1, Entry 2)[1d]

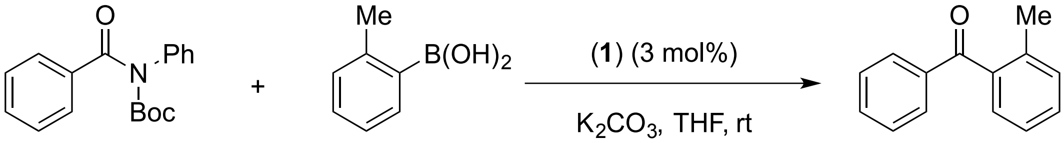

2a **3b** **4b**

According to the general procedure, the reaction of *tert*-butyl benzoyl(phenyl)carbamate (0.20 mmol), *o*-tolyboronic acid (3.0 equiv), K_2CO_3 (4.5 equiv), (**1**) (η^3-1-*t*-Bu-indenyl)Pd(IPr)(Cl) (3 mol%) in THF (0.25 M) for 15 h at room temperature, afforded after filtration and chromatography the title compound in 95% yield (37.3 mg). Oil. ^{1}H NMR (500 MHz, $CDCl_3$) δ 7.80 (d, *J* = 7.5 Hz, 2 H), 7.58 (t, *J* = 7.4 Hz, 1 H), 7.46 (t, *J* = 7.7 Hz, 2 H), 7.39 (t, *J* = 7.5 Hz, 1 H), 7.30 (dd, *J* = 11.0, 7.6 Hz, 2 H), 7.26-7.22 (m, 1 H), 2.33 (s, 3 H). ^{13}C NMR (125 MHz, $CDCl_3$) δ 198.7, 138.6, 137.8, 136.8, 133.1, 131.0, 130.3, 130.2, 128.5, 128.5, 125.2, 20.0.

Phenyl(*p*-tolyl)methanone (4c) (Table 1, Entry 3)[1d]

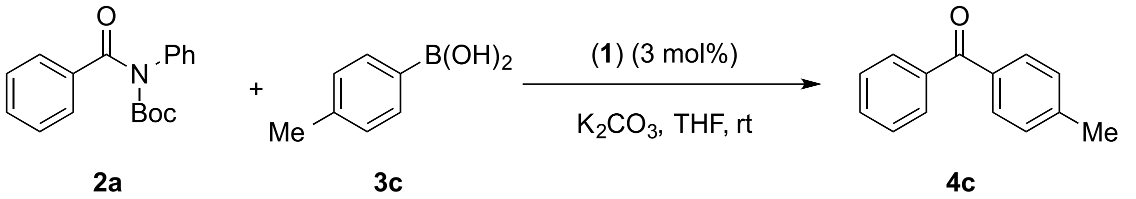

According to the general procedure, the reaction of *tert*-butyl benzoyl(phenyl)carbamate (0.20 mmol), *p*-tolylboronic acid (3.0 equiv), K_2CO_3 (4.5 equiv), (**1**) (η^3-1-*t*-Bu-indenyl)Pd(IPr)(Cl) (3 mol%) in THF (0.25 M) for 15 h at room temperature, afforded after filtration and chromatography the title compound in 96% yield (37.5 mg). White solid. ^{1}H NMR (500 MHz, $CDCl_3$) δ 7.79 (d, J = 7.2 Hz, 2 H), 7.73 (d, J = 8.1 Hz, 2 H), 7.57 (t, J = 7.4 Hz, 1 H), 7.47 (t, J = 7.6 Hz, 2 H), 7.28 (d, J = 7.9 Hz, 2 H), 2.44 (s, 3 H). ^{13}C NMR (125 MHz, $CDCl_3$) δ 196.5, 143.3, 138.0, 134.9, 132.2, 130.3, 130.0, 129.0, 128.2, 21.7.

(4-Methoxyphenyl)(phenyl)methanone (4d) (Table 1, Entry 4)[1d]

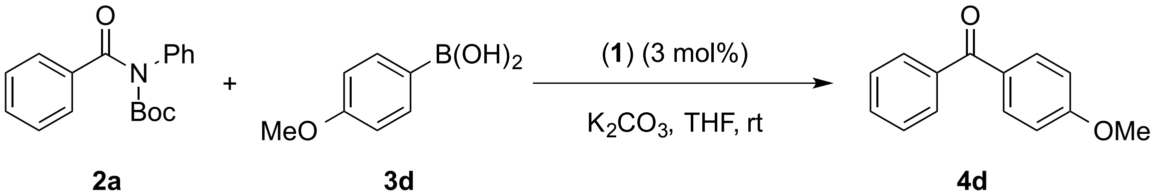

According to the general procedure, the reaction of *tert*-butyl benzoyl(phenyl)carbamate (0.20 mmol), (4-methoxyphenyl)boronic acid (3.0 equiv), K_2CO_3 (4.5 equiv), (**1**) (η^3-1-*t*-Bu-indenyl)Pd(IPr)(Cl) (3 mol%) in THF (0.25 M) for 15 h at room temperature, afforded after filtration and chromatography the title compound in 98% yield (41.7 mg). White solid. ^{1}H NMR (500 MHz, $CDCl_3$) δ 7.83 (d, J = 8.8 Hz, 2 H), 7.76 (d, J = 7.4 Hz, 2 H), 7.57 (t, J = 7.4 Hz, 1 H), 7.47 (t, J = 7.6 Hz, 2 H), 6.97 (d, J = 8.8 Hz, 2 H), 3.89 (s, 3 H). ^{13}C NMR (125 MHz, $CDCl_3$) δ 195.6, 163.2, 138.3, 132.6, 131.9, 130.2, 129.8, 128.2, 113.6, 55.5.

Methyl 4-benzoylbenzoate (4e) (Table 1, Entry 5)[1d]

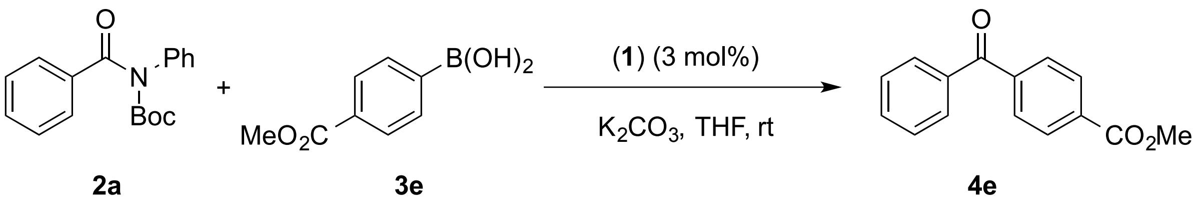

According to the general procedure, the reaction of *tert*-butyl benzoyl(phenyl)carbamate (0.20 mmol), (4-(methoxycarbonyl)phenyl)boronic acid (3.0 equiv), K_2CO_3 (4.5 equiv), (**1**) (η^3-1-*t*-Bu-indenyl)Pd(IPr)(Cl) (3 mol%) for 15 h at room temperature, afforded after filtration and chromatography the title compound in 95% yield (45.6 mg). White solid. ^{1}H NMR (500 MHz, $CDCl_3$) δ 8.15 (d, *J* = 8.3 Hz, 2 H), 7.84 (d, *J* = 8.3 Hz, 2 H), 7.80 (d, *J* = 7.3 Hz, 2 H), 7.62 (t, *J* = 7.4 Hz, 1 H), 7.50 (t, *J* = 7.7 Hz, 2 H), 3.96 (s, 3 H). ^{13}C NMR (125 MHz, $CDCl_3$) δ 196.1, 166.3, 141.3, 137.0, 133.2, 133.0, 130.1, 129.8, 129.5, 128.5, 52.5.

Phenyl(4-(trifluoromethyl)phenyl)methanone (4f) (Table 1, Entry 6)[1d]

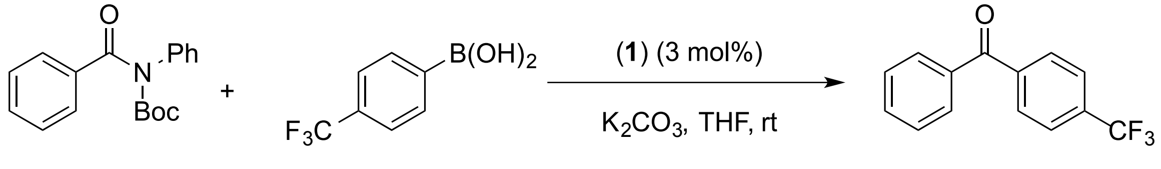

2a **3f** **4f**

According to the general procedure, the reaction of *tert*-butyl benzoyl(phenyl)carbamate (0.20 mmol), 4-(trifluoromethyl)phenylboronic acid (3.0 equiv), K_2CO_3 (4.5 equiv), (**1**) (η^3-1-*t*-Bu-indenyl)Pd(IPr)(Cl) (3 mol%) in THF (0.25 M) for 15 h at room temperature, afforded after filtration and chromatography the title compound in 98% yield (49.1 mg). White solid. ^{1}H NMR (500 MHz, $CDCl_3$) δ 7.90 (d, *J* = 8.1 Hz, 2 H), 7.81 (d, *J* = 7.3 Hz, 2 H), 7.76 (d, *J* = 8.1 Hz, 2 H), 7.63 (t, *J* = 7.4 Hz, 1 H), 7.51 (t, *J* = 7.7 Hz, 2 H). ^{13}C NMR (125 MHz, $CDCl_3$) δ 195.6, 140.7, 136.8, 133.7 (J^2 = 32.6 Hz), 133.1, 130.1 (J^4 = 4.2 Hz), 128.6, 125.4 (J^4 = 3.7 Hz), 123.7 (J^1 = 272.7 Hz). ^{19}F NMR (471 MHz, $CDCl_3$) δ -63.0.

Benzophenone (4a) (Table 2, Entry 1)[1d]

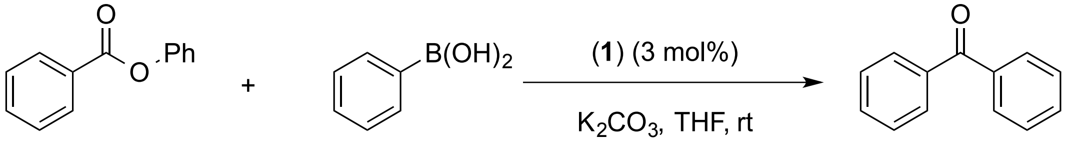

5a **3a** **4a**

According to the general procedure, the reaction of phenyl benzoate (0.20 mmol), phenylboronic acid (4.5 equiv), K_2CO_3 (7.2 equiv), (**1**) (η^3-1-*t*-Bu-indenyl)Pd(IPr)(Cl) (3 mol%) in THF (0.25 M) for 15 h at room temperature, afforded after filtration and chromatography the title compound in 83% yield (30.2 mg). White solid. ^{1}H NMR (500 MHz, $CDCl_3$) δ 7.83-7.78 (m, 4 H), 7.59 (t, *J*

= 7.4 Hz, 2 H), 7.48 (t, *J* = 7.7 Hz, 4 H). ^{13}C NMR (125 MHz, $CDCl_3$) δ 196.8, 137.6, 132.4, 130.1, 128.3.

Phenyl(*o*-tolyl)methanone (4b) (Table 2, Entry 2)[1d]

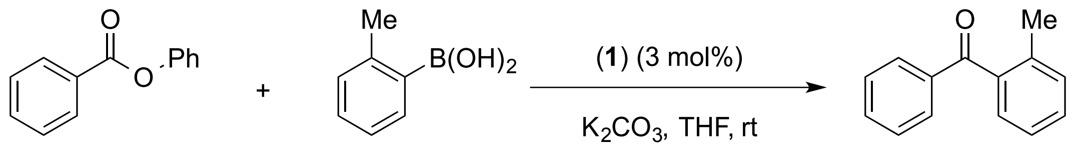

5a **3b** **4b**

According to the general procedure, the reaction of phenyl benzoate (0.20 mmol), *o*-tolyboronic acid (4.5 equiv), K_2CO_3 (7.2 equiv), (**1**) (η^3-1-*t*-Bu-indenyl)Pd(IPr)(Cl) (3 mol%) in THF (0.25 M) for 15 h at room temperature, afforded after filtration and chromatography the title compound in 72% yield (28.4 mg). Oil. ^{1}H NMR (500 MHz, $CDCl_3$) δ 7.80 (d, *J* = 7.5 Hz, 2 H), 7.58 (t, *J* = 7.4 Hz, 1 H), 7.46 (t, *J* = 7.7 Hz, 2 H), 7.39 (t, *J* = 7.5 Hz, 1 H), 7.30 (dd, *J* = 11.0, 7.6 Hz, 2 H), 7.26-7.22 (m, 1 H), 2.33 (s, 3 H). ^{13}C NMR (125 MHz, $CDCl_3$) δ 198.7, 138.6, 137.8, 136.8, 133.1, 131.0, 130.3, 130.2, 128.5, 128.5, 125.2, 20.0.

Phenyl(*p*-tolyl)methanone (4c) (Table 2, Entry 3)[1d]

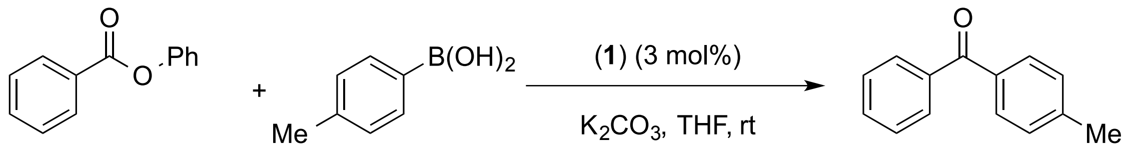

5a **3c** **4c**

According to the general procedure, the reaction of phenyl benzoate (0.20 mmol), *p*-tolylboronic acid (4.5 equiv), K_2CO_3 (7.2 equiv), (**1**) (η^3-1-*t*-Bu-indenyl)Pd(IPr)(Cl) (3 mol%) in THF (0.25 M) for 15 h at room temperature, afforded after filtration and chromatography the title compound in 80% yield (31.3 mg). White solid. ^{1}H NMR (500 MHz, $CDCl_3$) δ 7.79 (d, *J* = 7.2 Hz, 2 H), 7.73 (d, *J* = 8.1 Hz, 2 H), 7.57 (t, *J* = 7.4 Hz, 1 H), 7.47 (t, *J* = 7.6 Hz, 2 H), 7.28 (d, *J* = 7.9 Hz, 2 H), 2.44 (s, 3 H). ^{13}C NMR (125 MHz, $CDCl_3$) δ 196.5, 143.3, 138.0, 134.9, 132.2, 130.3, 130.0, 129.0, 128.2, 21.7.

(4-Methoxyphenyl)(phenyl)methanone (4d) (Table 2, Entry 4)[1d]

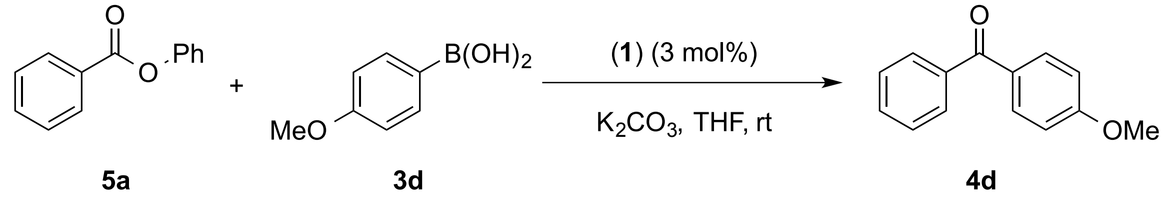

According to the general procedure, the reaction of phenyl benzoate (0.20 mmol), (4-methoxyphenyl)boronic acid (4.5 equiv), K_2CO_3 (7.2 equiv), (**1**) (η^3-1-*t*-Bu-indenyl)Pd(IPr)(Cl) (3 mol%) in THF (0.25 M) for 15 h at room temperature, afforded after filtration and chromatography the title compound in 98% yield (41.6 mg). White solid. ^{1}H NMR (500 MHz, $CDCl_3$) δ 7.83 (d, J = 8.8 Hz, 2 H), 7.76 (d, J = 7.4 Hz, 2 H), 7.57 (t, J = 7.4 Hz, 1 H), 7.47 (t, J = 7.6 Hz, 2 H), 6.97 (d, J = 8.8 Hz, 2 H), 3.89 (s, 3 H). ^{13}C NMR (125 MHz, $CDCl_3$) δ 195.6, 163.2, 138.3, 132.6, 131.9, 130.2, 129.8, 128.2, 113.6, 55.5.

Methyl 4-benzoylbenzoate (4e) (Table 2, Entry 5)[1d]

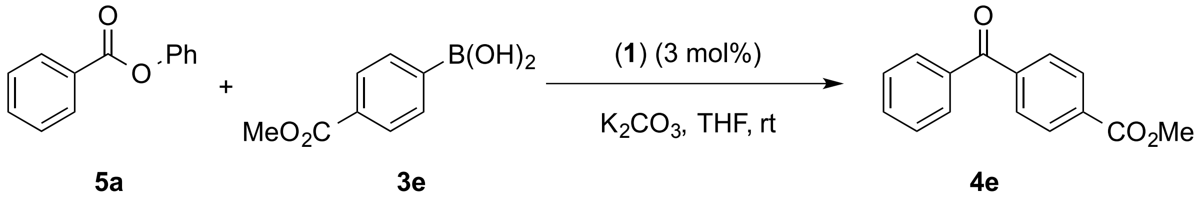

According to the general procedure, the reaction of phenyl benzoate (0.20 mmol), (4-(methoxycarbonyl)phenyl)boronic acid (4.5 equiv), K_2CO_3 (7.2 equiv), (**1**) (η^3-1-*t*-Bu-indenyl)Pd(IPr)(Cl) (3 mol%) in THF (0.25 M) for 15 h at room temperature, afforded after filtration chromatography the title compound in 62% yield (29.9 mg). White solid. ^{1}H NMR (500 MHz, $CDCl_3$) δ 8.15 (d, J = 8.3 Hz, 2 H), 7.84 (d, J = 8.3 Hz, 2 H), 7.80 (d, J = 7.3 Hz, 2 H), 7.62 (t, J = 7.4 Hz, 1 H), 7.50 (t, J = 7.7 Hz, 2 H), 3.96 (s, 3 H). ^{13}C NMR (125 MHz, $CDCl_3$) δ 196.1, 166.3, 141.3, 137.0, 133.2, 133.0, 130.1, 129.8, 129.5, 128.5, 52.5.

Phenyl(4-(trifluoromethyl)phenyl)methanone (4f) (Table 2, Entry 6)[1d]

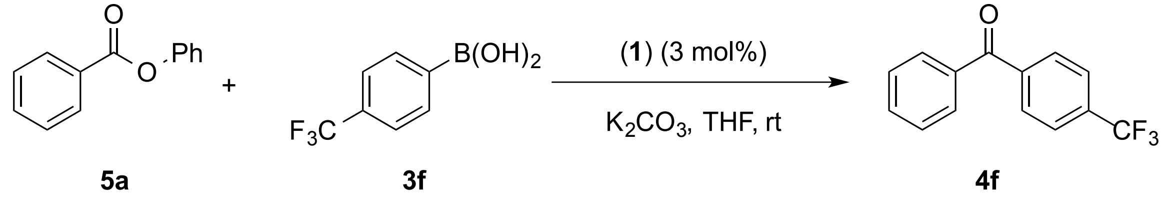

According to the general procedure, the reaction of phenyl benzoate (0.20 mmol), 4-(trifluoromethyl)phenylboronic acid (4.5 equiv), K_2CO_3 (7.2 equiv), (**1**) (η^3-1-*t*-Bu-indenyl)Pd(IPr)(Cl) (3 mol%) in THF (0.25 M) for 15 h at room temperature, afforded after filtration and chromatography the title compound in 86% yield (43.2 mg). White solid. ^{1}H NMR (500 MHz, $CDCl_3$) δ 7.90 (d, J = 8.1 Hz, 2 H), 7.81 (d, J = 7.3 Hz, 2 H), 7.76 (d, J = 8.1 Hz, 2 H), 7.63 (t, J = 7.4 Hz, 1 H), 7.51 (t, J = 7.7 Hz, 2 H). ^{13}C NMR (125 MHz, $CDCl_3$) δ 195.6, 140.7, 136.8, 133.7 (J^2 = 32.6 Hz), 133.1, 130.1 (J^4 = 4.2 Hz), 128.6, 125.4 (J^4 = 3.7 Hz), 123.7 (J^1 = 272.7 Hz). ^{19}F NMR (471 MHz, $CDCl_3$) δ -63.0.

Cross-Coupling of Amides and Esters: Variation of Amides and Esters

Phenyl(*o*-tolyl)methanone (4b) (Table 1, Entry 7)[1d]

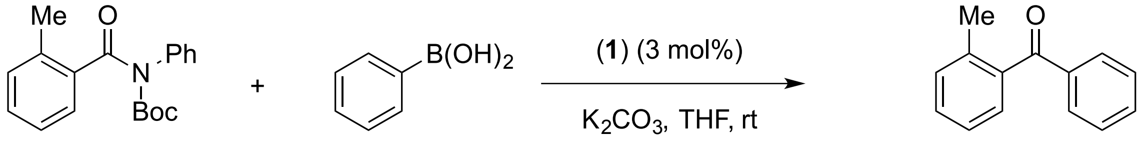

2b **3a** **4b**

According to the general procedure, the reaction of *tert*-butyl (2-methylbenzoyl)(phenyl)carbamate (0.20 mmol), phenylboronic acid (3.0 equiv), K_2CO_3 (4.5 equiv), (**1**) (η^3-1-*t*-Bu-indenyl)Pd(IPr)(Cl) (3 mol%) in THF (0.25 M) for 15 h at room temperature, afforded after filtration and chromatography the title compound in 78% yield (30.7 mg). Oil. ^{1}H NMR (500 MHz, $CDCl_3$) δ 7.80 (d, *J* = 7.5 Hz, 2 H), 7.58 (t, *J* = 7.4 Hz, 1 H), 7.46 (t, *J* = 7.7 Hz, 2 H), 7.39 (t, *J* = 7.5 Hz, 1 H), 7.30 (dd, *J* = 11.0, 7.6 Hz, 2 H), 7.26-7.22 (m, 1 H), 2.33 (s, 3 H). ^{13}C NMR (125 MHz, $CDCl_3$) δ 198.7, 138.6, 137.8, 136.8, 133.1, 131.0, 130.3, 130.2, 128.5, 128.5, 125.2, 20.0.

(4-Methoxyphenyl)(phenyl)methanone (4d) (Table 1, Entry 8)[1d]

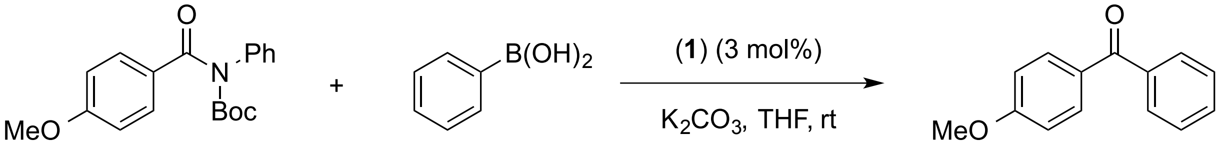

2c **3a** **4d**

According to the general procedure, the reaction of *tert*-butyl (4-methoxybenzoyl)(phenyl)carbamate (0.20 mmol), phenylboronic acid (3.0 equiv), K_2CO_3 (4.5 equiv), (**1**) (η^3-1-*t*-Bu-indenyl)Pd(IPr)(Cl) (3 mol%) in THF (0.25 M) for 15 h at room temperature, afforded after filtration and chromatography the title compound in 98% yield (41.4 mg). White solid. ^{1}H NMR (500 MHz, $CDCl_3$) δ 7.83 (d, *J* = 8.8 Hz, 2 H), 7.76 (d, *J* = 7.4 Hz, 2 H), 7.57 (t, *J* = 7.4 Hz, 1 H), 7.47 (t, *J* = 7.6 Hz, 2 H), 6.97 (d, *J* = 8.8 Hz, 2 H), 3.89 (s, 3 H). ^{13}C NMR (125 MHz, $CDCl_3$) δ 195.6, 163.2, 138.3, 132.6, 131.9, 130.2, 129.8, 128.2, 113.6, 55.5.

Methyl 4-benzoylbenzoate (4e) (Table 1, Entry 9)[1d]

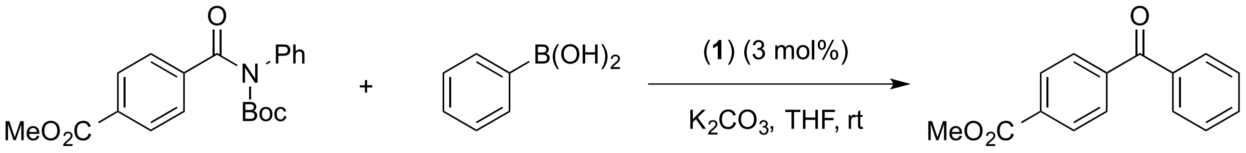

2d **3a** **4e**

According to the general procedure, the reaction of *tert*-butyl (4-(methoxycarbonyl)benzoyl)(phenyl)carbamate (0.20 mmol), phenylboronic acid (3.0 equiv), K_2CO_3 (4.5 equiv), (**1**) (η^3-1-*t*-Bu-indenyl)Pd(IPr)(Cl) (3 mol%) in THF (0.25 M) for 15 h at room temperature, afforded after filtration and chromatography the title compound in 80% yield (38.5 mg). White solid. ^{1}H NMR (500 MHz, $CDCl_3$) δ 8.15 (d, *J* = 8.3 Hz, 2 H), 7.84 (d, *J* = 8.3 Hz, 2 H), 7.80 (d, *J* = 7.3 Hz, 2 H), 7.62 (t, *J* = 7.4 Hz, 1 H), 7.50 (t, *J* = 7.7 Hz, 2 H), 3.96 (s, 3 H). ^{13}C NMR (125 MHz, $CDCl_3$) δ 196.1, 166.3, 141.3, 137.0, 133.2, 133.0, 130.1, 129.8, 129.5, 128.5, 52.5.

Phenyl(4-(trifluoromethyl)phenyl)methanone (4f) (Table 1, Entry 10)[1d]

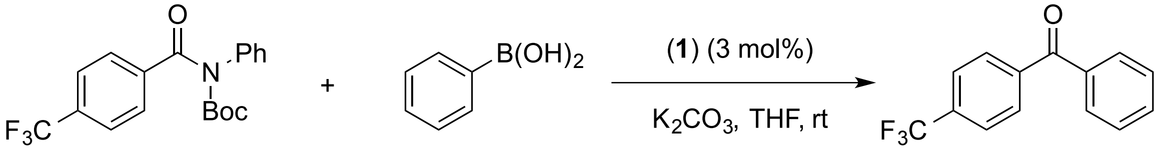

2e **3a** **4f**

According to the general procedure, the reaction of *tert*-butyl phenyl(4-(trifluoromethyl)benzoyl)carbamate (0.20 mmol), phenylboronic acid (3.0 equiv), K_2CO_3 (4.5 equiv), (**1**) (η^3-1-*t*-Bu-indenyl)Pd(IPr)(Cl) (3 mol%) in THF (0.25 M) for 15 h at room temperature, afforded after filtration and chromatography the title compound in 98% yield (48.8 mg). White solid. ^{1}H NMR (500 MHz, $CDCl_3$) δ 7.90 (d, *J* = 8.1 Hz, 2 H), 7.81 (d, *J* = 7.3 Hz, 2 H), 7.76 (d, *J* = 8.1 Hz, 2 H), 7.63 (t, *J* = 7.4 Hz, 1 H), 7.51 (t, *J* = 7.7 Hz, 2 H). ^{13}C NMR (125 MHz, $CDCl_3$) δ 195.6, 140.7, 136.8, 133.7 (J^2 = 32.6 Hz), 133.1, 130.1 (J^4 = 4.2 Hz), 128.6, 125.4 (J^4 = 3.7 Hz), 123.7 (J^1 = 272.7 Hz). ^{19}F NMR (471 MHz, $CDCl_3$) δ -63.0.

(3,4-Difluorophenyl)(phenyl)methanone (4g) (Table 1, Entry 11)[9]

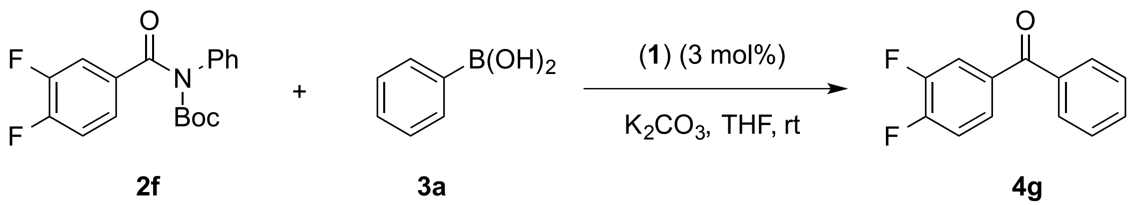

According to the general procedure, the reaction of *tert*-butyl (3,4-difluorobenzoyl)(phenyl)carbamate (0.20 mmol), phenylboronic acid (3.0 equiv), K_2CO_3 (4.5 equiv), (**1**) (η^3-1-*t*-Bu-indenyl)Pd(IPr)(Cl) (3 mol%) in THF (0.25 M) for 15 h at room temperature, afforded after filtration and chromatography the title compound in 95% yield (41.4 mg). White solid. ^{1}H NMR (500 MHz, $CDCl_3$) δ 7.77 (d, *J* = 7.4 Hz, 2 H), 7.70-7.66 (m, 1 H), 7.64-7.57 (m, 2 H), 7.50 (t, *J* = 7.7 Hz, 2 H), 7.30-7.24 (m, 1 H). ^{13}C NMR (125 MHz, $CDCl_3$) δ 194.1, 153.3 (J^1 = 256.3 Hz, J^3 = 12.9 Hz), 150.2 (J^1 = 251.1 Hz, J^3 = 12.9 Hz), 136.9, 134.5 (J^3 = 8.0 Hz), 132.8, 129.9, 128.5, 127.1 (J^3 = 7.3 Hz, J^4 = 3.6 Hz), 119.3 (J^2 = 18.1 Hz), 117.3 (J^2 = 17.8 Hz). ^{19}F NMR (471 MHz, $CDCl_3$) δ -130.6, -136.2.

Furan-2-yl(phenyl)methanone (4h) (Table 1, Entry 12)[4b]

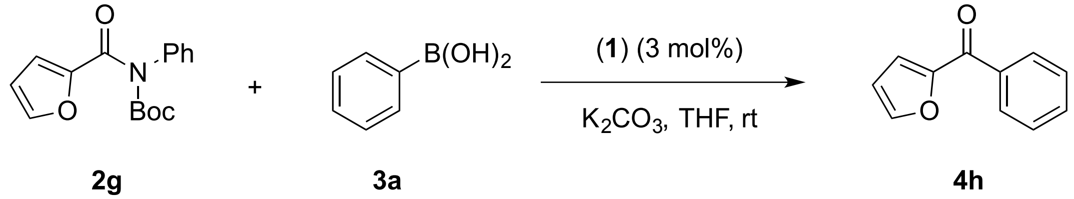

According to the general procedure, the reaction of *tert*-butyl (furan-2-carbonyl)(phenyl)carbamate (0.20 mmol), phenylboronic acid (3.0 equiv), K_2CO_3 (4.5 equiv), (**1**) (η^3-1-*t*-Bu-indenyl)Pd(IPr)(Cl) (3 mol%) in THF (0.25 M) for 15 h at room temperature, afforded after filtration and chromatography the title compound in 91% yield (31.3 mg). Oil. ^{1}H NMR (500 MHz, $CDCl_3$) δ 7.98 (d, *J* = 7.3 Hz, 2 H), 7.71 (s, 1 H), 7.60 (t, *J* = 7.4 Hz, 1 H), 7.50 (t, *J* = 7.6 Hz, 2 H), 7.24 (d, *J* = 3.5 Hz, 1 H), 6.61-6.59 (m, 1 H). ^{13}C NMR (125 MHz, $CDCl_3$) δ 182.6, 152.4, 147.1, 137.3, 132.6, 129.3, 128.4, 120.6, 112.2.

Phenyl(*o*-tolyl)methanone (4b) (Table 2, Entry 7)[1d]

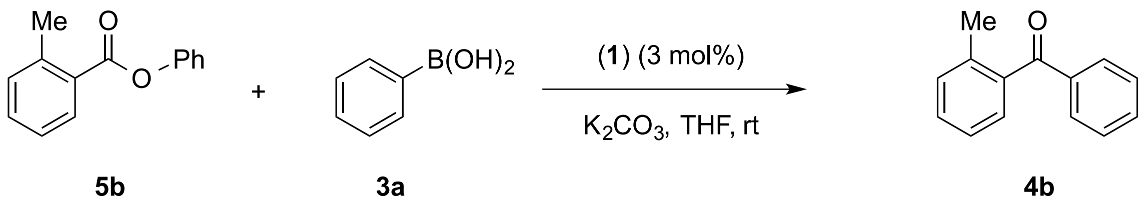

According to the general procedure, the reaction of phenyl 2-methylbenzoate (0.20 mmol), phenylboronic acid (4.5 equiv), K_2CO_3 (7.2 equiv), (**1**) (η^3-1-*t*-Bu-indenyl)Pd(IPr)(Cl) (3 mol%) in THF (0.25 M) for 15 h at room temperature, afforded after filtration and chromatography the title compound in 75% yield (29.6 mg). Oil. ^{1}H NMR (500 MHz, $CDCl_3$) δ 7.80 (d, *J* = 7.5 Hz, 2 H), 7.58 (t, *J* = 7.4 Hz, 1 H), 7.46 (t, *J* = 7.7 Hz, 2 H), 7.39 (t, *J* = 7.5 Hz, 1 H), 7.30 (dd, *J* = 11.0, 7.6 Hz, 2 H), 7.26-7.22 (m, 1 H), 2.33 (s, 3 H). ^{13}C NMR (125 MHz, $CDCl_3$) δ 198.7, 138.6, 137.8, 136.8, 133.1, 131.0, 130.3, 130.2, 128.5, 128.5, 125.2, 20.0.

(4-Methoxyphenyl)(phenyl)methanone (4d) (Table 2, Entry 8)[1d]

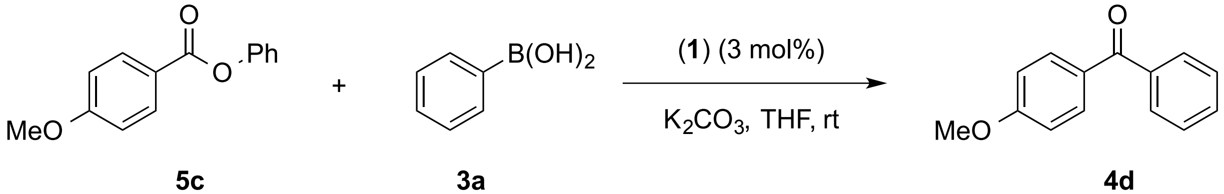

According to the general procedure, the reaction of phenyl 4-methoxybenzoate (0.20 mmol), phenylboronic acid (4.5 equiv), K_2CO_3 (7.2 equiv), (**1**) (η^3-1-*t*-Bu-indenyl)Pd(IPr)(Cl) (3 mol%) in THF (0.25 M) for 15 h at room temperature, afforded after filtration and chromatography the title compound in 75% yield (31.9 mg). White solid. ^{1}H NMR (500 MHz, $CDCl_3$) δ 7.83 (d, *J* = 8.8 Hz, 2 H), 7.76 (d, *J* = 7.4 Hz, 2 H), 7.57 (t, *J* = 7.4 Hz, 1 H), 7.47 (t, *J* = 7.6 Hz, 2 H), 6.97 (d, *J* = 8.8 Hz, 2 H), 3.89 (s, 3 H). ^{13}C NMR (125 MHz, $CDCl_3$) δ 195.6, 163.2, 138.3, 132.6, 131.9, 130.2, 129.8, 128.2, 113.6, 55.5.

Methyl 4-benzoylbenzoate (4e) (Table 2, Entry 9)[1d]

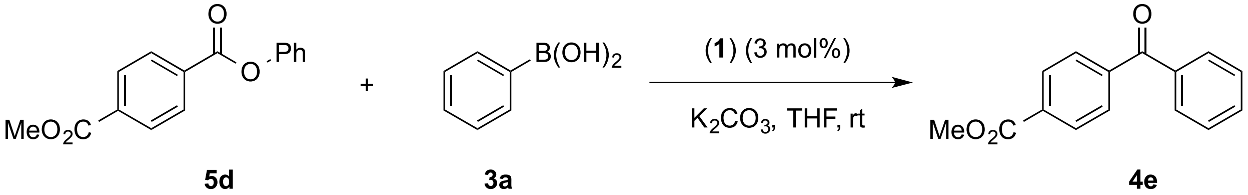

According to the general procedure, the reaction of methyl phenyl terephthalate (0.20 mmol), phenylboronic acid (4.5 equiv), K_2CO_3 (7.2 equiv), (**1**) (η^3-1-*t*-Bu-indenyl)Pd(IPr)(Cl) (3 mol%) in THF (0.25 M) for 15 h at room temperature, afforded after filtration and chromatography the title compound in 91% yield (43.7 mg). White solid. ^{1}H NMR (500 MHz, $CDCl_3$) δ 8.15 (d, J = 8.3 Hz, 2 H), 7.84 (d, J = 8.3 Hz, 2 H), 7.80 (d, J = 7.3 Hz, 2 H), 7.62 (t, J = 7.4 Hz, 1 H), 7.50 (t, J = 7.7 Hz, 2 H), 3.96 (s, 3 H). ^{13}C NMR (125 MHz, $CDCl_3$) δ 196.1, 166.3, 141.3, 137.0, 133.2, 133.0, 130.1, 129.8, 129.5, 128.5, 52.5.

Phenyl(4-(trifluoromethyl)phenyl)methanone (4f) (Table 2, Entry 10)[1d]

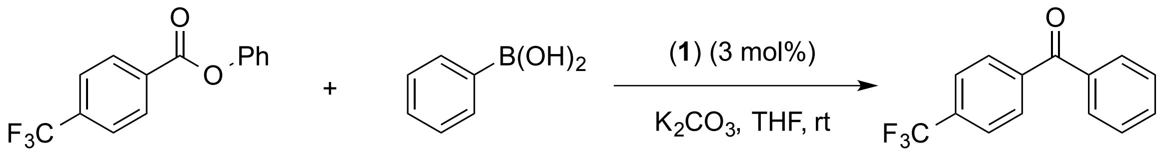

5e **3a** **4f**

According to the general procedure, the reaction of phenyl 4-(trifluoromethyl)benzoate (0.20 mmol), phenylboronic acid (4.5 equiv), K_2CO_3 (7.2 equiv), (**1**) (η^3-1-*t*-Bu-indenyl)Pd(IPr)(Cl) (3 mol%) in THF (0.25 M) for 15 h at room temperature, afforded after filtration and chromatography the title compound in 97% yield (48.3 mg). White solid. ^{1}H NMR (500 MHz, $CDCl_3$) δ 7.90 (d, J = 8.1 Hz, 2 H), 7.81 (d, J = 7.3 Hz, 2 H), 7.76 (d, J = 8.1 Hz, 2 H), 7.63 (t, J = 7.4 Hz, 1 H), 7.51 (t, J = 7.7 Hz, 2 H). ^{13}C NMR (125 MHz, $CDCl_3$) δ 195.6, 140.7, 136.8, 133.7 (J^2 = 32.6 Hz), 133.1, 130.1 (J^4 = 4.2 Hz), 128.6, 125.4 (J^4 = 3.7 Hz), 123.7 (J^1 = 272.7 Hz). ^{19}F NMR (471 MHz, $CDCl_3$) δ -63.0.

(3,4-Difluorophenyl)(phenyl)methanone (4g) (Table 2, Entry 11)[9]

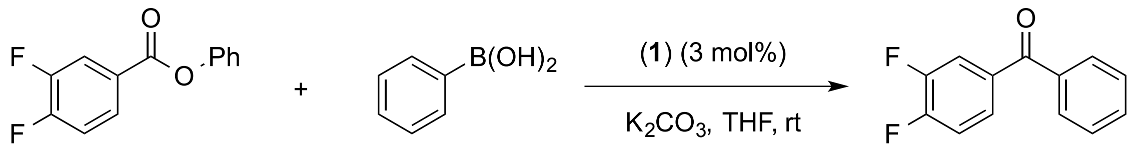

5f **3a** **4g**

According to the general procedure, the reaction of phenyl 3,4-difluorobenzoate (0.20 mmol), phenylboronic acid (4.5 equiv), K_2CO_3 (7.2 equiv), (**1**) (η^3-1-*t*-Bu-indenyl)Pd(IPr)(Cl) (3 mol%) in THF (0.25 M) for 15 h at room temperature, afforded after filtration and chromatography the title compound in 96% yield (41.8 mg). White solid. ^{1}H NMR (500 MHz, $CDCl_3$) δ 7.77 (d, J =

7.4 Hz, 2 H), 7.70-7.66 (m, 1 H), 7.64-7.57 (m, 2 H), 7.50 (t, J = 7.7 Hz, 2 H), 7.30-7.24 (m, 1 H). ^{13}C NMR (125 MHz, $CDCl_3$) δ 194.1, 153.3 (J^1 = 256.3 Hz, J^3 = 12.9 Hz), 150.2 (J^1 = 251.1 Hz, J^3 = 12.9 Hz), 136.9, 134.5 (J^3 = 8.0 Hz), 132.8, 129.9, 128.5, 127.1 (J^3 = 7.3 Hz, J^4 = 3.6 Hz), 119.3 (J^2 = 18.1 Hz), 117.4 (J^2 = 17.8 Hz). ^{19}F NMR (471 MHz, $CDCl_3$) δ -130.6, -136.2.

Furan-2-yl(phenyl)methanone (4h) (Table 2, Entry 12)[4b]

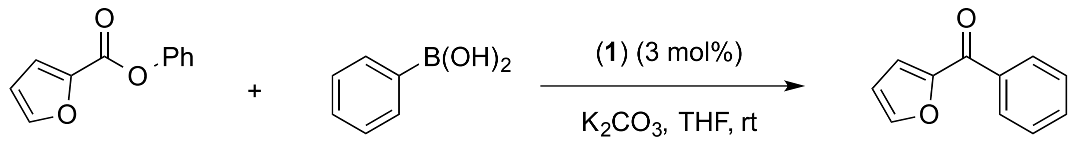

5g **3a** **4h**

According to the general procedure, the reaction of phenyl furan-2-carboxylate (0.20 mmol), phenylboronic acid (4.5 equiv), K_2CO_3 (7.2 equiv), (**1**) (η^3-1-*t*-Bu-indenyl)Pd(IPr)(Cl) (3 mol%) in THF (0.25 M) for 15 h at room temperature, afforded after filtration and chromatography the title compound in 60% yield (20.8 mg). Oil. ^{1}H NMR (500 MHz, $CDCl_3$) δ 7.98 (d, J = 7.3 Hz, 2 H), 7.71 (s, 1 H), 7.60 (t, J = 7.4 Hz, 1 H), 7.50 (t, J = 7.6 Hz, 2 H), 7.24 (d, J = 3.5 Hz, 1 H), 6.61-6.59 (m, 1 H). ^{13}C NMR (125 MHz, $CDCl_3$) δ 182.6, 152.4, 147.1, 137.3, 132.6, 129.3, 128.4, 120.6, 112.2.

(4-Methoxyphenyl)(phenyl)methanone (4d) (Eq. 2, Amide = *N*-Me/Boc)[1d]

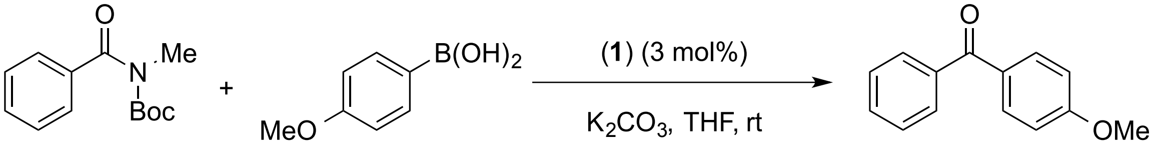

2h **3d** **4d**

According to the general procedure, the reaction of *tert*-butyl (benzoyl)(methyl)carbamate (0.20 mmol), (4-methoxyphenyl)boronic acid (3.0 equiv), K_2CO_3 (4.5 equiv), (**1**) (η^3-1-*t*-Bu-indenyl)Pd(IPr)(Cl) (3 mol%) in THF (0.25 M) for 15 h at room temperature, afforded after filtration and chromatography the title compound in 92% yield (39.2 mg). White solid. ^{1}H NMR (500 MHz, $CDCl_3$) δ 7.83 (d, J = 8.8 Hz, 2 H), 7.76 (d, J = 7.4 Hz, 2 H), 7.57 (t, J = 7.4 Hz, 1 H), 7.47 (t, J = 7.6 Hz, 2 H), 6.97 (d, J = 8.8 Hz, 2 H), 3.89 (s, 3 H). ^{13}C NMR (125 MHz, $CDCl_3$) δ 195.6, 163.2, 138.3, 132.6, 131.9, 130.2, 129.8, 128.2, 113.6, 55.5.

One-Pot *N*-Activation/ Cross-Coupling

<u>General Procedure.</u> An oven-dried vial equipped with a stir bar was charged with *N*-phenylbenzamide (neat, 0.20 mmol, 1.0 equiv), di-*tert*-butyl-dicarbonate (1.0 equiv), and DMAP (10 mol%). Acetonitrile (0.25 M) was added with vigorous stirring and the resulting reaction mixture was stirred at room temperature for 15 h. After the indicated time, the reaction mixture was concentrated under high vacuum, 4-methylphenylboronic acid (3.0 equiv), potassium carbonate (4.5 equiv), (**1**) (η^3-1-*t*-Bu-indenyl)Pd(IPr)(Cl) (3 mol%) were added, the reaction vial was placed under a positive pressure of argon, and subjected to three evacuation/backfilling cycles under high vacuum. THF (0.25 M) was added with vigorous stirring and the resulting reaction mixture was stirred at room temperature for 15 h. After the indicated time, the reaction mixture was diluted with CH_2Cl_2 (10 mL), filtered, and concentrated. The sample was analyzed by ^{1}H NMR (CDCl3, 500 MHz) and/or GC-MS to obtain conversion, selectivity and yield using internal standard and comparison with authentic samples. Purification by chromatography on silica gel (EtOAc/hexanes) afforded the tile product. Yield 94% (36.8 mg). White solid. Characterization data matched those described above.

Scheme SI-1. One-Pot *N*-Activation/Suzuki-Miyaura Cross-Coupling at Room Temperature Catalyzed by (**1**).

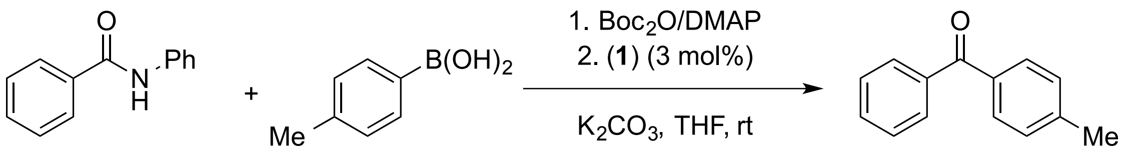

6 **3c** **4c**

Conditions: (**1**) (3 mol%), 4-Tol-B(OH)$_2$ (3.0 equiv), K_2CO_3 (4.5 equiv), THF (0.25 M), 23 °C, 15 h.

Cross-Coupling of Amides and Esters: Additional Examples

Bis(4-methoxyphenyl)methanone (4i) (Table 3, Entry 1)[4b]

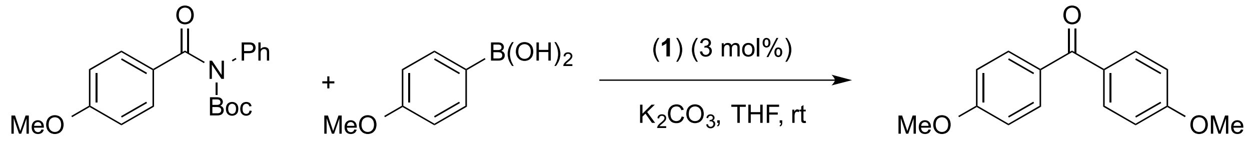

According to the general procedure, the reaction of *tert*-butyl (4-methoxybenzoyl)(phenyl)carbamate (0.20 mmol), (4-methoxyphenyl)boronic acid (3.0 equiv), K_2CO_3 (4.5 equiv), (**1**) (η^3-1-*t*-Bu-indenyl)Pd(IPr)(Cl) (3 mol%) in THF (0.25 M) for 15 h at room temperature, afforded after filtration and chromatography the title compound in 98% yield (47.5 mg). White solid. ^{1}H NMR (500 MHz, $CDCl_3$) δ 7.81 (d, *J* = 8.3 Hz, 4 H), 6.99 (d, *J* = 8.3 Hz, 4 H), 3.91 (s, 6 H). ^{13}C NMR (125 MHz, $CDCl_3$) δ 194.5, 162.9, 132.2, 130.8, 113.5, 55.5.

(4-Methoxyphenyl)(4-(trifluoromethyl)phenyl)methanone (4j) (Table 3, Entry 2)[4b]

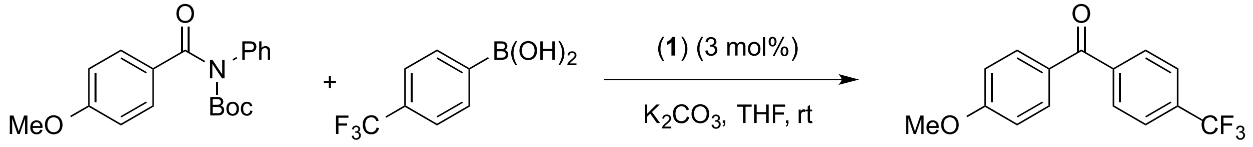

According to the general procedure, the reaction of *tert*-butyl (4-methoxybenzoyl)(phenyl)carbamate (0.20 mmol), 4-(trifluoromethyl)phenylboronic acid (3.0 equiv), K_2CO_3 (4.5 equiv), (**1**) (η^3-1-*t*-Bu-indenyl)Pd(IPr)(Cl) (3 mol%) in THF (0.25 M) for 15 h at room temperature, afforded after filtration and chromatography the title compound in 95% yield (53.3 mg). White solid. ^{1}H NMR (500 MHz, $CDCl_3$) δ 7.86 (t, *J* = 9.0 Hz, 4H), 7.77 (d, *J* = 7.8 Hz, 2H), 7.01 (d, *J* = 8.3 Hz, 2H), 3.93 (s, 3H). ^{13}C NMR (150 MHz, $CDCl_3$) δ 194.3, 163.7, 141.5, 133.4 (t, *J* = 32.7 Hz), 132.7, 129.8, 129.4, 125.3, 123.8 (d, *J* = 272.1 Hz), 113.8, 55.6. ^{19}F NMR (470 MHz, $CDCl_3$) δ -62.9.

(4-Methoxyphenyl)(4-(trifluoromethyl)phenyl)methanone (4j) (Table 3, Entry 3)[4b]

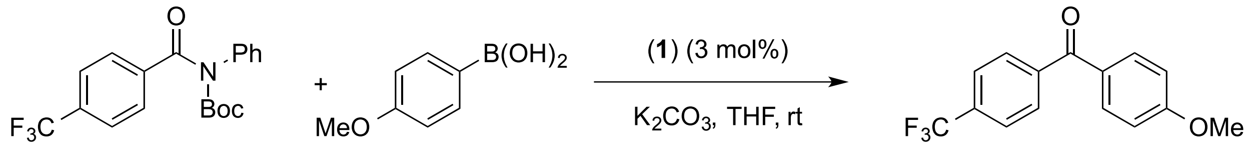

2e 3d 4j

According to the general procedure, the reaction of *tert*-butyl phenyl(4-(trifluoromethyl)benzoyl)carbamate (0.20 mmol), (4-methoxyphenyl)boronic acid (3.0 equiv), K_2CO_3 (4.5 equiv), (**1**) (η^3-1-*t*-Bu-indenyl)Pd(IPr)(Cl) (3 mol%) in THF (0.25 M) for 15 h at room temperature, afforded after filtration and chromatography the title compound in 94% yield (52.5 mg). White solid. ^{1}H NMR (500 MHz, $CDCl_3$) δ 7.86 (t, *J* = 9.0 Hz, 4H), 7.77 (d, *J* = 7.8 Hz, 2H), 7.01 (d, *J* = 8.3 Hz, 2H), 3.93 (s, 3H). ^{13}C NMR (150 MHz, $CDCl_3$) δ 194.3, 163.7, 141.5, 133.4 (t, *J* = 32.7 Hz), 132.7, 129.8, 129.4, 125.3, 123.8 (d, *J* = 272.1 Hz), 113.8, 55.6. ^{19}F NMR (470 MHz, $CDCl_3$) δ -62.9.

Bis(4-(trifluoromethyl)phenyl)methanone (4k) (Table 3, Entry 4)[4b]

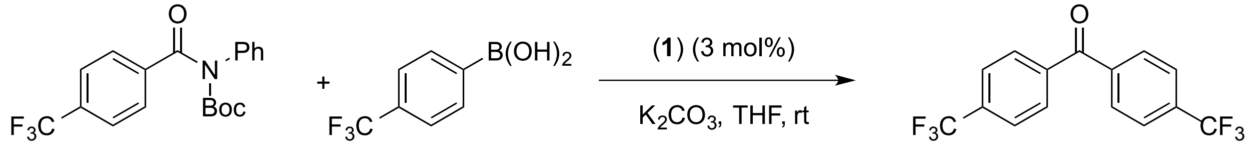

2e 3f 4k

According to the general procedure, the reaction of *tert*-butyl phenyl(4-(trifluoromethyl)benzoyl)carbamate (0.20 mmol), 4-(trifluoromethyl)phenylboronic acid (3.0 equiv), K_2CO_3 (4.5 equiv), (**1**) (η^3-1-*t*-Bu-indenyl)Pd(IPr)(Cl) (3 mol%) in THF (0.25 M) for 15 h at room temperature, afforded after filtration and chromatography the title compound in 96% yield (61.1 mg). White solid. ^{1}H NMR (500 MHz, $CDCl_3$) δ 7.93 (d, *J* = 7.4 Hz, 4 H), 7.81 (d, *J* = 7.6 Hz, 4 H). ^{13}C NMR (125 MHz, $CDCl_3$) δ 194.4, 139.8, 134.4 (d, *J* = 32.8 Hz), 130.2 (s), 125.6, 123.5 (d, *J* = 273.0 Hz). ^{19}F NMR (471 MHz, $CDCl_3$) δ -63.1.

Di-*o*-tolylmethanone (4l) (Table 3, Entry 5)[3]

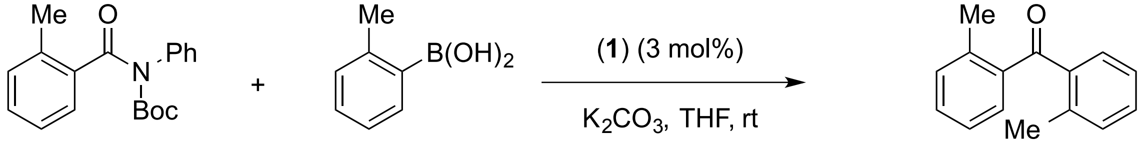

2b 3b 4l

According to the general procedure, the reaction of *tert*-butyl phenyl(2-methyl benzoyl)carbamate (0.20 mmol), *o*-tolyboronic acid (3.0 equiv), K_2CO_3 (4.5 equiv), (**1**) (η^3-1-*t*-Bu-indenyl)Pd(IPr)(Cl) (3 mol%) in THF (0.25 M) for 15 h at room temperature, afforded after filtration and chromatography the title compound in 62% yield (25.9 mg). White solid. ^{1}H NMR (500 MHz, $CDCl_3$) δ 7.42 (t, *J* = 7.4 Hz, 2 H), 7.32 (dt, *J* = 14.6, 7.3 Hz, 4 H), 7.23 (t, *J* = 7.4 Hz, 2 H), 2.48 (s, 6 H). ^{13}C NMR (125 MHz, $CDCl_3$) δ 200.8, 139.0, 138.2, 131.4, 131.1, 130.3, 125.4, 20.7.

1-Phenyldecan-1-one (4m) (Table 3, Entry 6)[1d]

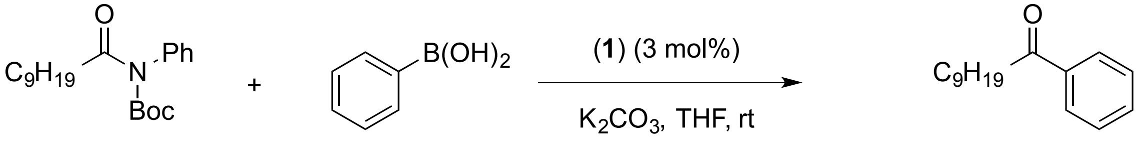

2i **3a** **4m**

According to the general procedure, the reaction of *tert*-butyl decanoylphenylcarbamate (0.20 mmol), phenylboronic acid (3.0 equiv), K_2CO_3 (4.5 equiv), (**1**) (η^3-1-*t*-Bu-indenyl)Pd(IPr)(Cl) (3 mol%) in THF (0.25 M) for 15 h at room temperature, afforded after filtration and chromatography the title compound in 76% yield (35.5 mg). White solid. ^{1}H NMR (500 MHz, $CDCl_3$) δ 7.96 (d, *J* = 7.4 Hz, 2 H), 7.55 (t, *J* = 7.3 Hz, 1 H), 7.46 (t, *J* = 7.6 Hz, 2 H), 2.96 (t, *J* = 7.4 Hz, 2 H), 1.78-1.69 (m, 2 H), 1.40-1.22 (m, 12 H), 0.88 (t, *J* = 6.8 Hz, 3 H). ^{13}C NMR (125 MHz, $CDCl_3$) δ 200.7, 137.1, 132.9, 128.6, 128.1, 38.7, 31.9, 29.5, 29.5, 29.4, 29.3, 24.4, 22.69, 14.1.

Bis(4-methoxyphenyl)methanone (4i) (Table 3, Entry 7)[4b]

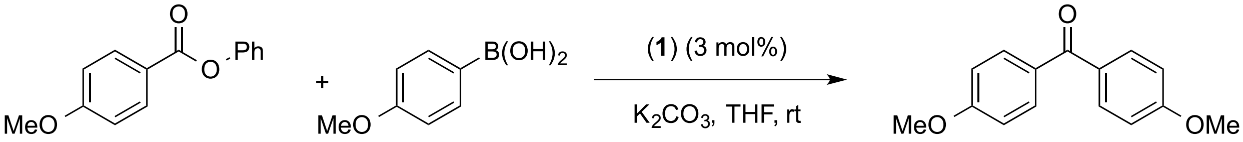

5c **3d** **4i**

According to the general procedure, the reaction of phenyl 4-methoxybenzoate (0.20 mmol), (4-methoxyphenyl)boronic acid (4.5 equiv), K_2CO_3 (7.2 equiv), (**1**) (η^3-1-*t*-Bu-indenyl)Pd(IPr)(Cl) (3 mol%) in THF (0.25 M) for 15 h at room temperature, afforded after filtration and chromatography the title compound in 96% yield (46.5 mg). White solid. ^{1}H NMR (500 MHz,

$CDCl_3$) δ 7.81 (d, *J* = 8.3 Hz, 4 H), 6.99 (d, *J* = 8.3 Hz, 4 H), 3.91 (s, 6 H). ^{13}C NMR (125 MHz, $CDCl_3$) δ 194.5, 162.9, 132.2, 130.8, 113.4, 55.5.

(4-Methoxyphenyl)(4-(trifluoromethyl)phenyl)methanone (4j) (Table 3, Entry 8)[4b]

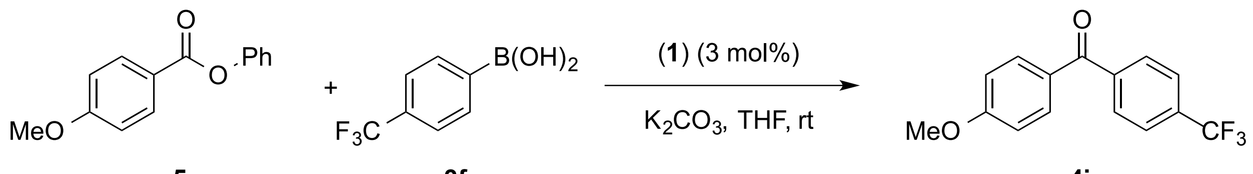

According to the general procedure, the reaction of phenyl 4-(trifluoromethyl)phenylboronic acid (0.20 mmol), (4-methoxyphenyl)boronic acid (4.5 equiv), K_2CO_3 (7.2 equiv), (**1**) (η^3-1-*t*-Bu-indenyl)Pd(IPr)(Cl) (3 mol%) in THF (0.25 M) for 15 h at room temperature, afforded after filtration and chromatography the title compound in 94% yield (52.7 mg). White solid. ^{1}H NMR (500 MHz, $CDCl_3$) δ 7.86 (t, *J* = 9.0 Hz, 4H), 7.77 (d, *J* = 7.8 Hz, 2H), 7.01 (d, *J* = 8.3 Hz, 2H), 3.93 (s, 3H). ^{13}C NMR (150 MHz, $CDCl_3$) δ 194.3, 163.7, 141.5, 133.4 (t, *J* = 32.7 Hz), 132.7, 129.8, 129.4, 125.3, 123.8 (d, *J* = 272.1 Hz), 113.8, 55.6. ^{19}F NMR (470 MHz, $CDCl_3$) δ -62.9.

(4-Methoxyphenyl)(4-(trifluoromethyl)phenyl)methanone (4j) (Table 3, Entry 9)[4b]

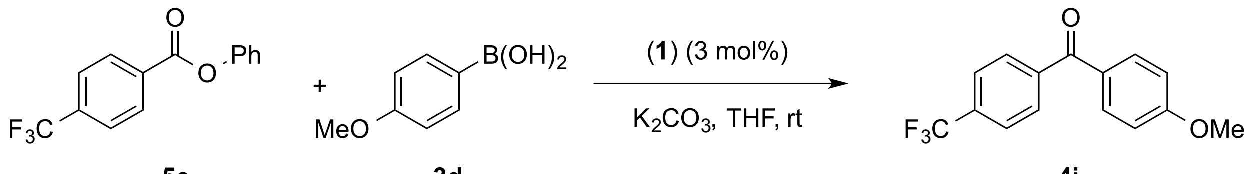

According to the general procedure, the reaction of phenyl 4-(trifluoromethyl)benzoate (0.20 mmol), (4-methoxyphenyl)boronic acid (4.5 equiv), K_2CO_3 (7.2 equiv), (**1**) (η^3-1-*t*-Bu-indenyl)Pd(IPr)(Cl) (3 mol%) in THF (0.25 M) for 15 h at room temperature, afforded after filtration and chromatography the title compound in 98% yield (54.9 mg). White solid. White solid. ^{1}H NMR (500 MHz, $CDCl_3$) δ 7.86 (t, *J* = 9.0 Hz, 4H), 7.77 (d, *J* = 7.8 Hz, 2H), 7.01 (d, *J* = 8.3 Hz, 2H), 3.93 (s, 3H). ^{13}C NMR (150 MHz, $CDCl_3$) δ 194.3, 163.7, 141.5, 133.4 (t, *J* = 32.7 Hz), 132.7, 129.8, 129.4, 125.3, 123.8 (d, *J* = 272.1 Hz), 113.8, 55.6. ^{19}F NMR (470 MHz, $CDCl_3$) δ -62.9.

Bis(4-(trifluoromethyl)phenyl)methanone (4k) (Table 3, Entry 10)[4b]

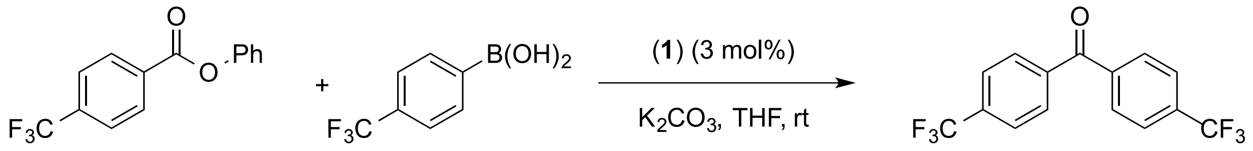

5e 3f 4k

According to the general procedure, the reaction of phenyl 4-(trifluoromethyl)benzoate (0.20 mmol), (4-methoxyphenyl)boronic acid (4.5 equiv), K_2CO_3 (7.2 equiv), (**1**) (η^3-1-*t*-Bu-indenyl)Pd(IPr)(Cl) (3 mol%) in THF (0.25 M) for 15 h at room temperature, afforded after filtration and chromatography the title compound in 93% yield (59.2 mg). White solid. ^{1}H NMR (500 MHz, $CDCl_3$) δ 7.93 (d, *J* = 7.4 Hz, 4 H), 7.81 (d, *J* = 7.6 Hz, 4 H). ^{13}C NMR (125 MHz, $CDCl_3$) δ 194.4, 139.8, 134.4 (d, *J* = 32.8 Hz), 130.2 (s), 125.6, 123.5 (d, *J* = 273.0 Hz). ^{19}F NMR (471 MHz, $CDCl_3$) δ -63.1.

Di-*o*-tolylmethanone (4l) (Table 3, Entry 11)[3]

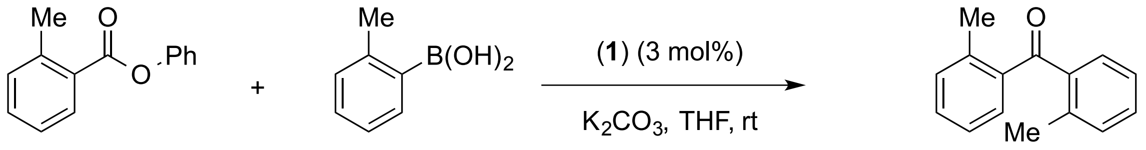

5b 3b 4l

According to the general procedure, the reaction of phenyl 2-methylbenzoate (0.20 mmol), *o*-tolyboronic acid (4.5 equiv), K_2CO_3 (7.2 equiv), (**1**) (η^3-1-*t*-Bu-indenyl)Pd(IPr)(Cl) (3 mol%) in THF (0.25 M) for 15 h at room temperature, afforded after filtration and chromatography the title compound in 49% yield (20.5 mg). White solid. ^{1}H NMR (500 MHz, $CDCl_3$) δ 7.42 (t, *J* = 7.4 Hz, 2 H), 7.32 (dt, *J* = 14.6, 7.3 Hz, 4 H), 7.23 (t, *J* = 7.4 Hz, 2 H), 2.48 (s, 6 H). ^{13}C NMR (125 MHz, $CDCl_3$) δ 200.8, 139.0, 138.2, 131.4, 131.1, 130.3, 125.4, 20.687

1-Phenyldecan-1-one (4m) (Table 3, Entry 12)[1b]

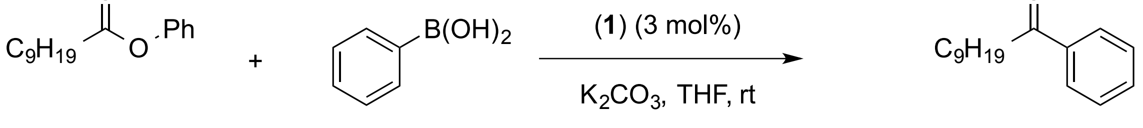

5h 3a 4m

According to the general procedure, the reaction of phenyl decanoate (0.20 mmol), phenylboronic acid (4.5 equiv), K_2CO_3 (7.2 equiv), (**1**) (η^3-1-*t*-Bu-indenyl)Pd(IPr)(Cl) (3 mol%)

in THF (0.25 M) for 15 h at room temperature, afforded after filtration and chromatography the title compound in 91% yield (42.3 mg). White solid. ^{1}H NMR (500 MHz, $CDCl_3$) δ 7.96 (d, *J* = 7.4 Hz, 2 H), 7.55 (t, *J* = 7.3 Hz, 1 H), 7.46 (t, *J* = 7.6 Hz, 2 H), 2.96 (t, *J* = 7.4 Hz, 2 H), 1.78-1.69 (m, 2 H), 1.40-1.22 (m, 12 H), 0.88 (t, *J* = 6.8 Hz, 3 H). ^{13}C NMR (125 MHz, $CDCl_3$) δ 200.7, 137.1, 132.9, 128.6, 128.1, 38.7, 31.9, 29.5, 29.5, 29.4, 29.3, 24.4, 22.7, 14.1.

Determination of Kinetic Profiles

General Procedure. An oven-dried vial equipped with a stir bar was charged with an amide or ester substrate (neat, 0.20 mmol, 1.0 equiv), potassium carbonate (4.5 equiv), boronic acid (3.0 equiv), (**1**) (η^3-1-*t*-Bu-indenyl)Pd(IPr)(Cl)) (3 mol%), placed under a positive pressure of argon, and subjected to three evacuation/backfilling cycles under high vacuum. THF (0.25 M) was added with vigorous stirring and the reaction mixture was stirred at room temperature for the indicated time. After the indicated time, the reaction mixture was diluted with CH_2Cl_2 (10 mL), filtered, and concentrated. The sample was analyzed by ^{1}H NMR ($CDCl_3$, 500 MHz) and/or GC-MS to obtain conversion, selectivity and yield using internal standard and comparison with authentic samples.

Scheme SI-2. Determination of Relative Reaction Rates in the Suzuki-Miyaura Cross-Coupling Catalyzed by (**1**).

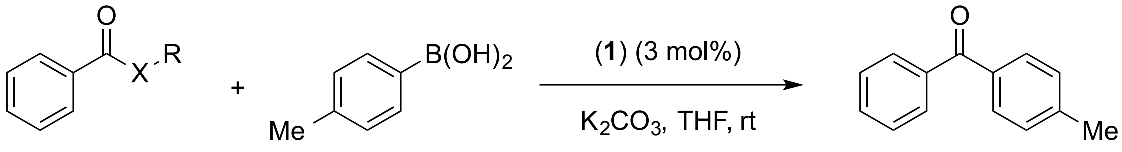

XR = N,N-Boc/Ph (**2a**) **3c** **4c**
XR = OPh (**5a**)
XR = N,N-Ts/Ph (**8**)

Conditions: (**1**) (3 mol%), 4-Tol-B$(OH)_2$ (3.0 equiv), K_2CO_3 (4.5 equiv), THF (0.25 M), 23 °C, 0-300 min.

The relative reactivity of amide and ester electrophiles in the Suzuki-Miyaura cross-coupling at room temperature was studied by determining kinetic profiles.[6] In addition, N,N-Ts/Ph amide (**8**) was included for comparison.[7,1-3] The observed kinetic profiles are consistent with the barrier to isomerization of the acyl bond.[8] Note that these conditions allow for synthetically useful selectivity between **2a**, **5a** and **8**. Further studies on the mechanism of the Suzuki-Miyaura reaction are underway in our laboratory.

Selectivity Studies

General Procedure. An oven-dried vial equipped with a stir bar was charged with two carbonyl substrates (each 0.20 mmol, 1.0 equiv), potassium carbonate (4.5 equiv), 4-methylphenylboronic acid (0.50 equiv), (**1**) (η^3-1-*t*-Bu-indenyl)Pd(IPr)(Cl)) (3 mol%), placed under a positive pressure of argon, and subjected to three evacuation/backfilling cycles under high vacuum. THF (0.25 M) was added with vigorous stirring and the reaction mixture was stirred at room temperature for the indicated time. After the indicated time, the reaction mixture was diluted with CH_2Cl_2 (10 mL), filtered, and concentrated. The sample was analyzed by ^{1}H NMR ($CDCl_3$, 500 MHz) and/or GC-MS to obtain conversion, selectivity and yield using internal standard and comparison with authentic samples.

Table SI-1. Selectivity Study in the Suzuki-Miyaura Cross-Coupling at Room Temperature Catalyzed by (**1**).

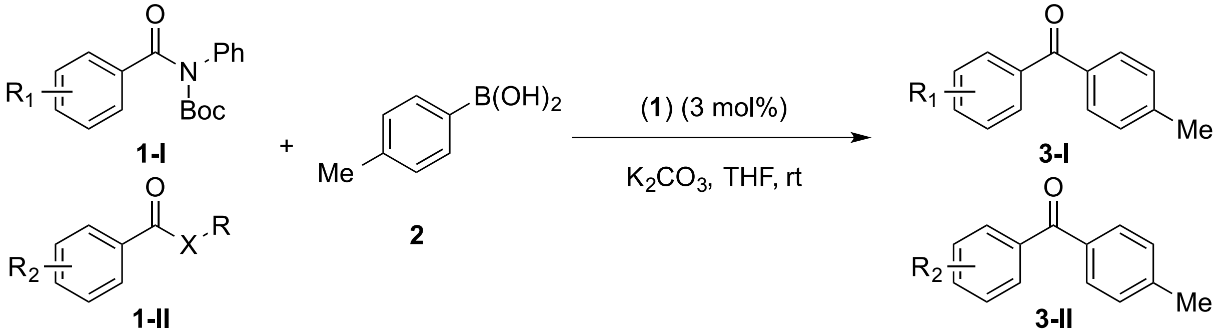

Entry	**1-I** (R_1)	**1-II** (XR, R_2)	Amide/Ester (equiv)	**3-I**:**3-II** (R_1:R_2)[b]
1	4-Me-	N,N-Ph/Me, H	2.0	>98:2
2	4-Me-	N,N-Ph/Ts, H	2.0	>98:2
3	4-Me-	OPh, H	2.0	10:1

[a]Conditions: (**1**) (3.0 mol%), THF (0.25 M), 23 °C. All reactions carried out using standard Schlenk techniques under argon. [b]Determined by ^{1}H NMR and/or GC-MS.

Comparison of Reactivity using Cin-IPr

General Procedure. An oven-dried vial equipped with a stir bar was charged with an amide or ester substrate (neat, 0.20 mmol, 1.0 equiv), potassium carbonate, boronic acid, (**Cin-IPr**) (η^3-cinnamyl)Pd(IPr)(Cl) (3 mol%),[9] placed under a positive pressure of argon, and subjected to three evacuation/backfilling cycles under high vacuum. THF (0.25 M) was added with vigorous stirring and the reaction mixture was stirred at room temperature for 15 h. After the indicated time, the reaction mixture was diluted with CH_2Cl_2 (10 mL), filtered, and concentrated. The sample was analyzed by ^1H NMR ($CDCl_3$, 500 MHz) and/or GC-MS to obtain conversion, selectivity and yield using internal standard and comparison with authentic samples. Note that in all cases (**1**) significantly outperforms **Cin-IPr** in the coupling.[10]

Table SI-2. Suzuki-Miyaura Cross-Coupling Reactions at Room Temperature Catalyzed by **Cin-IPr**.[a]

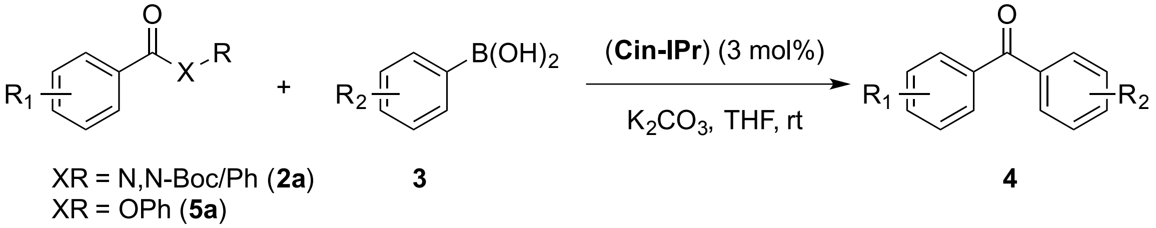

Entry	XR	(R$_1$)	(R$_2$)	**yield of 4** (%)[b]
1	(**2a**)	4-MeO-	H-	8
2	(**2a**)	4-CF_3-	H-	7
3	(**2a**)	4-CO_2Me-	H-	<2
4	(**5a**)	4-MeO-	H-	<2
5	(**5a**)	4-CF_3-	H-	<2
6	(**5a**)	4-CO_2Me-	H-	<2
7	(**2a**)	H-	4-CF_3-	6
8	(**2a**)	H-	4-Me-	28
9	(**5a**)	H-	4-CF_3-	<2
10	(**5a**)	H-	4-Me-	7

[a]Conditions: (**Cin-IPr**) (3.0 mol%), Ar-B(OH)$_2$ (**2a**: 3.0 equiv, **5a**: 4.5 equiv), K_2CO_3 (**2a**: 4.5 equiv, **5a**: 7.2 equiv), THF (0.25 M), 23 °C. All reactions carried out using standard Schlenk techniques under argon. [b]Determined by ^1H NMR and/or GC-MS.

Computational Methods

Computational Methods. All of the calculations were performed using Gaussian 09 suite of programs. All of the geometry optimizations were performed at the B3LYP/6-311++G(d,p**)** level of theory in the gas phase. This level has been shown to be accurate in predicting properties and resonance energies of carboxylic acid derivatives (*J. Org. Chem.* **2012**, *77*, 5492, and references cited therein). This method was further verified by obtaining good correlations between the calculated structures and X-ray structures in the series. All conformations within 3 kcal/mol from the lowest energy conformer were explored (*J. Am. Chem. Soc.* **1996**, *118*, 8658). The absence of imaginary frequencies was used to characterize the structures as minima on the potential energy surface. All of the optimized geometries were verified as minima (no imaginary frequencies). Electronic and thermal energies were calculated for all structures. Energetic parameters were calculated under standard conditions (298.15 K and 1 atm). For geometry optimizations, we employed the structure of 4-bromophenyl benzoate as the starting geometry and performed full optimization (*Acta Crystallogr. Sect. E* **2008**, *64*, 771). Barrier to rotation in methyl acetate has been reported (13.0 kcal/mol, *J. Am. Chem. Soc.* **1987**, *109*, 5935). Phenyl benzoate isomerization barrier was determined by ester bond rotation. COSNAR method was used for determination of amide resonance energies (*J. Am. Chem. Soc.* **1996**, *118*, 8658, *J. Org. Chem.* **2016**, *81*, 8091). Optimized amide conformations were used as starting geometries for isodesmic calculations for the aza, keto, and hydrocarbon derivatives. Structural representations were generated using CYLview software (Legault, C. Y. CYLview version 1.0 BETA, University of Sherbrooke). All other representations were generated using GaussView (GaussView, version 5, Dennington, R.; Keith, T.; Millam, J. Semichem Inc., Shawnee Mission, KS, 2009).

Full Reference for Gaussian 09

Gaussian 09, Revision D.01, Frisch, M. J.; Trucks, G. W.; Schlegel, H. B.; Scuseria, G. E.; Robb, M. A.; Cheeseman, J. R.; Scalmani, G.; Barone, V.; Mennucci, B.; Petersson, G. A.; Nakatsuji, H.; Caricato, M.; Li, X.; Hratchian, H. P.; Izmaylov, A. F.; Bloino, J.; Zheng, G.; Sonnenberg, J. L.; Hada, M.; Ehara, M.; Toyota, K.; Fukuda, R.; Hasegawa, J.; Ishida, M.; Nakajima, T.; Honda, Y.; Kitao, O.; Nakai, H.; Vreven, T.; Montgomery, J. A., Jr.; Peralta, J. E.; Ogliaro, F.; Bearpark, M.; Heyd, J. J.; Brothers, E.; Kudin, K. N.; Staroverov, V. N.; Kobayashi, R.; Normand, J.; Raghavachari, K.; Rendell, A.; Burant, J. C.; Iyengar, S. S.; Tomasi, J.; Cossi, M.; Rega, N.; Millam, M. J.; Klene, M.; Knox, J. E.; Cross, J. B.; Bakken, V.; Adamo, C.; Jaramillo, J.; Gomperts, R.; Stratmann, R. E.; Yazyev, O.; Austin, A. J.; Cammi, R.; Pomelli, C.; Ochterski, J. W.; Martin, R. L.; Morokuma, K.; Zakrzewski, V. G.; Voth, G. A.; Salvador, P.; Dannenberg, J. J.; Dapprich, S.; Daniels, A. D.; Farkas, Ö.; Foresman, J. B.; Ortiz, J. V.; Cioslowski, J.; Fox, D. J. Gaussian, Inc., Wallingford CT, 2009.

Figure SI-1. Optimized Geometry of 5a Referred to from the Main Manuscript

*Ester **5a** (B3LYP/6-311++G(d,p))*

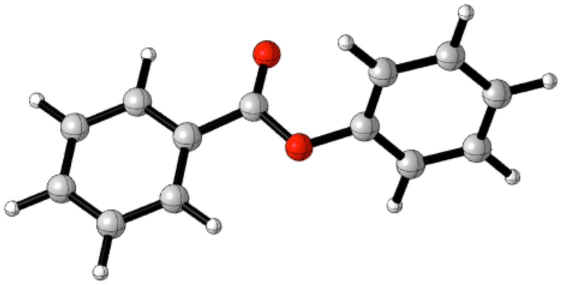

Selected bond lengths (Å) and angles (deg): C1–C2–O1–C3 = 179.90; O1–C2–O2–C3 = 0.0; C5–C3–O1–C2 = 65.33; C4–C3–O1–C2 = -118.58; O1–C2, 1.370; C2–O2, 1.204; C1–C2, 1.489; O1–C3, 1.399.

Plot of Rotational Barrier Referred to from the Main Manuscript

*Rotational profile of ester **5a***

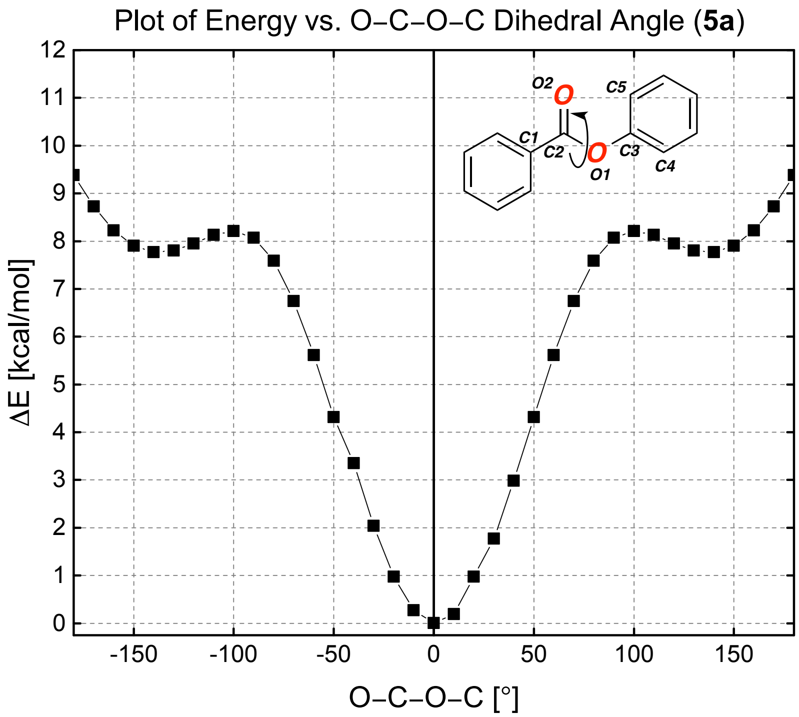

Chart SI-1. Correlation of ΔE [kcal/mol] to O–C–O–C [°] in **5a**. Note that barrier to C–O isomerization in methyl benzoate (12.8 kcal/mol, B3LYP/6-311++G(d,p)) is similar to anilides (12-14 kcal/mol, B3LYP/6-311++G(d,p)).

References

1. a) Meng, G.; Szostak, M. *Angew. Chem. Int. Ed.* **2015**, *54*, 14518. b) Meng, G.; Szostak, M. *Org. Lett.* **2016**, *18*, 796. c) Shi, S.; Meng, G.; Szostak, M. *Angew. Chem. Int. Ed.* **2016**, *55*, 6959. d) Meng, G.; Szostak, M. *Org. Lett.* **2015**, *17*, 4364.
2. a) Weires, N. A.; Baker, E. L.; Garg, N. K. *Nat. Chem.* **2016**, 8, 75. b) Baker, E. L.; Yamano, M. M.; Zhou, Y.; Anthony, S. M.; Garg, N. K. *Nat. Commun.* **2016**, *7*, 11554. c) Simmons, B. J.; Weires, N. A.; Dander, J. E.; Garg, N. K. *ACS Catal.* **2016**, *6*, 3176.
3. Li, X.; Zou, G. *Chem. Commun.* **2015**, *51*, 5089.
4. a) Muto, K.; Yamaguchi, J.; Musaev, D. G.; Itami, K. *Nat. Commun.* **2015**, *6*, 7508. b) Halima, T. B.; Zhang, W.; Yalaoui, I.; Hong, X.; Yang, Y. F.; Houk, K. N.; Newman, S. G. *J. Am. Chem. Soc.* **2017**, *139*, 1311.
5. Melvin, P. R.; Nova, A.; Balcells, D.; Dai, W.; Hazari, N.; Hruszkewycz, D. P.; Shah, H. P.; Tudge, M. T. *ACS Catal.* **2015**, *5*, 3680.
6. Espenson, J. H. *Chemical Kinetics and Reaction Mechanisms*, McGraw-Hill, **2002**.
7. Meng, G.; Shi, S.; Szostak, M. *Synlett* **2016**, *27*, 2530.
8. a) Liebman, J.; Greenberg, A. *Biophys. Chem.* **1974**, *1*, 222. b) Greenberg, A.; Breneman, C. M.; Liebman, J. F. *The Amide Linkage: Structural Significance in Chemistry, Biochemistry and Materials Science*, Wiley, **2003**.
9. Lei, P.; Meng, G.; Szostak, M. *ACS Catal.* **2017**, *7*, 1960.
10. Note that, in general, strong bases such as KO*t*-Bu are not viable in the coupling of amides and esters catalyzed by Pd-NHC complexes.

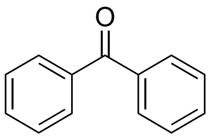

4a

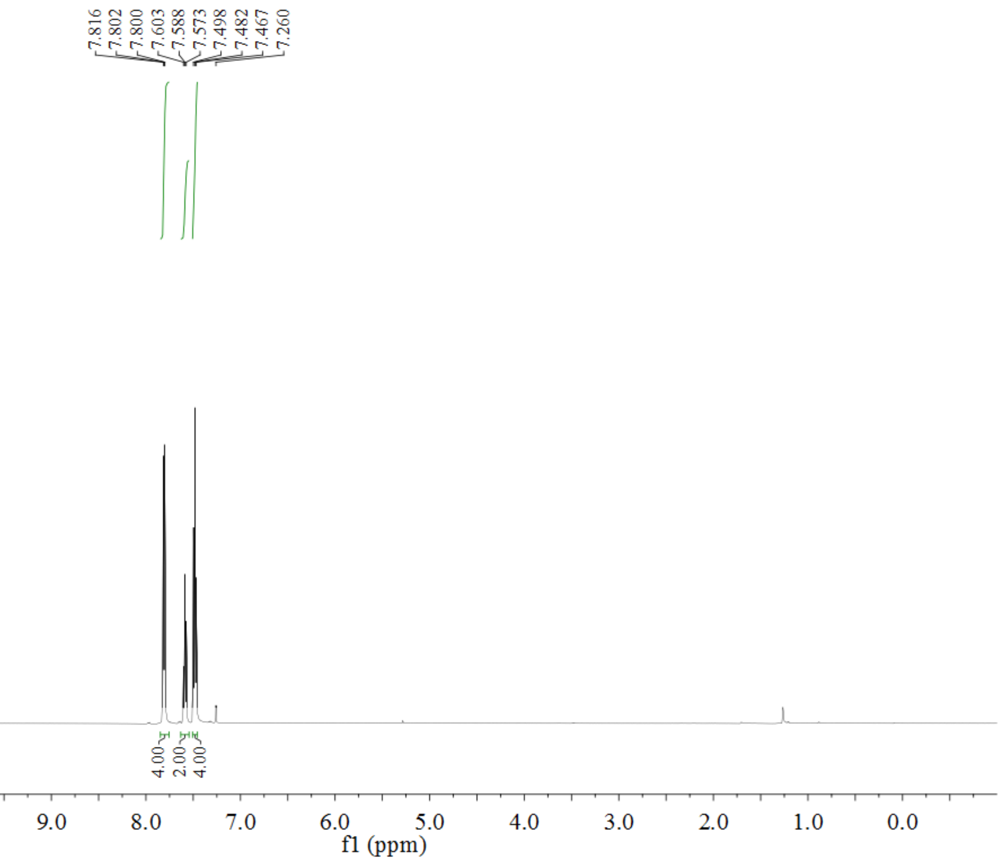
7.816
7.802
7.800
7.603
7.588
7.573
7.498
7.482
7.467
7.260
4.00
2.00
4.00
11.0 10.0 9.0 8.0 7.0 6.0 5.0 4.0 3.0 2.0 1.0 0.0
f1 (ppm)

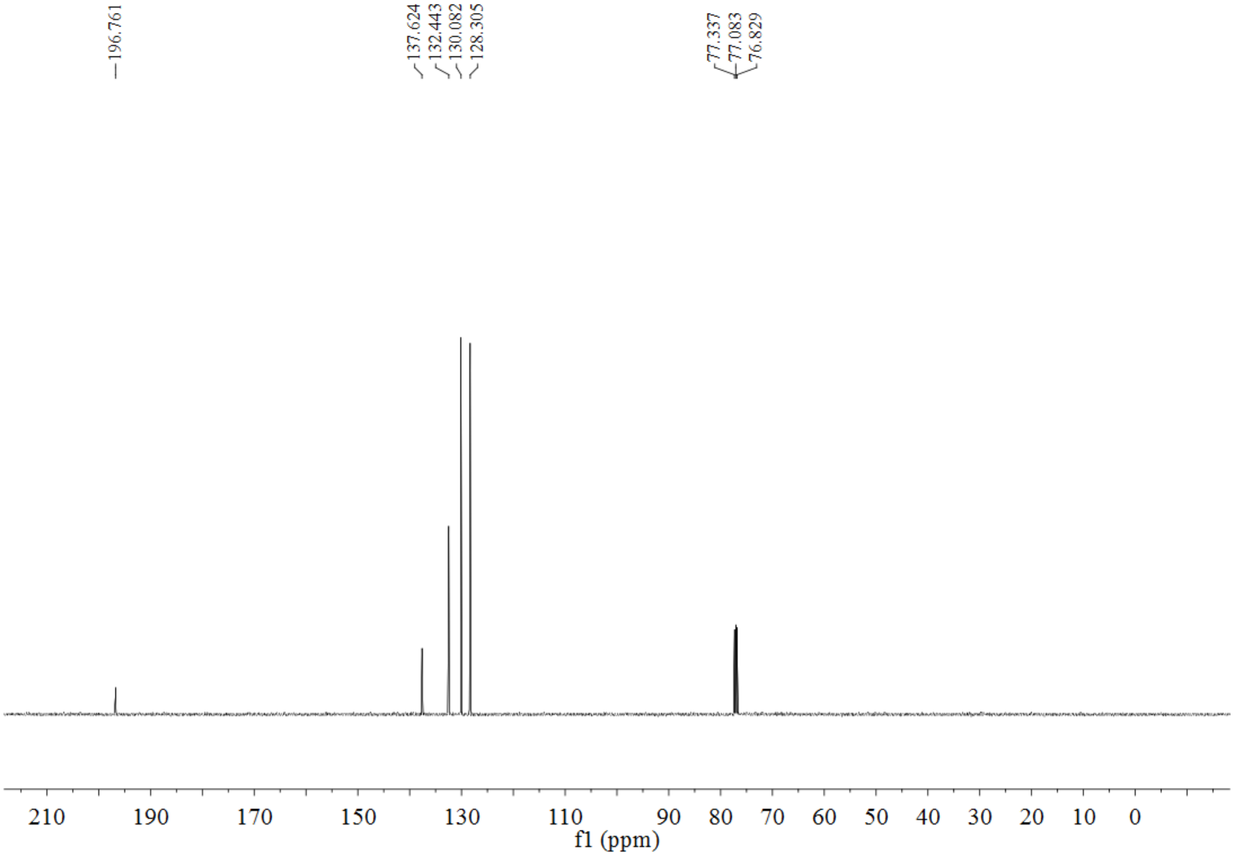
196.761
137.624
132.443
130.082
128.305
77.337
77.083
76.829
210 190 170 150 130 110 90 80 70 60 50 40 30 20 10 0
f1 (ppm)

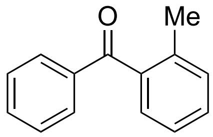
O
Me

4b

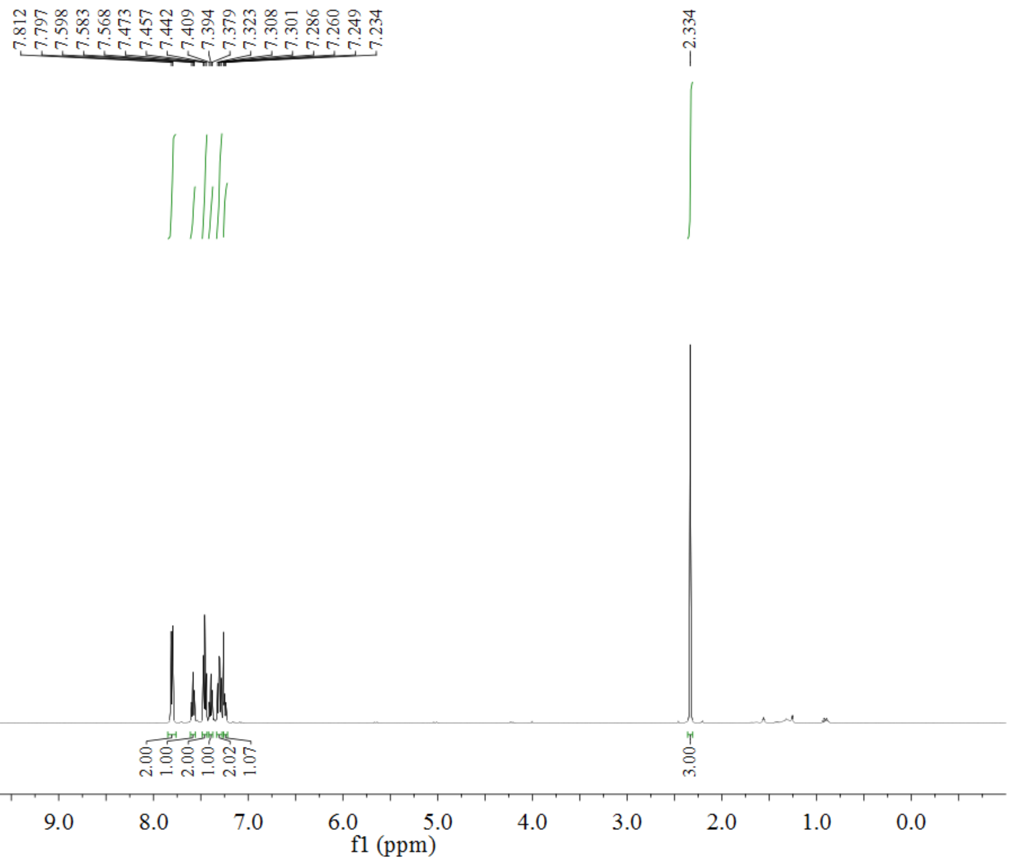
7.812
7.797
7.598
7.583
7.568
7.473
7.457
7.442
7.409
7.394
7.379
7.323
7.308
7.301
7.286
7.260
7.249
7.234
2.334
2.00
1.00
2.00
1.00
2.02
1.07
3.00
11.0
10.0
9.0
8.0
7.0
6.0
5.0
4.0
3.0
2.0
1.0
0.0
f1 (ppm)

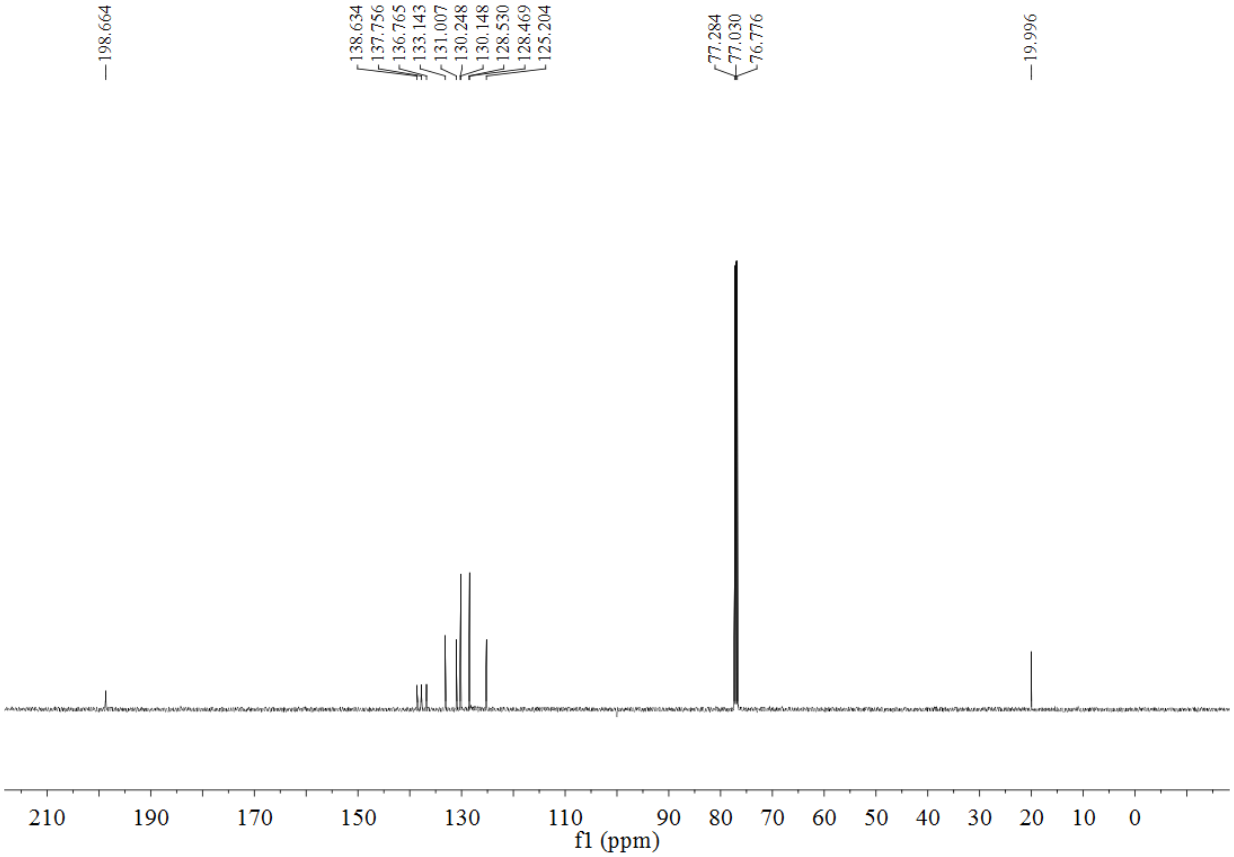
198.664
138.634
137.756
136.765
133.143
131.007
130.248
130.148
128.530
128.469
125.204
77.284
77.030
76.776
19.996
210
190
170
150
130
110
90
80
70
60
50
40
30
20
10
0
f1 (ppm)

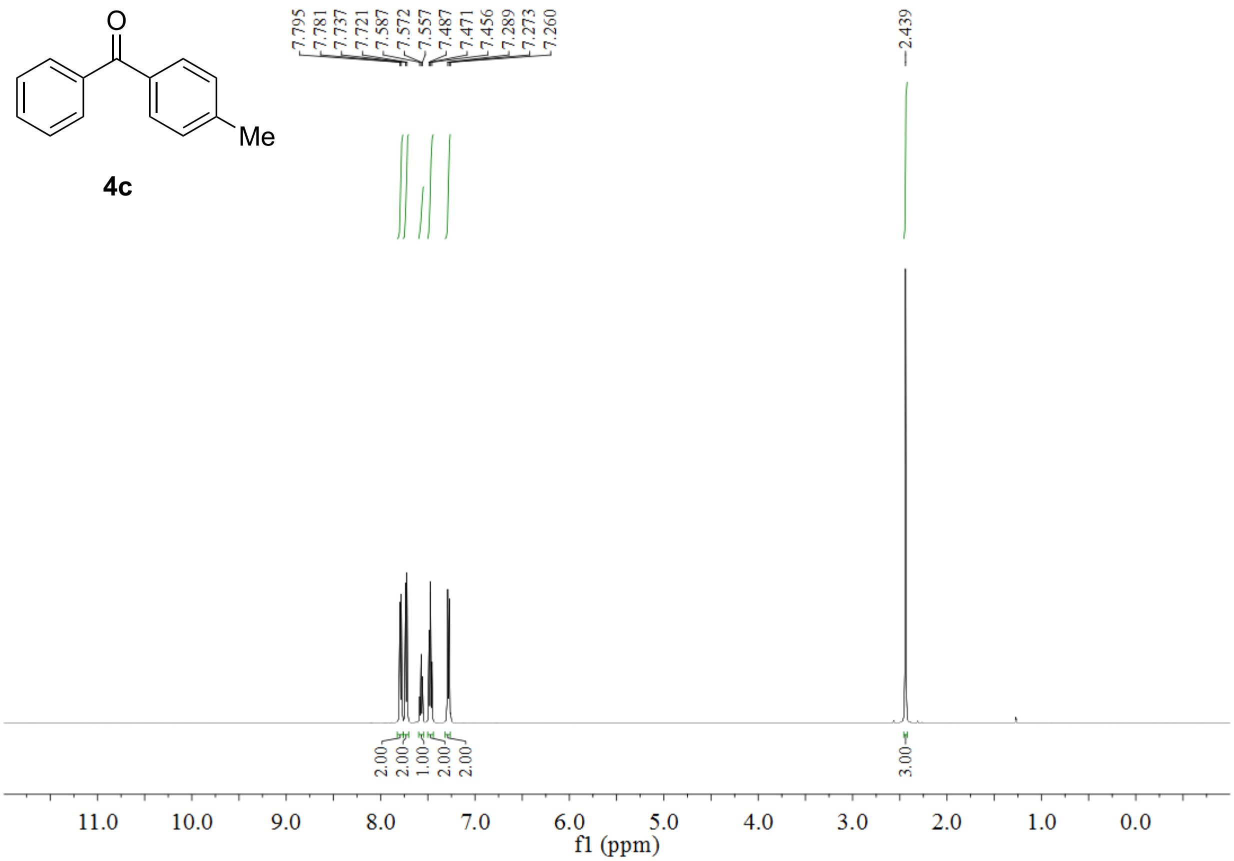
O
Me
4c
7.795
7.781
7.737
7.721
7.587
7.572
7.557
7.487
7.471
7.456
7.289
7.273
7.260
2.439
2.00
2.00
1.00
2.00
2.00
3.00
11.0
10.0
9.0
8.0
7.0
6.0
5.0
4.0
3.0
2.0
1.0
0.0
f1 (ppm)

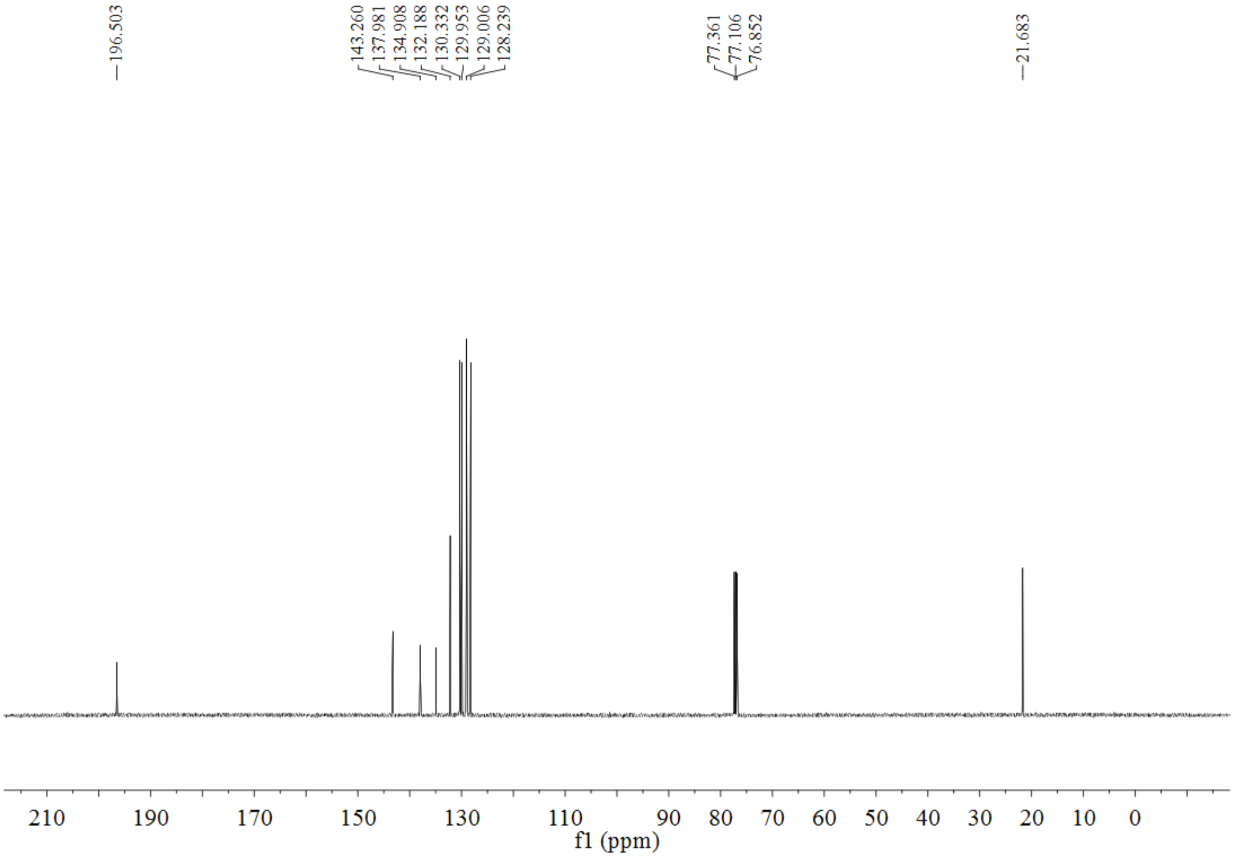
196.503
143.260
137.981
134.908
132.188
130.332
129.953
129.006
128.239
77.361
77.106
76.852
21.683
210
190
170
150
130
110
90
80
70
60
50
40
30
20
10
0
f1 (ppm)

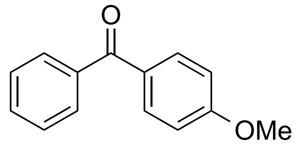
OMe

4d

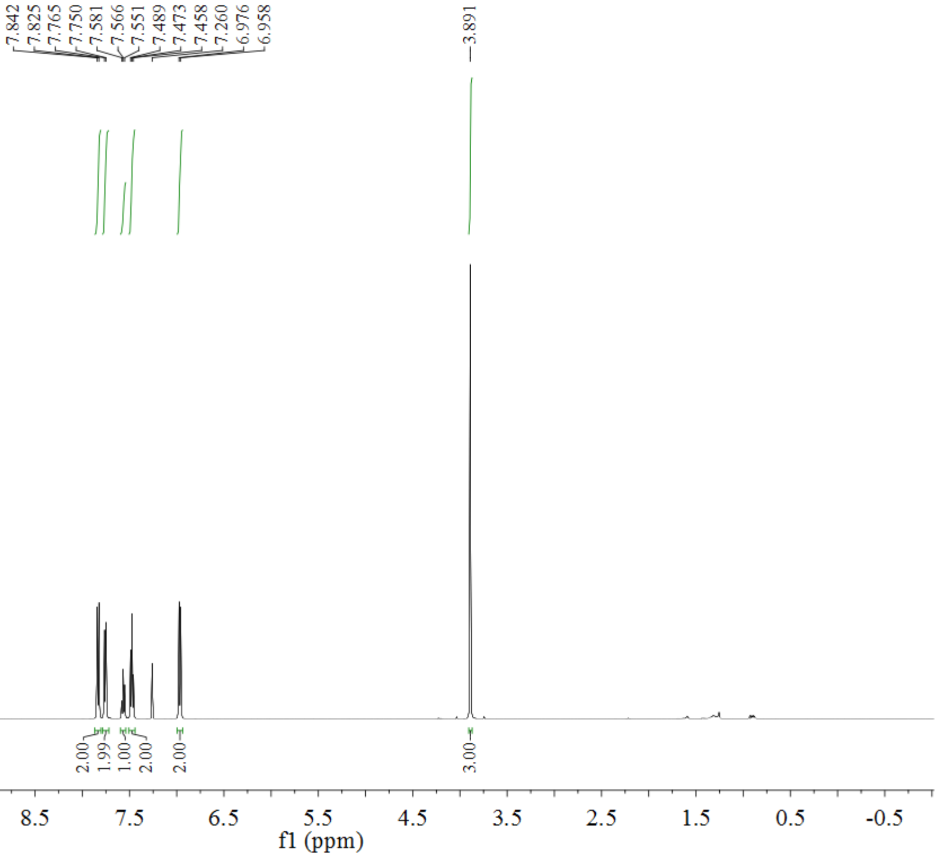
7.842
7.825
7.765
7.750
7.581
7.566
7.551
7.489
7.473
7.458
7.260
6.976
6.958
3.891
2.00
1.99
1.00
2.00
2.00
3.00
11.5
10.5
9.5
8.5
7.5
6.5
5.5
4.5
3.5
2.5
1.5
0.5
-0.5
f1 (ppm)

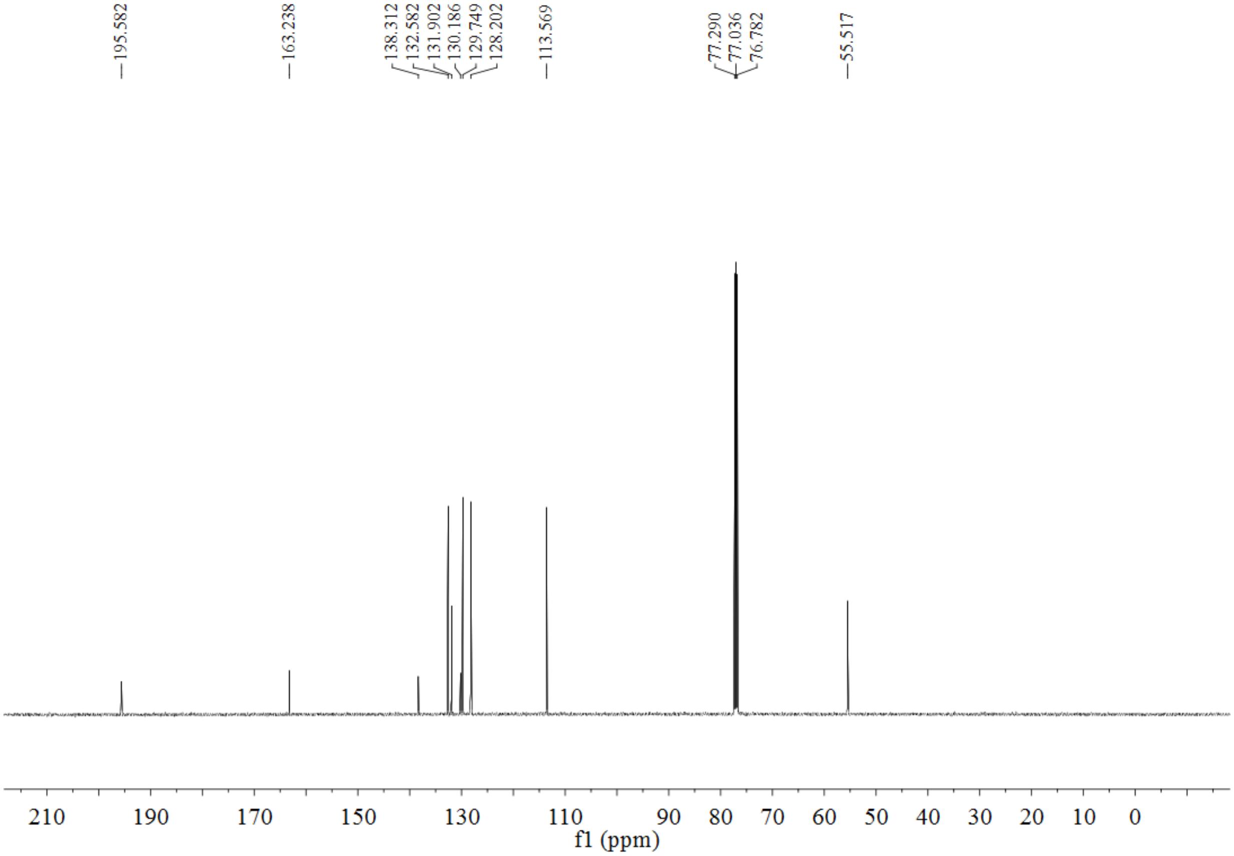
195.582
163.238
138.312
132.582
131.902
130.186
129.749
128.202
113.569
77.290
77.036
76.782
55.517
210
190
170
150
130
110
90
80
70
60
50
40
30
20
10
0
f1 (ppm)

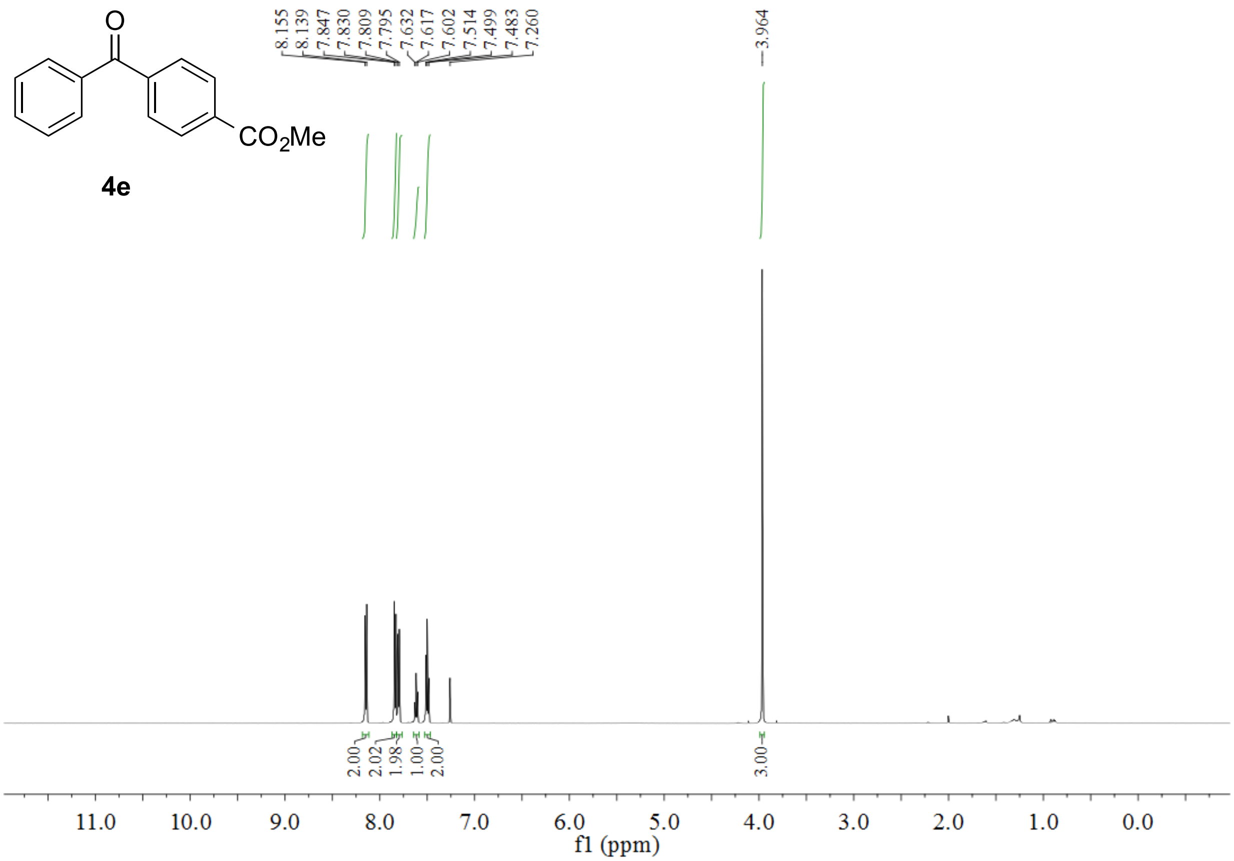

CO_2Me
4e
8.155
8.139
7.847
7.830
7.809
7.795
7.632
7.617
7.602
7.514
7.499
7.483
7.260
3.964
2.00
2.02
1.98
1.00
2.00
3.00
11.0 10.0 9.0 8.0 7.0 6.0 5.0 4.0 3.0 2.0 1.0 0.0
f1 (ppm)

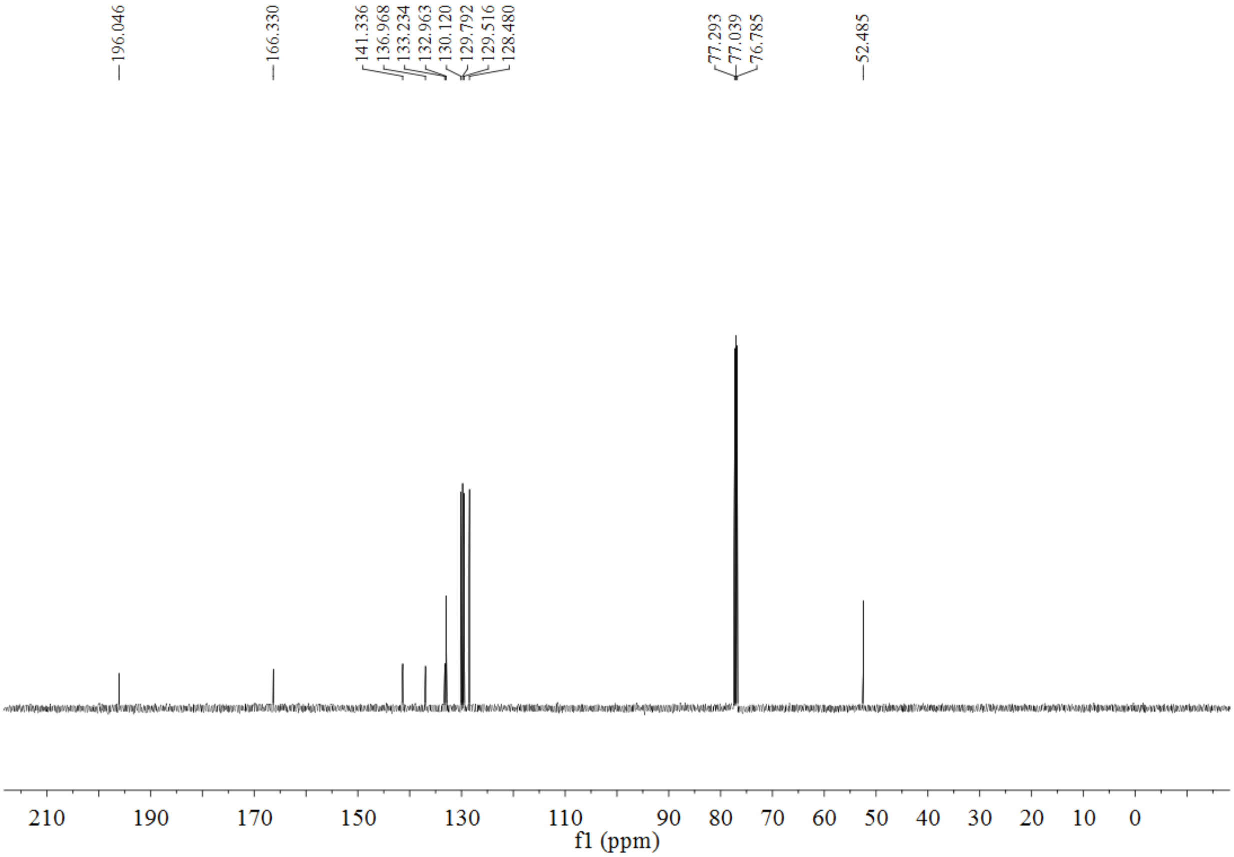

196.046
166.330
141.336
136.968
133.234
132.963
130.120
129.792
129.516
128.480
77.293
77.039
76.785
52.485
210 190 170 150 130 110 90 80 70 60 50 40 30 20 10 0
f1 (ppm)

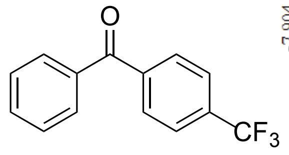

4f

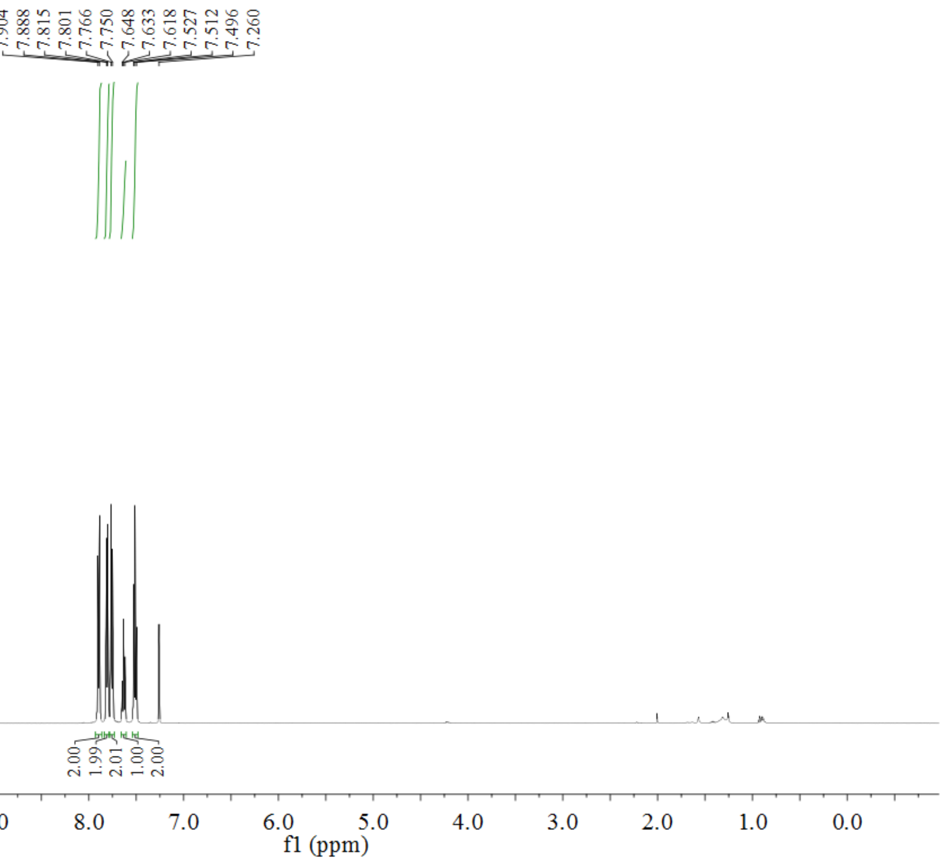
7.904
7.888
7.815
7.801
7.766
7.750
7.648
7.633
7.618
7.527
7.512
7.496
7.260
2.00
1.99
2.01
1.00
2.00
11.0
10.0
9.0
8.0
7.0
6.0
5.0
4.0
3.0
2.0
1.0
0.0
f1 (ppm)

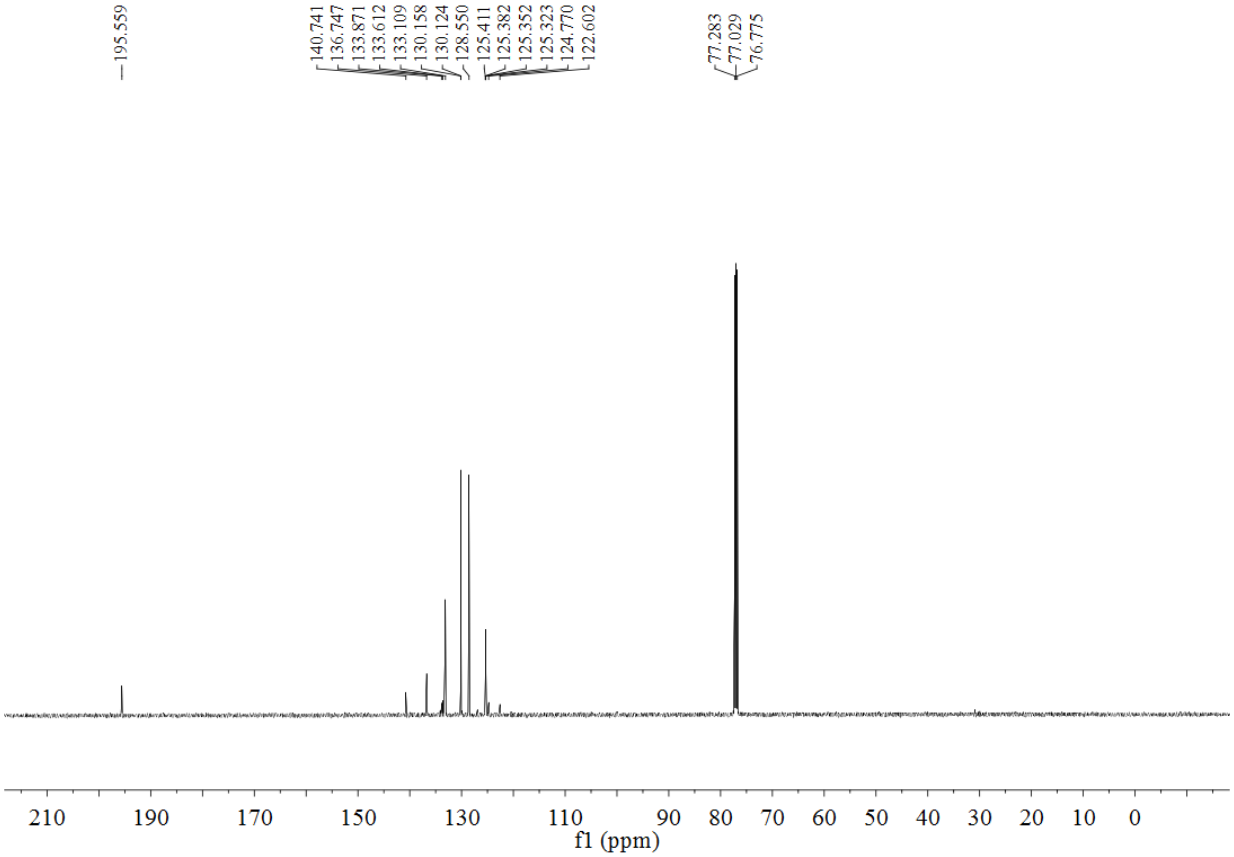
195.559
140.741
136.747
133.871
133.612
133.109
130.158
130.124
128.550
125.411
125.382
125.352
125.323
124.770
122.602
77.283
77.029
76.775
210
190
170
150
130
110
90
80
70
60
50
40
30
20
10
0
f1 (ppm)

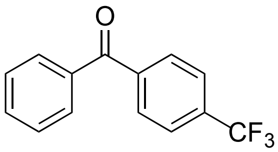

4f

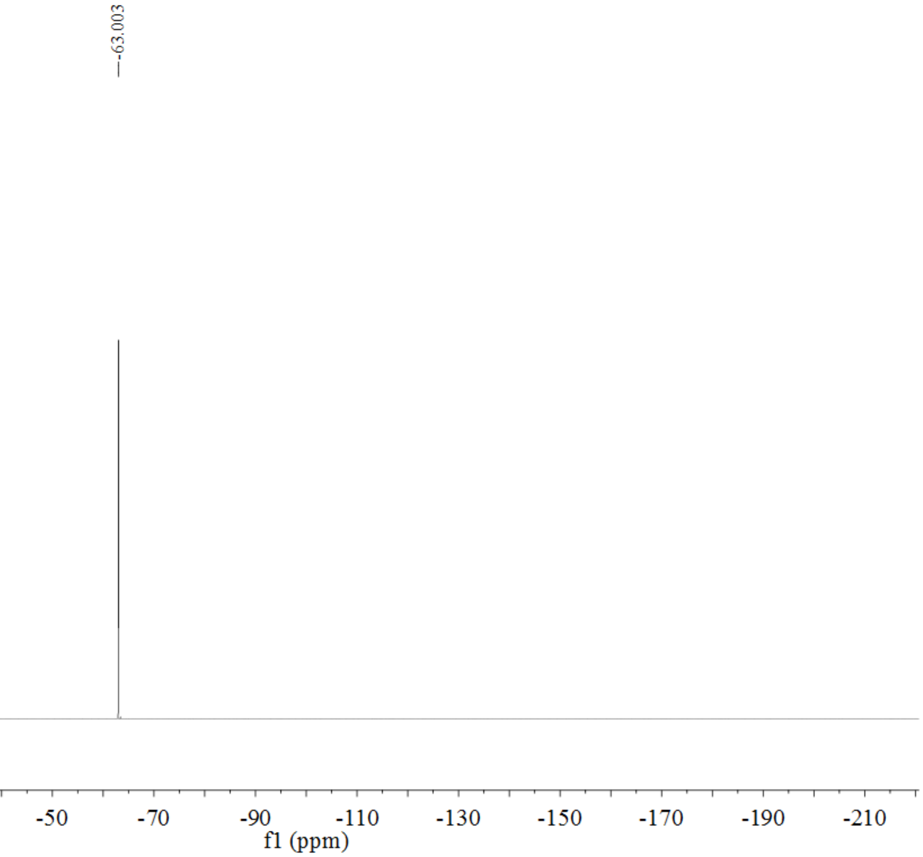

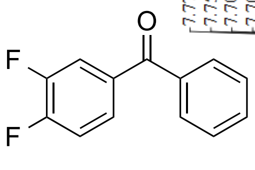
O
F
F

4g

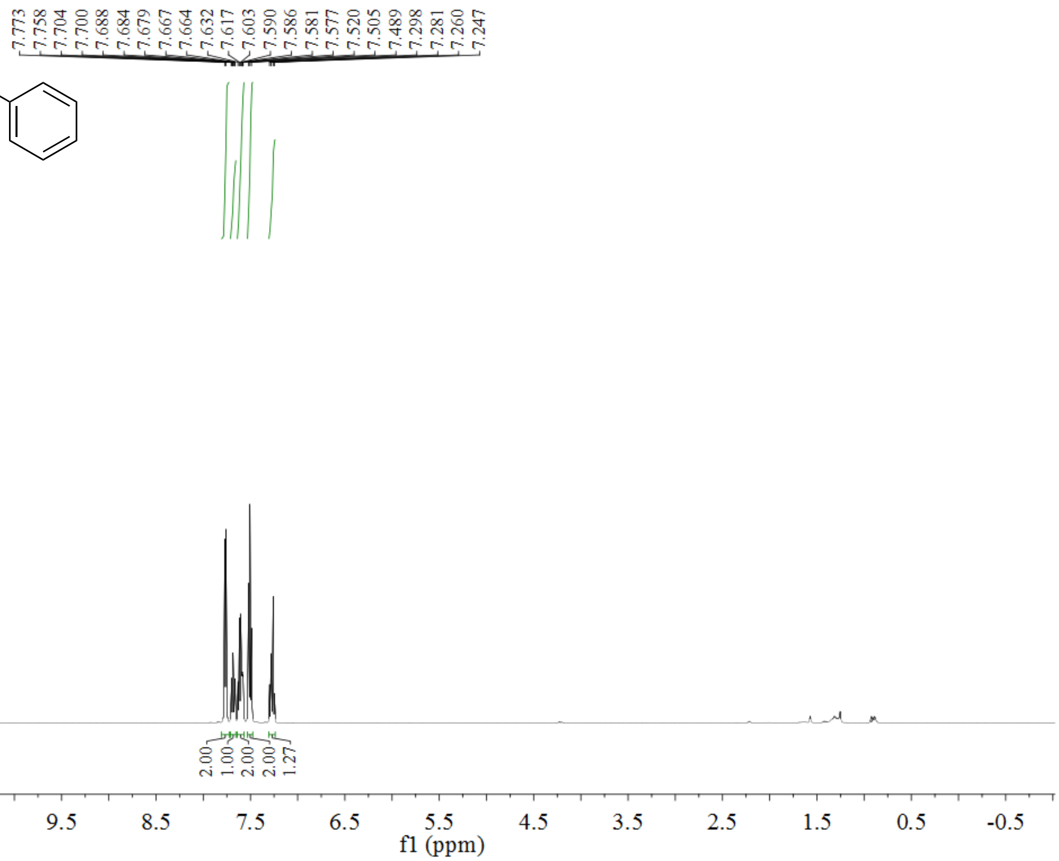
7.773
7.758
7.704
7.700
7.688
7.684
7.679
7.667
7.664
7.632
7.617
7.603
7.590
7.586
7.581
7.577
7.520
7.505
7.489
7.298
7.281
7.260
7.247
2.00
1.00
2.00
2.00
1.27
11.5 10.5 9.5 8.5 7.5 6.5 5.5 4.5 3.5 2.5 1.5 0.5 -0.5
f1 (ppm)

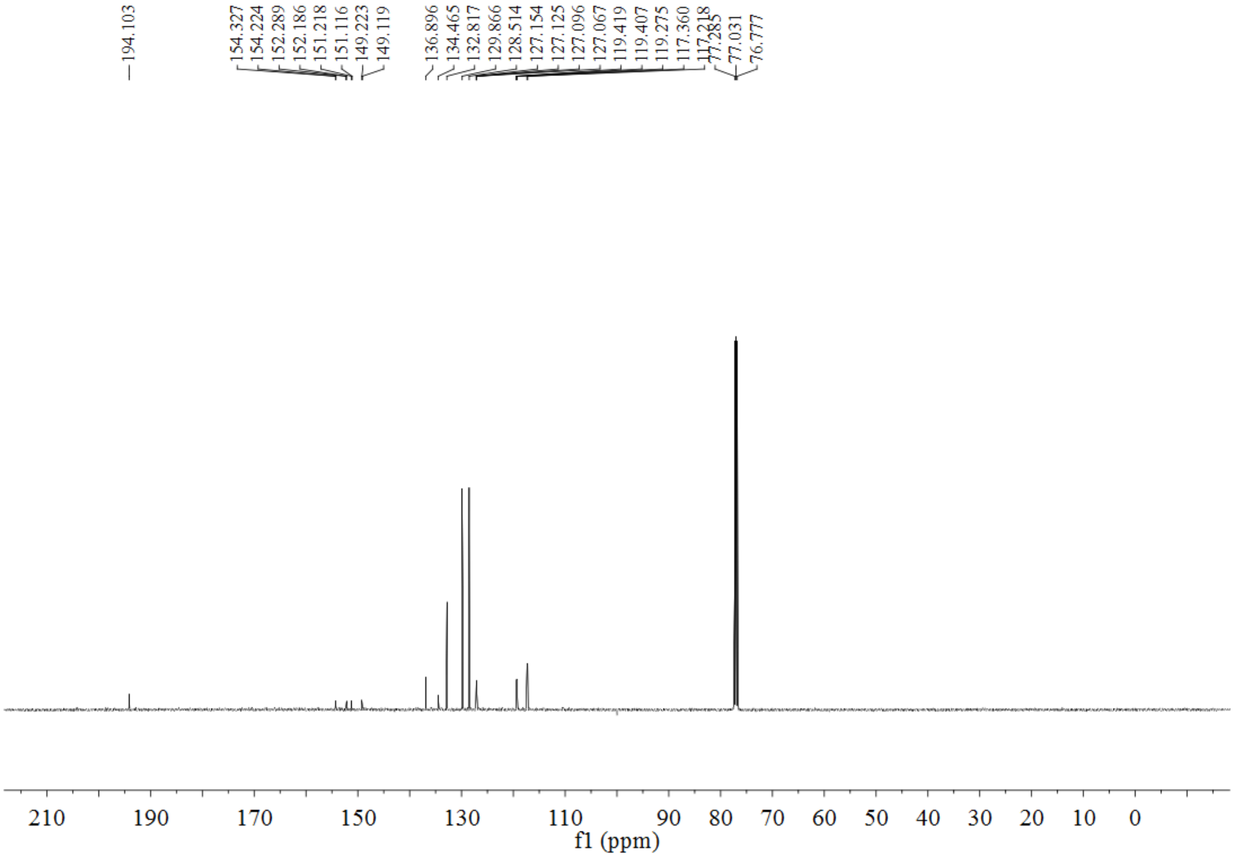
194.103
154.327
154.224
152.289
152.186
151.218
151.116
149.223
149.119
136.896
134.465
132.817
129.866
128.514
127.154
127.125
127.096
127.067
119.419
119.407
119.275
117.360
117.218
77.285
77.031
76.777
210 190 170 150 130 110 90 80 70 60 50 40 30 20 10 0
f1 (ppm)

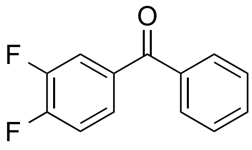

4g

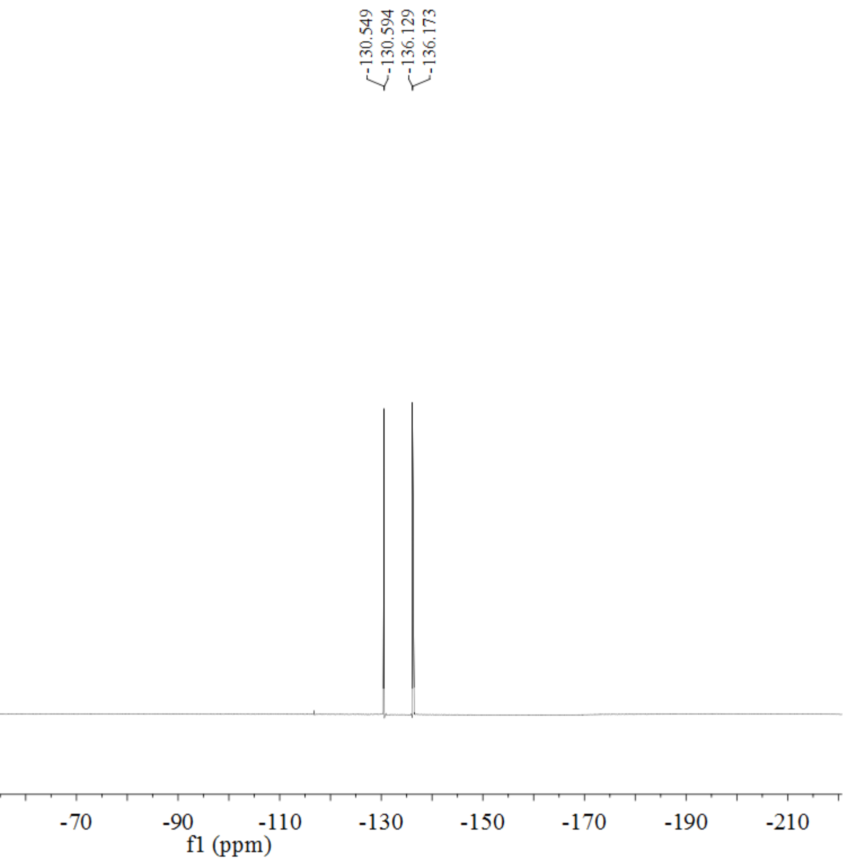
-130.549
-130.594
-136.129
-136.173
20 10 0 -10 -30 -50 -70 -90 -110 -130 -150 -170 -190 -210
f1 (ppm)

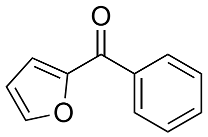

4h

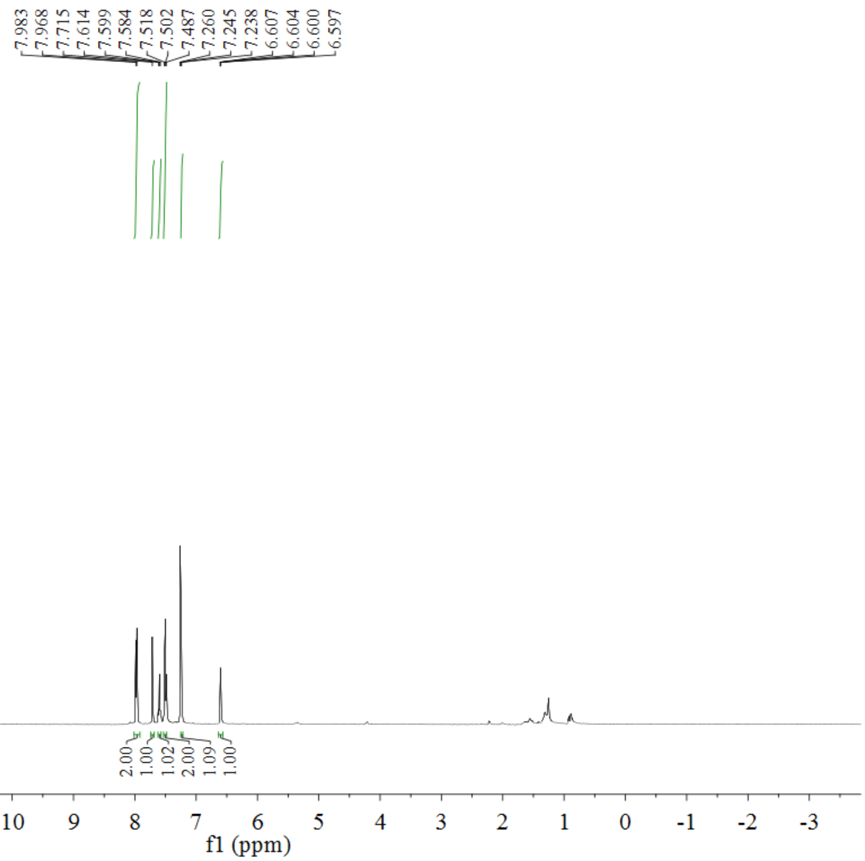
7.983
7.968
7.715
7.614
7.599
7.584
7.518
7.502
7.487
7.260
7.245
7.238
6.607
6.604
6.600
6.597
2.00
1.00
1.02
2.00
1.09
1.00
16 15 14 13 12 11 10 9 8 7 6 5 4 3 2 1 0 -1 -2 -3
f1 (ppm)

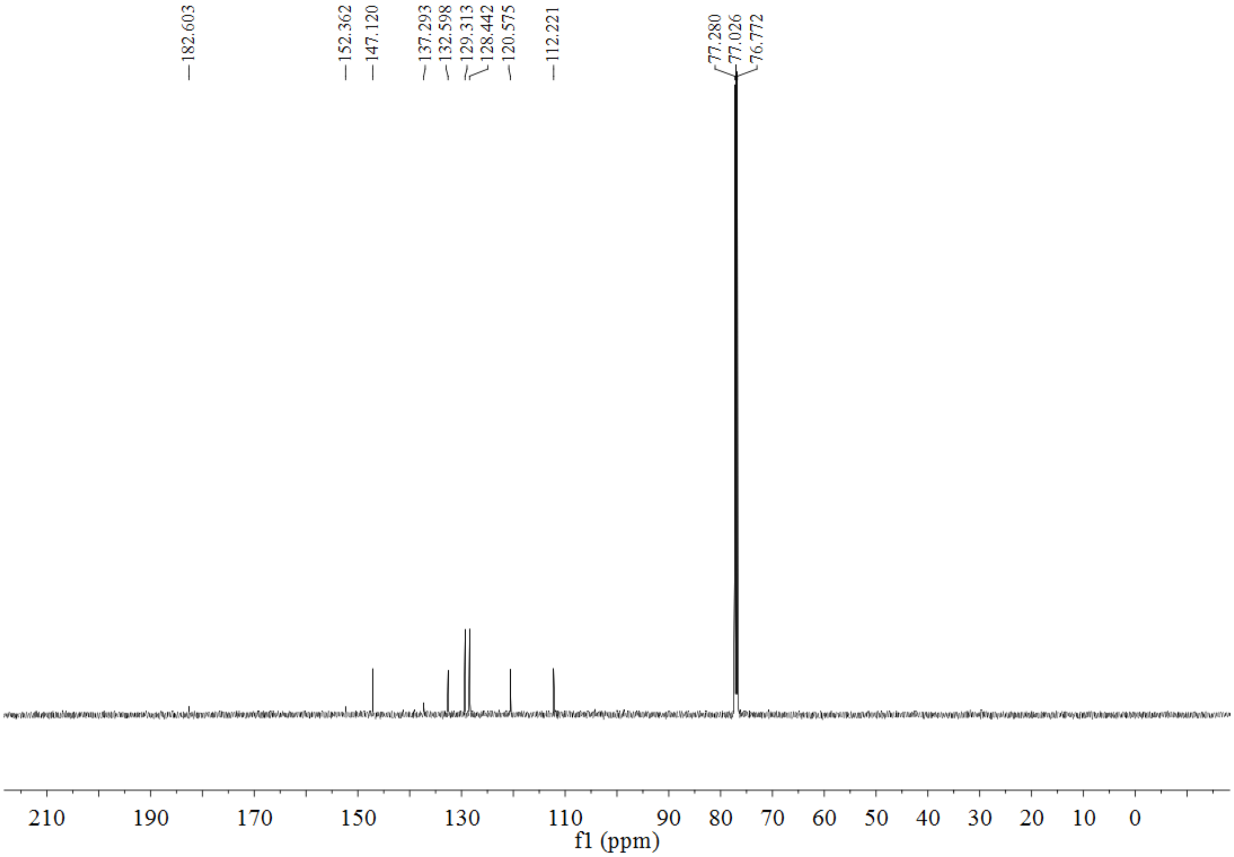
182.603
152.362
147.120
137.293
132.598
129.313
128.442
120.575
112.221
77.280
77.026
76.772
210 190 170 150 130 110 90 80 70 60 50 40 30 20 10 0
f1 (ppm)

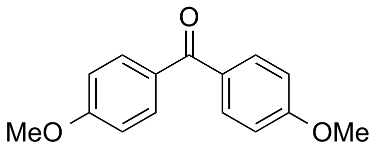

4i

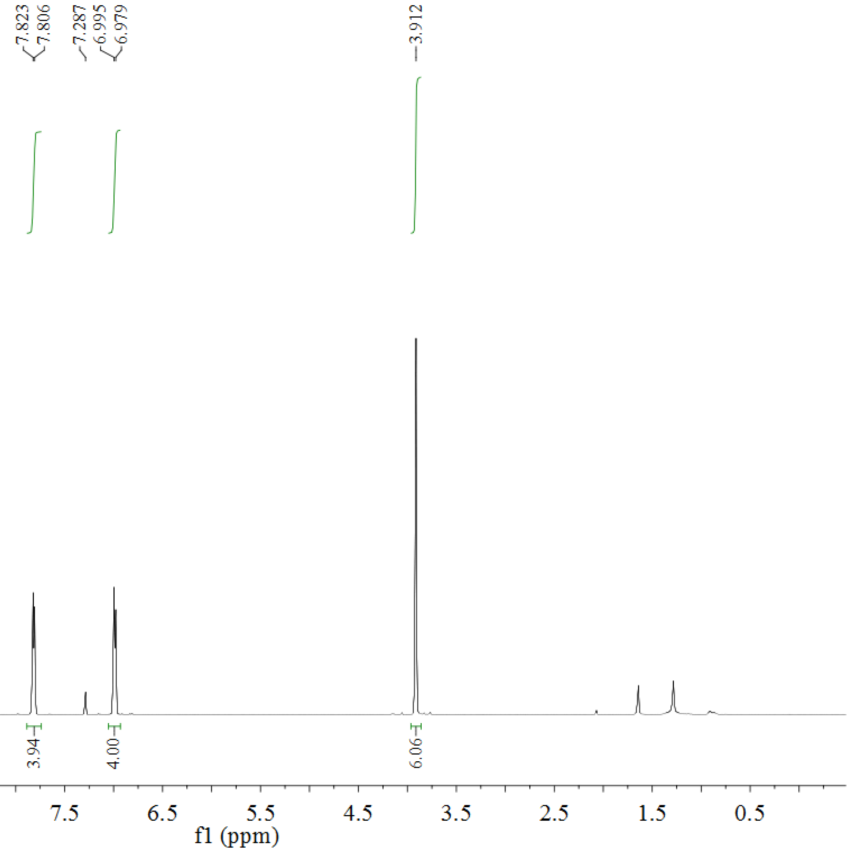
7.823
7.806
7.287
6.995
6.979
3.912
3.94
4.00
6.06
11.5
10.5
9.5
8.5
7.5
6.5
5.5
4.5
3.5
2.5
1.5
0.5
f1 (ppm)

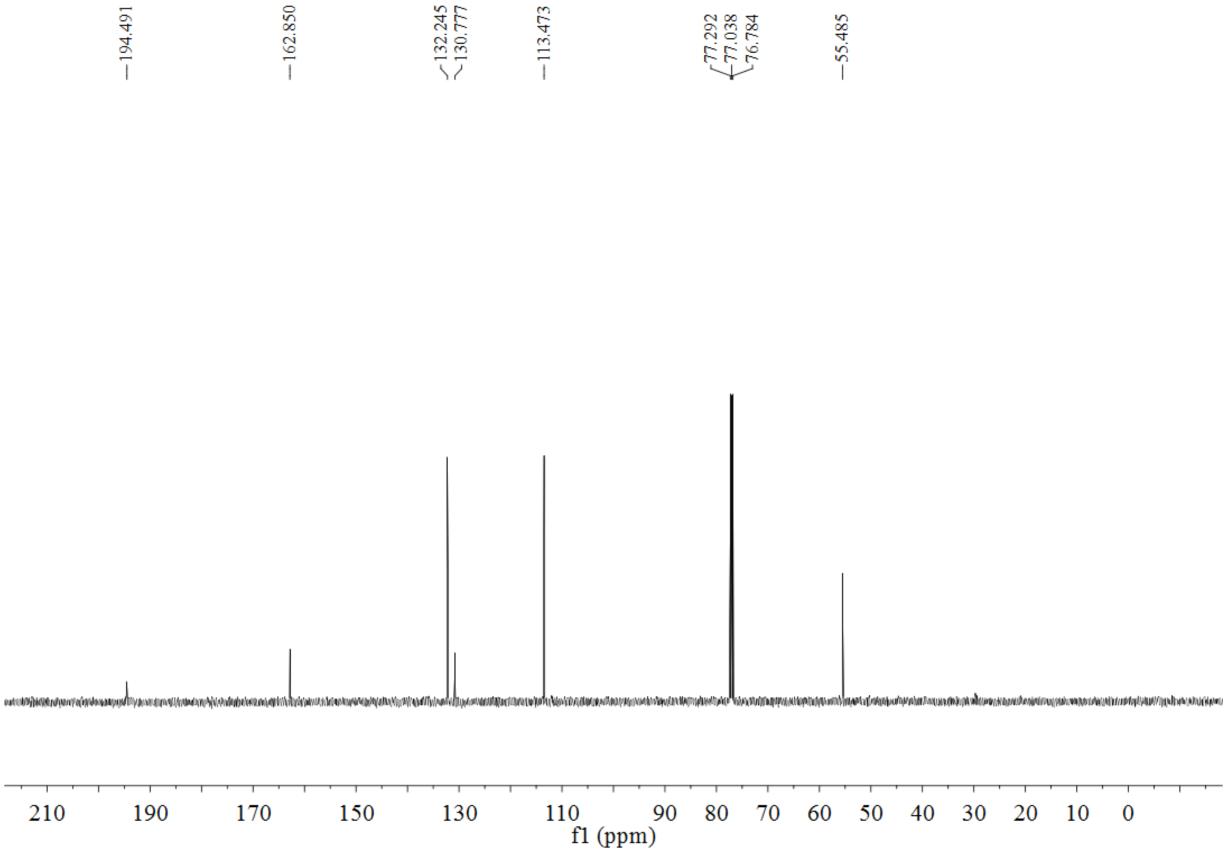
194.491
162.850
132.245
130.777
113.473
77.292
77.038
76.784
55.485
210
190
170
150
130
110
90
80
70
60
50
40
30
20
10
0
f1 (ppm)

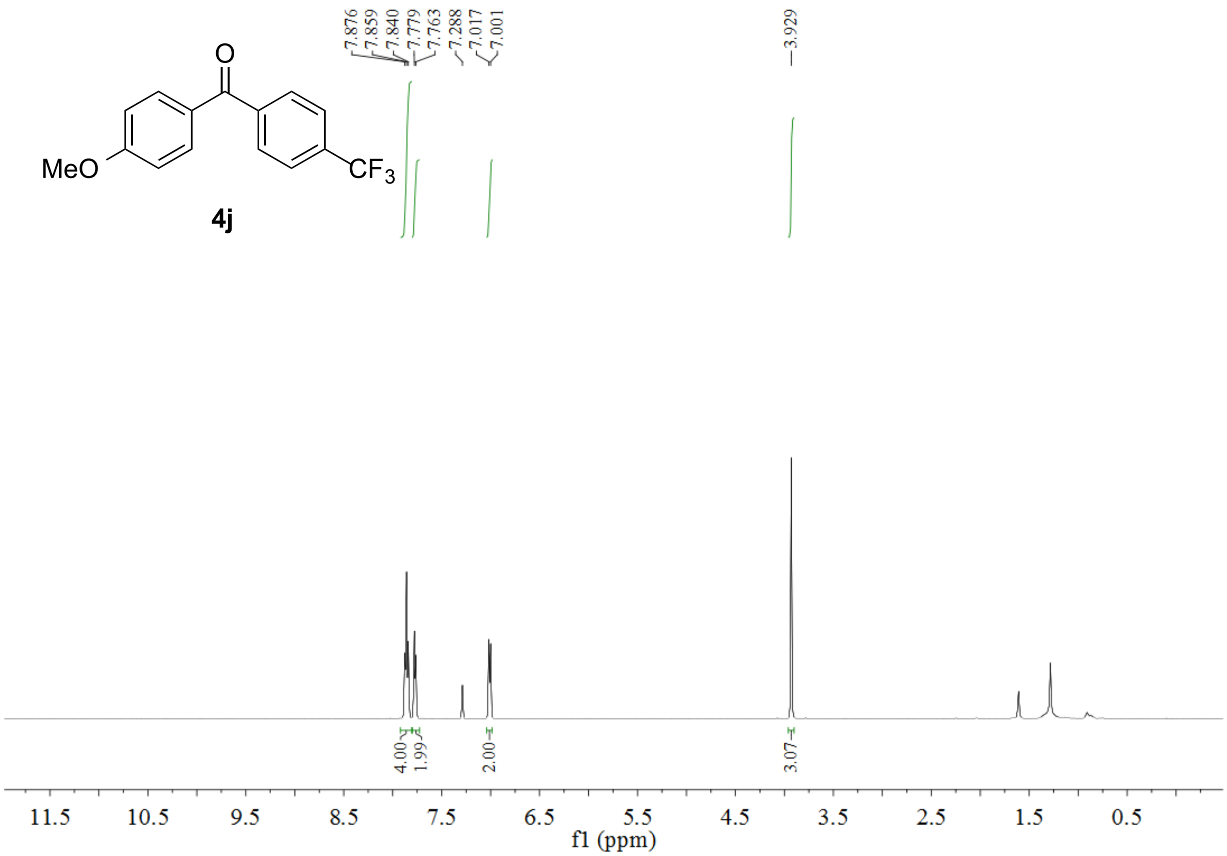
MeO
CF_3
O
4j
7.876
7.859
7.840
7.779
7.763
7.288
7.017
7.001
3.929
4.00
1.99
2.00
3.07
11.5 10.5 9.5 8.5 7.5 6.5 5.5 4.5 3.5 2.5 1.5 0.5
f1 (ppm)

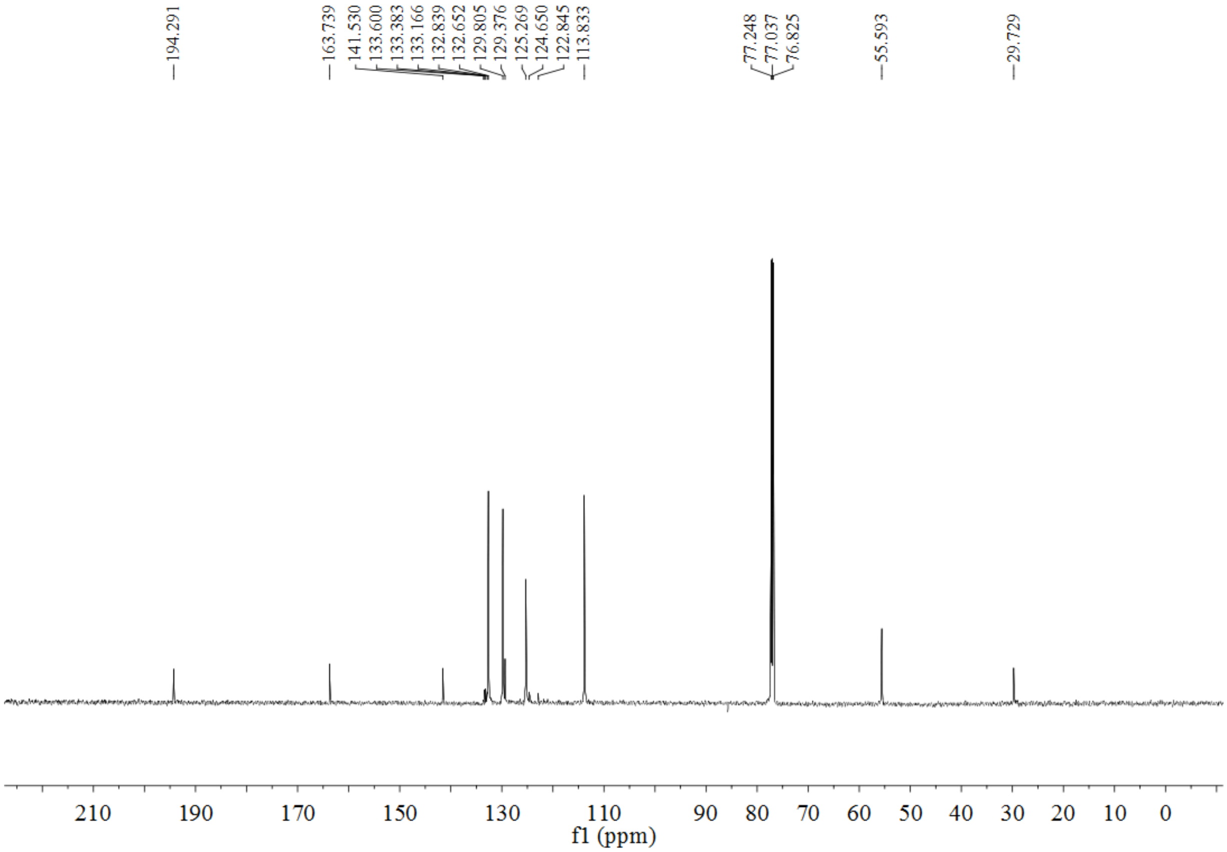
194.291
163.739
141.530
133.600
133.383
133.166
132.839
132.652
129.805
129.376
125.269
124.650
122.845
113.833
77.248
77.037
76.825
55.593
29.729
210 190 170 150 130 110 90 80 70 60 50 40 30 20 10 0
f1 (ppm)

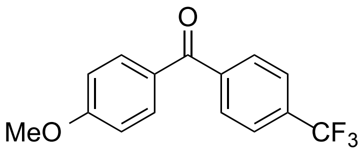

4j

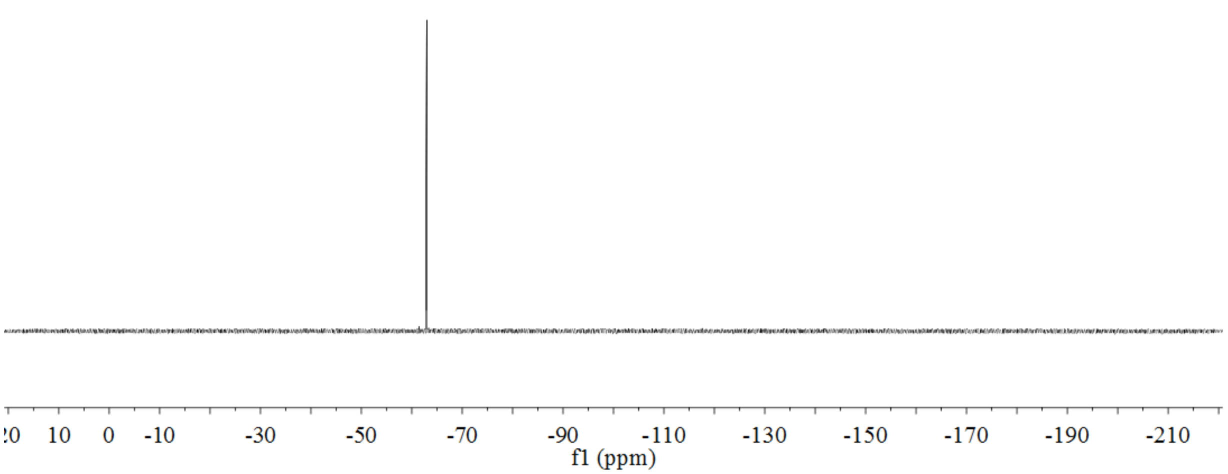

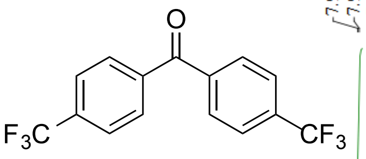
O
F_3C
CF_3

4k

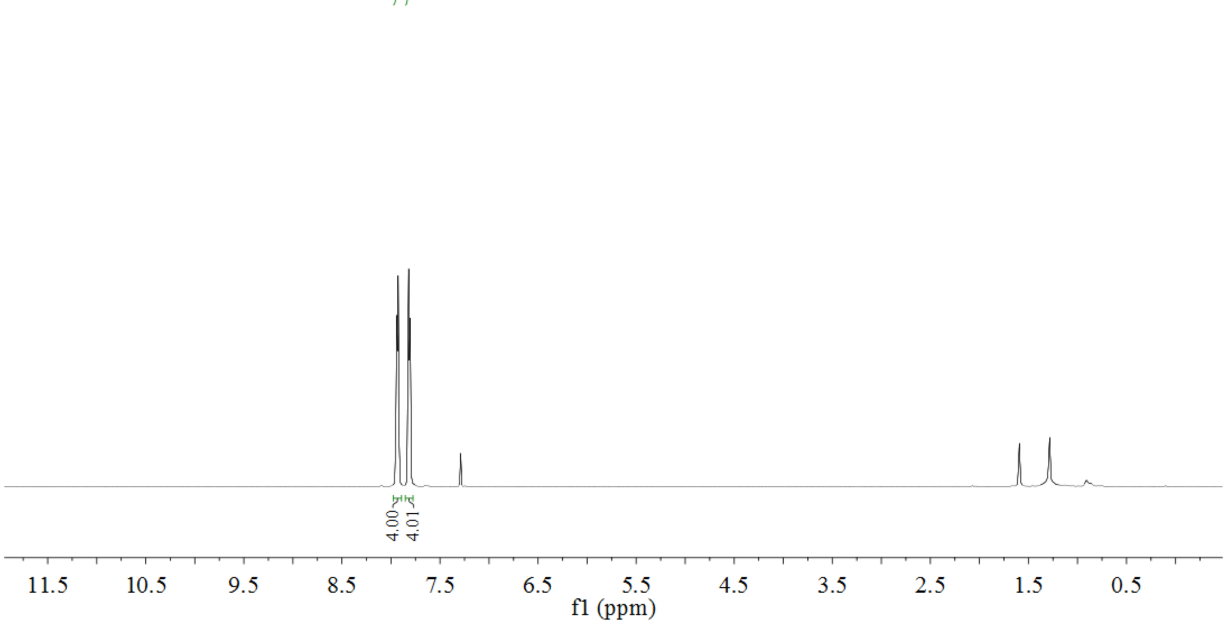
7.941
7.926
7.820
7.805
7.289
4.00
4.01
11.5 10.5 9.5 8.5 7.5 6.5 5.5 4.5 3.5 2.5 1.5 0.5
f1 (ppm)

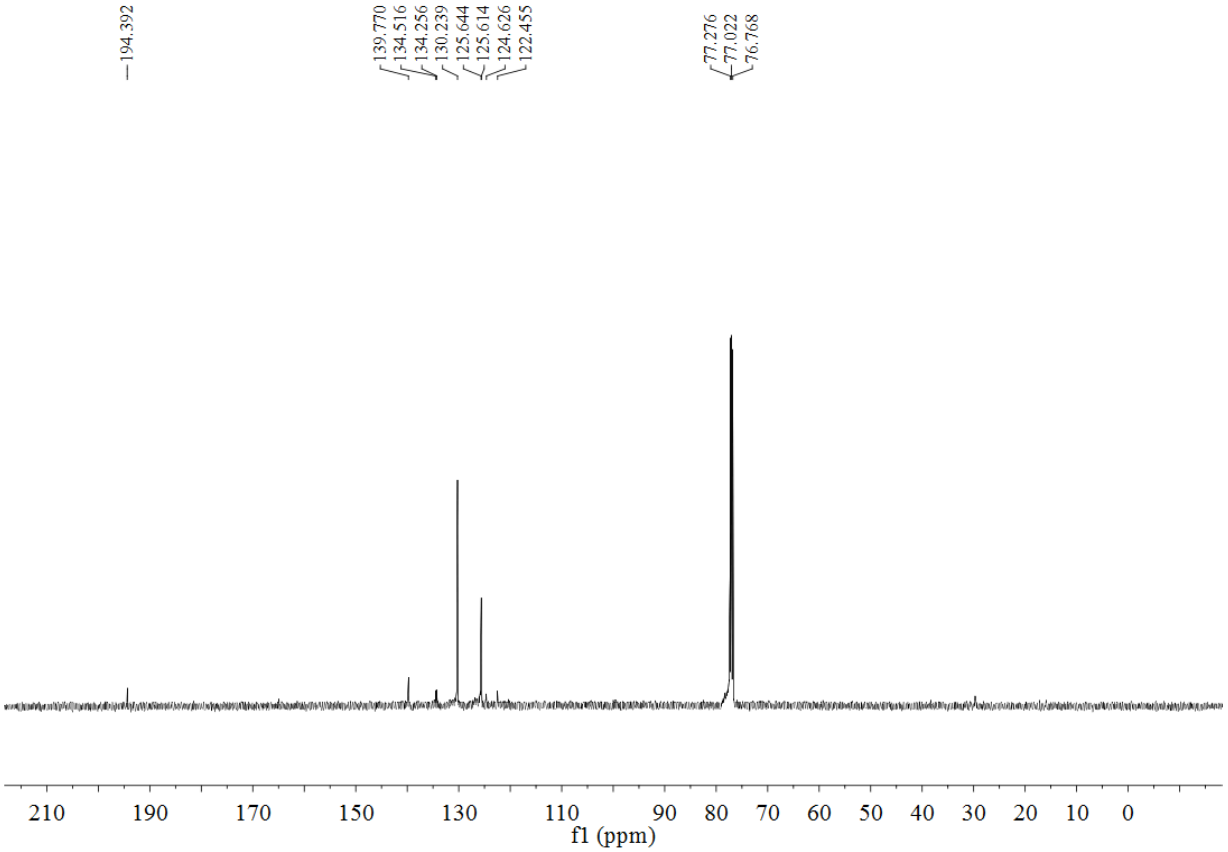
194.392
139.770
134.516
134.256
130.239
125.644
125.614
124.626
122.455
77.276
77.022
76.768
210 190 170 150 130 110 90 80 70 60 50 40 30 20 10 0
f1 (ppm)

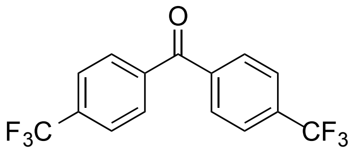
O
F3C
CF3

4k

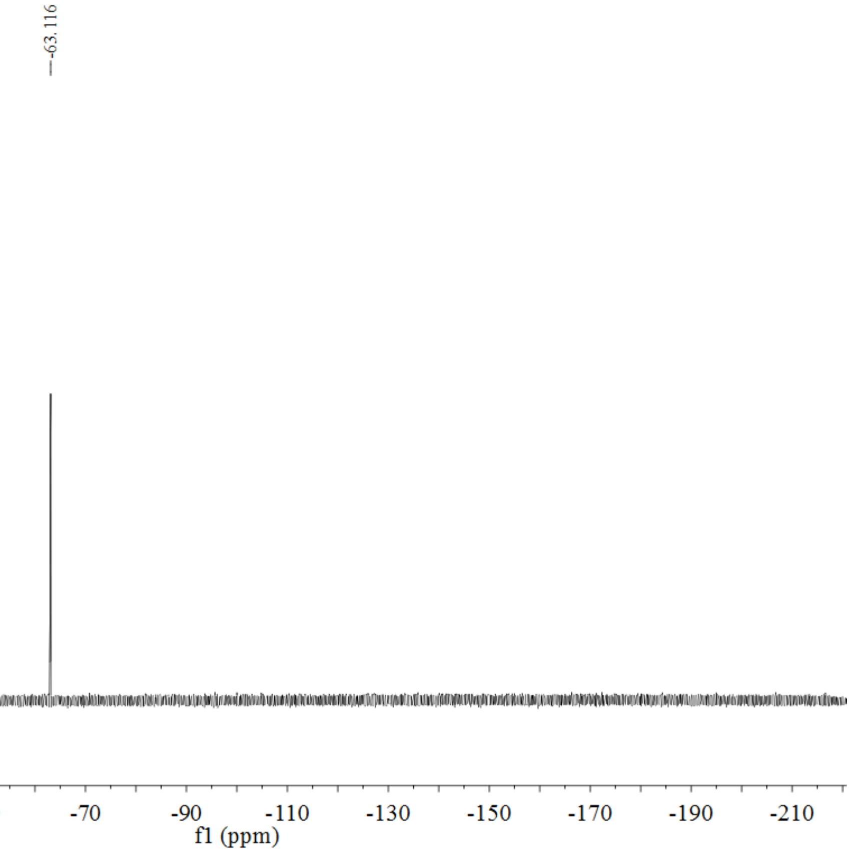
-63.116
20
10
0
-10
-30
-50
-70
-90
-110
-130
-150
-170
-190
-210
f1 (ppm)

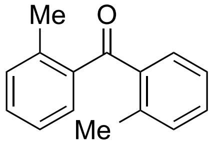

4l

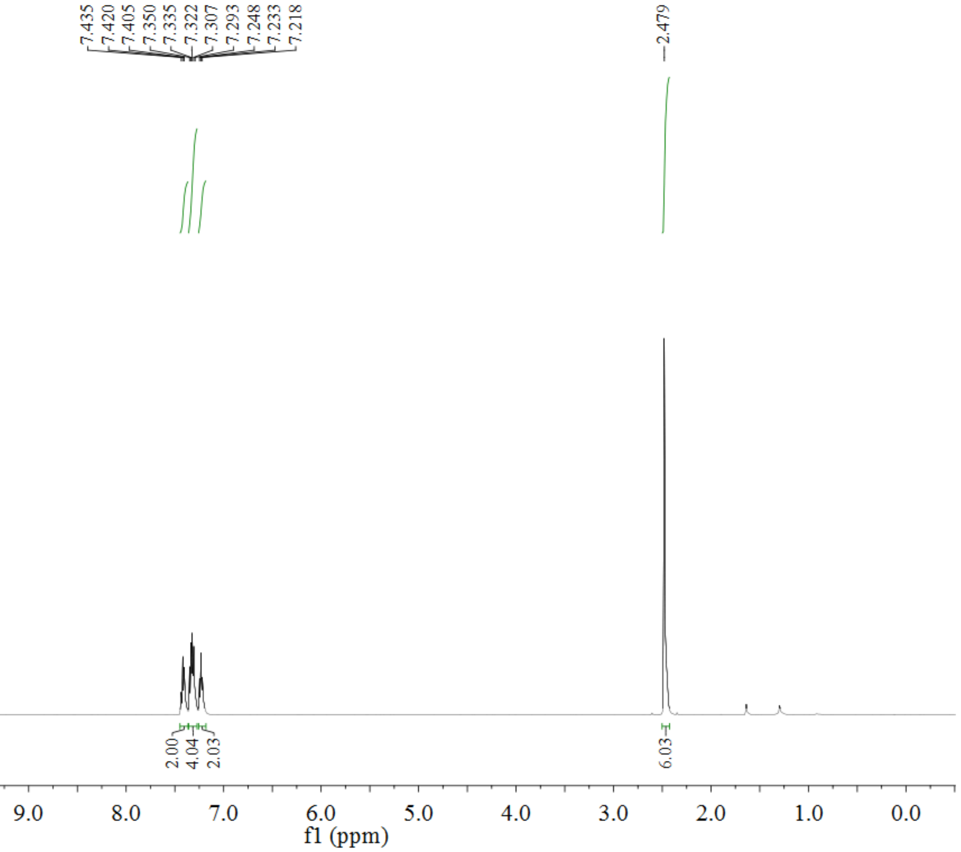
7.435
7.420
7.405
7.350
7.335
7.322
7.307
7.293
7.248
7.233
7.218
2.479
2.00
4.04
2.03
6.03
11.0 10.0 9.0 8.0 7.0 6.0 5.0 4.0 3.0 2.0 1.0 0.0
f1 (ppm)

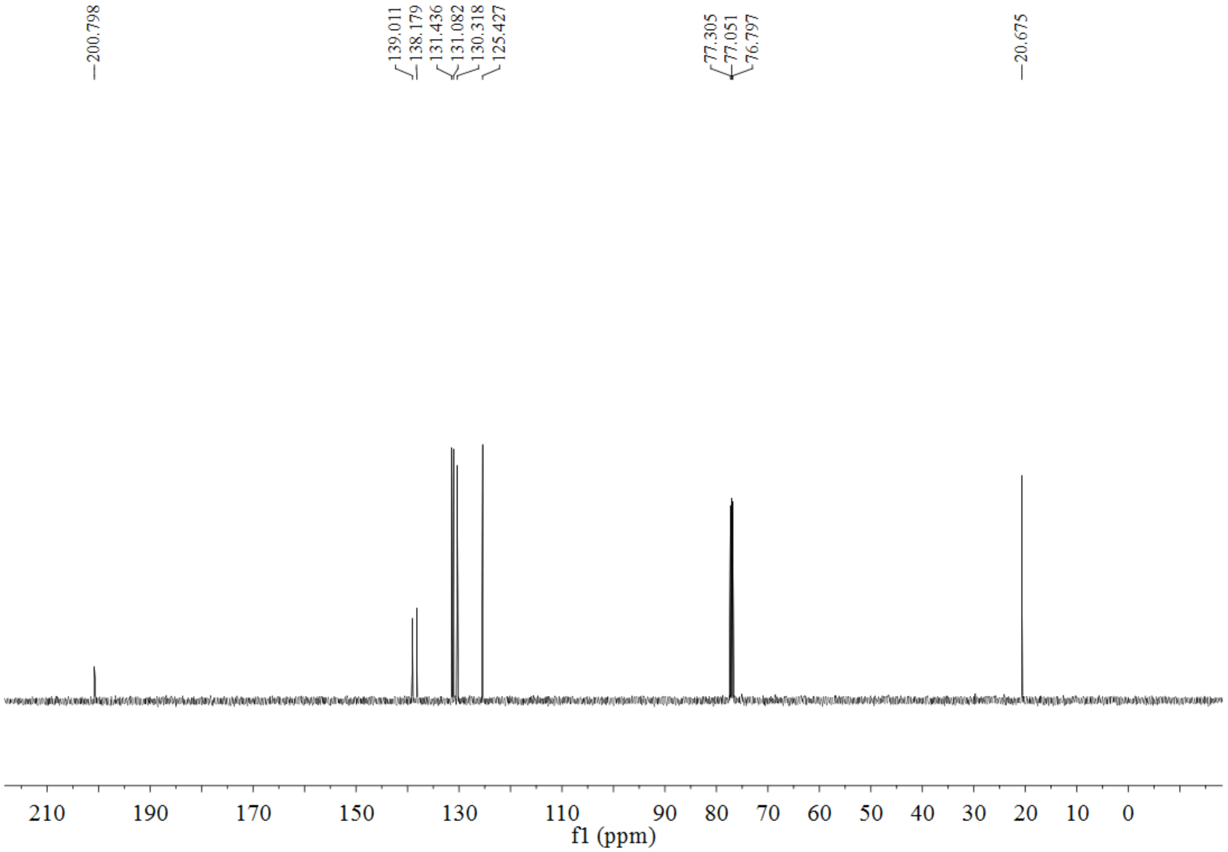
200.798
139.011
138.179
131.436
131.082
130.318
125.427
77.305
77.051
76.797
20.675
210 190 170 150 130 110 90 80 70 60 50 40 30 20 10 0
f1 (ppm)

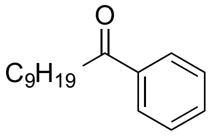
O
C_9H_{19}

4m

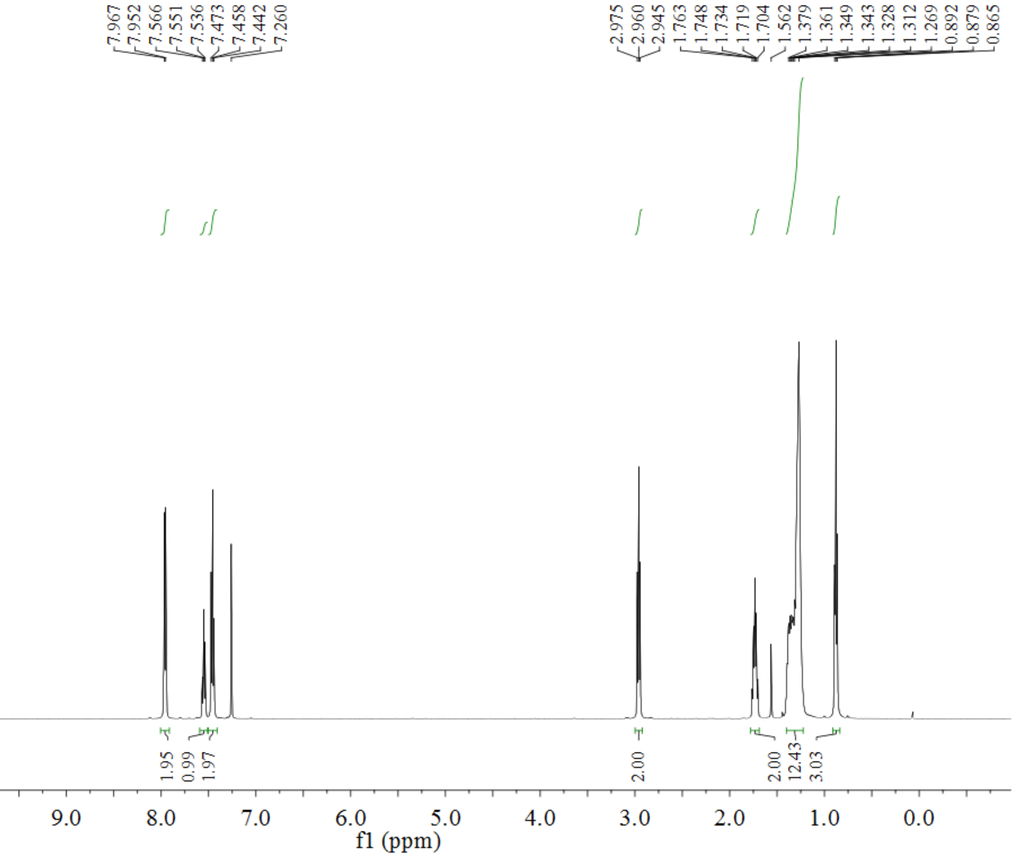
7.967
7.952
7.566
7.551
7.536
7.473
7.458
7.442
7.260
2.975
2.960
2.945
1.763
1.748
1.734
1.719
1.704
1.562
1.379
1.361
1.349
1.343
1.328
1.312
1.269
0.892
0.879
0.865
1.95
0.99
1.97
2.00
2.00
12.43
3.03
11.0
10.0
9.0
8.0
7.0
6.0
5.0
4.0
3.0
2.0
1.0
0.0
f1 (ppm)

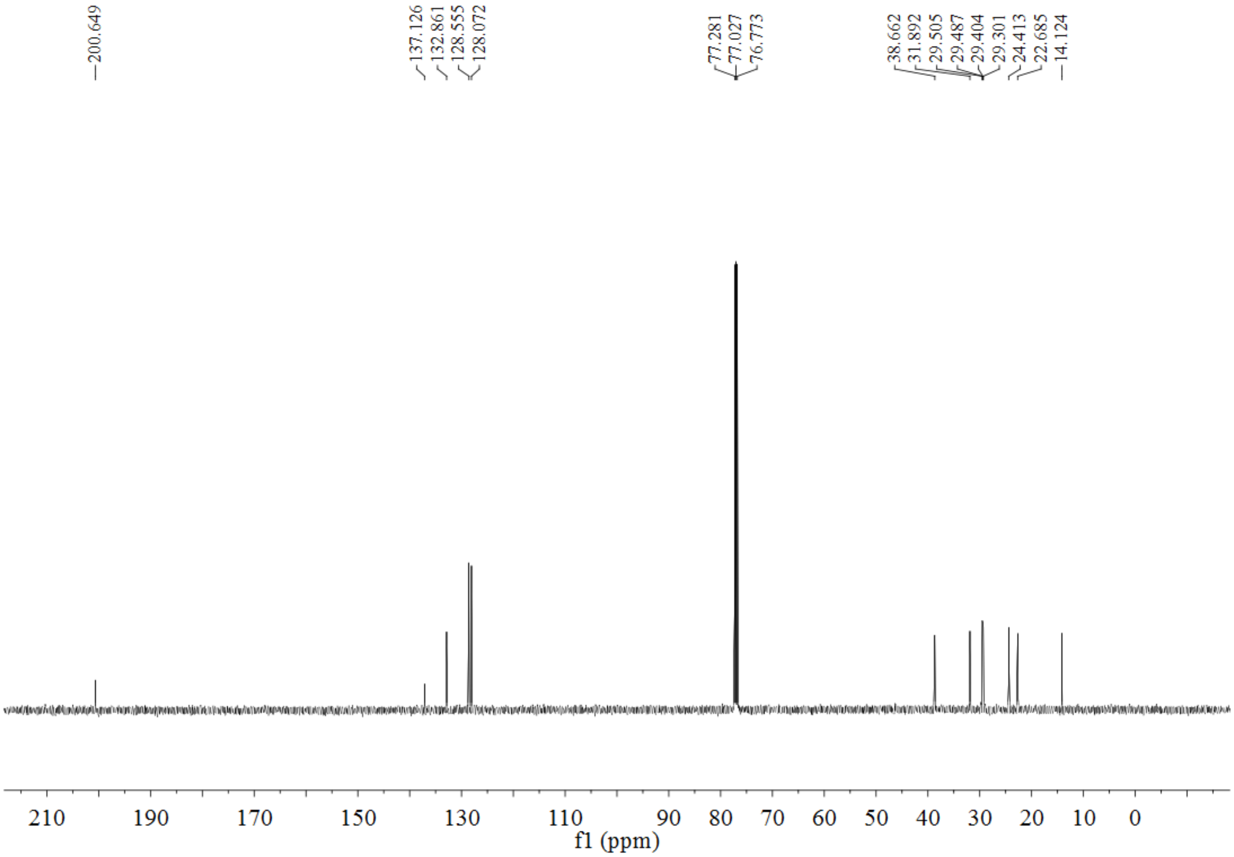
200.649
137.126
132.861
128.555
128.072
77.281
77.027
76.773
38.662
31.892
29.505
29.487
29.404
29.301
24.413
22.685
14.124
210
190
170
150
130
110
90
80
70
60
50
40
30
20
10
0
f1 (ppm)
